# Caesarean section rates and obstetric practices in Sierra Leone before, during, and after the Ebola epidemic: Results from a nationwide household study

**DOI:** 10.1177/17455057261487141

**Published:** 2026-09-21

**Authors:** Josien Westendorp, Hanna M. Mathéron, Alex J. van Duinen, Momoh L. Mansaray, Lora Daskalska, Osman Sankoh, Risa Hoffmann-Lonnée, Reinou S. Groen, Martin P. Grobusch, Håkon A. Bolkan

**Affiliations:** 1 Department of Public Health and Nursing, Norwegian University of Science and Technology, Trondheim, Norway; 2 CapaCare NGO, Sierra Leone, Liberia, The Netherlands, Trondheim, Norway; 3 Department of Obstetrics and Gynaecology, St. Olavs Hospital HF, Trondheim University Hospital, Trondheim, Norway; 4 Center of Tropical Medicine and Travel Medicine, Amsterdam University Medical Centres, AMC, Amsterdam Infection & Immunity, Amsterdam Public Health – Global Health, University of Amsterdam, Amsterdam, The Netherlands; 5 612607 Masanga Medical Research Unit, Masanga, Sierra Leone; 6 Department of Surgery, St. Olavs Hospital HF, Trondheim University Hospital, Trondheim, Norway; 7 Institute for Health and Society and Epidemiology Division, 5506 Medical College of Wisconsin, Milwaukee, WI, USA; 8 380727 Centre for Health Research and Training, University of Management and Technology, Freetown, Sierra Leone; 9 School of Community College of Health Sciences, Njala University, Bo Campus, Bo, Sierra Leone; 10 School of Public Health, Faculty of Health Sciences, University of the Witwatersrand, Johannesburg, South Africa; 11 Alaska Native Medical Centre, Anchorage, AK, USA; 12 Alaska Native Tribal Health Consortium – Southcentral Foundation (ANTHC/SCF), Anchorage, AK, USA; 13 Centre de Recherches Médicales en Lambaréné (CERMEL), Lambaréné, Gabon; 14 Institute of Molecular Medicine and Infectious Diseases, University of Cape Town, Cape Town, South Africa; 15 Institut für Tropenmedizin und Deutsches Zentrum für Infektiologie (DZIF), Universität Tübingen, Tübingen, Germany

**Keywords:** Sierra Leone, caesarean section, reproductive health, childbirth, assisted vaginal delivery, household survey, maternal mortality, Ebola (EBV) outbreak, education

## Abstract

**Background:**

Through health system reforms Sierra Leone has achieved reductions from 1,603 to 354 maternal deaths per 100,000 live births between 2000 and 2023, but progress has been uneven and severely disrupted by the 2013–2016 Ebola virus disease (EVD) outbreak. National surveys track key maternal health indicators but provide limited insight into assisted vaginal delivery and women’s access to emergency obstetric care, including caesarean section, during and after health system shocks.

**Objectives:**

To describe patterns of caesarean section and assisted vaginal delivery, place and mode of transport for childbirth before, during, and after the EVD outbreak from a community perspective, and assess changes compared with earlier nationwide household study data.

**Design:**

Cross-sectional household survey.

**Methods:**

A district and urban-rural weighted random cluster design selected enumerator areas with probability proportional to population size. Women aged 12–50 years provided obstetric histories. Data were compared with the 2012 household survey using the same methodology and interpreted in the context of national health system initiatives and the EVD outbreak timeline. Descriptive statistics and chi-square tests examined differences across time periods.

**Results:**

Among 914 women with 2,094 pregnancies, caesarean section rates rose from 1.6% pre-to 3.7% post-EVD, while assisted vaginal delivery dropped to zero during the outbreak and re-emerged at 2.9%. Facility births increased from 57.6% pre-to 73.8% post-EVD, with home deliveries declining to 16.3%. Almost half of women delivering post-EVD reached facilities on foot, and ambulance use remained minimal. No substantial improvement was observed in girls’ education and literacy over the decade.

**Conclusion:**

Obstetric service use in Sierra Leone continued to improve after the EVD outbreak, but transport barriers and low assisted vaginal delivery and caesarean section rates persist. Strengthening girls’ education, referral systems, emergency obstetric capacity and skills retention remain essential for resilient, equitable maternal care.

## Introduction

Ensuring access to timely emergency obstetric interventions, including assisted vaginal delivery (AVD) and caesarean section (C-section), is fundamental to meeting Sustainable Development Goal 3.1, to reduce the global maternal mortality ratio to less than 70 per 100,000 live births by 2030.^1^ Sierra Leone has reduced its maternal mortality rate from 1,603 in 2000 to 354 maternal deaths per 100,000 live births in 2023.^2^

The 2013–2016 Ebola viral disease (EVD) outbreak severely disrupted Sierra Leone’s health system, including maternal and newborn services. Multiple studies indicate that antenatal care attendance, institutional childbirth, and emergency obstetric services (including C-section) declined sharply during the epidemic and recovered partially in due course.^3,4^ Major health system reforms have been implemented before and after the EVD epidemic, including the Free Health Care Initiative (FHCI) introduced in 2010 which alleviated health care user fees for pregnant and lactating women and the introduction of fines for home deliveries.^5,6,7^ Substantial expansion of the health workforce through the training of midwives and surgical associate clinicians to provide emergency obstetric care, including AVD and C-sections, together with the establishment of the National Emergency Medical Service (NEMS) in 2018, providing free ambulance services, have also been important.^8–10^ These initiatives have improved access to emergency obstetric care, but geographic, financial, sustainability, and quality-of-care challenges persist.^11,8,12,13^

Three Demographic Household Surveys (DHS) have been conducted in Sierra Leone over the past two decades; with the last two in 2013 and 2019, one year before and four years after the EVD outbreak, corresponding to time periods assessed in this study. Between 2013 and 2019 the C-section rate increased from 2.9 to 4.1%, facility births from 54.0 to 83.0% and births attended by a skilled provider from 60.0 to 86.9%.^14,15^ However, the DHS provides no data on AVD, transport to childbirth facilities, or household-level data on obstetric care during the EVD outbreak.

In this context, analysing patterns of obstetric care before, during, and after the EVD outbreak period may provide important insights into health system resilience. This study aimed to compare C-section and AVD rates, mode of transport, and place of delivery from a community perspective before, during and after the EVD outbreak.

## Materials and methods

### Study setting

Sierra Leone is a low-income West-African country with a population of 8.4 million, ranked 185 out of 193 on the 2025 Human Development Index.^16^ Primary care and basic obstetric services are mainly provided in peripheral health units (PHUs). Most of them have a simple labour room, but no operating theatre. Emergency obstetric surgical care is largely provided in governmental and non-governmental hospitals, with a few additional private hospitals and clinics.^17^ Health care services were severely disrupted by the civil war in the nineties and the West-African EVD outbreak from 2013 to 2016.^4,18^ The government of Sierra Leone has prioritised obstetric care over the last two decades. In addition to the previously described FHCI, NEMS, and the deployment of nearly 2,000 midwives and more than 100 surgically trained associate clinicians, Sierra Leone introduced a maternal death surveillance and response system in 2015, conducted regular nationwide emergency obstetric and newborn care assessments, and developed national reproductive, maternal, newborn, and child health strategic plans in 2008, 2017, and 2024.^19–22^

### Study design

This population-based, cross-sectional household study of obstetric care was part of a larger household survey examining prevalence of surgical conditions in Sierra Leone (PRESSCO 2020). The survey design and sampling strategy have previously been described in detail.^23^ In brief, a weighted multistage random cluster sampling design was used to obtain a nationally representative sample. Enumerator Areas (EAs) were selected with a probability proportional to the population size. Statistics Sierra Leone staff with DHS experience provided the maps and the EAs, performed the randomization, and supervised data collection in all clusters onsite. To obtain a representative sample of the population, each district as well as the rural and urban areas were stratified. A random calculator assigned 25 households per EA. Data was collected on password protected tablets with 3G connection in REDCap^©^ (Research Electronic Data Capture) between October 16^th^ and November 11^th^, 2019, and February 15^th^ to March 7^th^, 2020. Enumerators were all health care workers, extensively trained in randomisation, experienced in surveying and interviewing, as well as in applying the survey tool.

Women aged 12–50 years, residing and randomised in the selected households at interview, who either reported one or more menstruation periods in the past year or were amenorrhoeic for more than one year because of pregnancy or hysterectomy were included for this study on women’s health.^23^ Women outside the 12–50 age range or amenorrhoeic without pregnancy/hysterectomy were excluded. A referral letter was issued when medical conditions in need of attention were discovered and verified by one of the medical staff on site.

The PRESSCO questionnaire was adapted from the validated Surgeons OverSeas Assessment of Surgical Need (SOSAS) tool.^23^ The adapted version was modified to include obstetric-specific variables relevant to the study objectives and context and was not formally validated (supplemental material). Prior to data collection, the questionnaire was pilot tested in a small number of households outside the main sampling areas to assess clarity, cultural relevance, and feasibility of administration. Minor revisions were made based on feedback from this process.

Data from the 2020 survey were compared with findings from the SOSAS conducted in Sierra Leone in 2012.^24,25^ While both studies used stratified cluster-based sampling approaches, there were differences in survey instruments and variable categorisation. For example, the 2020 survey included additional categories for place of delivery (e.g., traditional birth attendant and “other”), which were not explicitly captured in the 2012 dataset. For comparative analyses, categories were harmonised where possible by grouping place of delivery into broader categories (e.g., home vs facility-based delivery).

### Outcomes

The women’s health interview included a full obstetrical history on current and previous pregnancies. Pregnant women were interviewed about complaints in their current pregnancy but were not independently verified through clinical records. Fundal height and blood pressure were measured. An AVD was defined as a birth accomplished using instruments such as vacuum extractor or obstetric forceps device after the onset of the second stage of labour. C-section was defined as an abdominal delivery of a foetus through surgical incision of the uterus. The mode of transport was categorised as walking, motorcycle, (public) car/taxi, boat or ambulance. Facility-based delivery included births occurring in public or private health facilities. Indicators were compared between before, during and after the EVD outbreak, whereby the EVD outbreak was defined as the period from the first confirmed case in Sierra Leone (May 2014) to the official declaration of the end of the outbreak (November 2016) in the country. Findings of this study undertaken in 2019 and 2020 were compared with the SOSAS from 2012.^24^

Household head respondents were asked whether any woman of reproductive age in the household had died while pregnant or within six weeks after her delivery, within the preceding year. These data were based on retrospective self-reporting and were limited to deaths occurring in the 12 months prior to the interview. Given the design of the survey, maternal deaths could not be independently verified, and detailed clinical or temporal classification relative to the EVD outbreak (before, during, or after) was not possible. Therefore, these data are presented for descriptive purposes only.

### Analysis

Descriptive statistics including frequencies and measures of central tendency were used to describe the PRESSCO 2020 and SOSAS 2012 samples. We compared categorical data for age, residency, education, literacy, self-reported health, occupation, and family planning methods between the two surveys, as well as before, during and after the EVD outbreak using Chi Square Tests (alpha<0.05). All SOSAS and PRESSCO data was gathered from the original data files. Stata IC 15.1 was used for data analysis. Analyses of pregnancies before, during, and after the EVD outbreak were restricted to data from the PRESSCO 2020 survey, as the SOSAS 2012 questionnaire did not capture lifetime pregnancies. This approach ensured internal consistency in data collection, variable definitions, and recall structure, as all pregnancies were reported by the same respondents using a uniform questionnaire.

The sample size for PRESSCO 2020 was determined based on the primary outcomes of the parent study (prevalence of surgical conditions), rather than obstetric-specific outcomes. As such, while the sample is sufficient for national-level estimates of common outcomes, it may be underpowered for subgroup analyses and for less frequent events such as C-section and AVDs. We did not apply survey weights or accounted for clustering in the statistical analyses, as the analyses focused on observed sample proportions rather than generating nationally representative population estimates.

### Reporting guideline, consent and ethical approval

The reporting of this study conforms to the STROBE statement for cross-sectional studies (Supplemental Material).^26^ Permission was obtained from community leaders before enumeration. Literate participants provided written informed consent; for those unable to read or write, the form was read aloud in the local language, witnessed verbal consent was obtained, and consent was documented by thumbprint. Participants aged 12–18 years provided assent alongside parental or guardian consent. Participants were informed that their anonymised data could help improve health services, with further details provided in the supplementary questionnaire. The protocol was evaluated by the Masanga Medical Research Unit’s Scientific Review Committee (MMRU-SRC-009-2019), and ethical clearance was granted by the Norwegian Regional Committee for Medical and Health Research Ethics (REC 2019/31932) as well as the Sierra Leone Ethics and Scientific Review Committee (SLESRC 2019/October/03). The study was registered with the ISRCTN registry (No. 12353489).

## Results

In the 75 EAs visited, 1,854 households and 3,618 household members were included. All 914 women in the reproductive age, consented for an in-depth interview on women’s health (Table 1). Participants’ median age was 25 years (range 12–48). Rural residence predominated (59.3%, 542/914), 46.3% (423/914) of the women had no schooling, while 58.3% (533/914) reported themselves as illiterate. Farming (30.6%, 280/914) and self-employment (23.9%, 218/914) were the most common occupations, whereas 18.3% (167/914) were unemployed. Contraception was used by 261/914 women (28.6%). Of all women included in the study, 652/914 (71.3%) had been pregnant before and 73/914 (7.9%) were pregnant at the time of the survey. The cohort recalled 2,094 lifetime pregnancies that produced 1,990 births, divided between 95.1% (1,892/1,990) spontaneous vaginal deliveries, 2.3% (46/1,990) C-sections and 2.3% (45/1,990) AVDs (Table 2).

**Table 1.** Socioeconomic characteristics of female PRESSCO 2020 survey respondents in comparison with reference national household survey SOSAS 2012.

​	PRESSCO 2020		SOSAS 2012*		Difference *p*-value†
	n	% (95% CI)	n	% (95% CI)	
Total respondents	914	100.0	990	100.0	​
**Age**	​	​	​	​	**0.006**
12-17 years	141	15.4 (13.1–17.9)	145	14.6 (12.5–17.0)	​
18-25 years	340	37.2 (34.1–40.4)	355	35.9 (32.9–38.9)	​
26-35 years	300	32.8 (29.8–36.0)	305	30.8 (27.9–33.8)	​
36-40 years	95	10.4 (8.5–12.6)	110	11.1 (9.2–13.2)	​
41-50 years	33	3.6 (2.5–5.0)	75	7.6 (6.0–9.4)	​
Missing	5	0.5 (0.2–1.3)	​	​	​
**Residency**	​	​	​	​	0.129
Urban	372	40.7 (37.5–44.0)	437	44.1 (41.0–47.3)	​
Rural	542	59.3 (56.0–62.5)	553	55.9 (52.7–59.0)	​
Missing	0	0 (0.0–0.04)	0	0 (0.0–0.04)	​
**Education**	​	​	​	​	0.208
None	423	46.3 (43.0–49.6)	500	50.5 (47.3–53.7)	​
Primary school	119	13.0 (10.9–15.4)	116	11.7 (9.8–13.9)	​
Secondary school or higher	365	39.9 (36.7–43.2)	371	37.5 (34.5–40.6)	​
Missing	7	0.8 (1.6-3.1)	3	0.3 (0.1–0.8)	​
**Literacy**	​	​	​	​	**0.031**
Not able to read or write	533	58.3 (55.0–61.5)	532	53.7 (50.6–56.9)	​
Able to read and write	372	40.7 (37.5–44.0)	454	45.9 (42.7–49.0)	​
Missing	9	1.0 (0.5–1.9)	4	0.4 (0.1–1.0)	​
**Self-reported health**	​	​	​	​	**<0.001**
Healthy	808	88.4 (86.1–90.4)	575	58.1 (54.9–61.2)	​
Un-healthy	91	10.0 (8.1–12.1)	413	41.7 (38.6–44.9)	​
Missing	15	1.6 (0.9–2.7)	2	0.2 (0.0–0.7)	​
**Occupation**	​	​	​	​	**0.008**
Farmer	280	30.6 (27.7–33.7)	310	31.3 (28.4–34.3)	​
Unemployed	167	18.3 (15.8–20.9)	267	27.0 (24.2–29.9)	​
Self-employed	218	23.9 (21.1–26.8)	233	23.5 (20.9–26.3)	​
Home worker	96	10.5 (8.6–12.7)	147	14.8 (12.7–17.2)	​
Employee	23	2.5 (1.6–3.8)	32	3.2 (2.2–4.5)	​
Student	118	12.9 (10.8–15.3)	​	​	​
Other	5	0.6 (0.2–1.3)	​	​	​
Missing	7	0.8 (0.3–1.6)	1	0.1 (0.0–0.6)	​
**Family planning**	261	100.0	235	100.0	**<0.001**
Contraceptive pills	59	22.6 (17.7–28.2)	88	37.4 (31.2–44.0)	​
Implant	97	37.2 (31.3–43.3)	20	8.5 (5.3–12.8)	​
Injectable	97	37.2 (31.3–43.3)	111	47.2 (40.7–53.8)	​
Other	6	2.3 (0.8–4.9)	16	6.8 (3.9–10.8)	​
Don’t know	2	0.8 (0.1–2.7)	​	​	​

*Surgeons OverSeas Assessment of Surgical Needs (SOSAS) national data collection in Sierra Leone in 2012.

^†^Missing category was not used in Chi^2^ test. Statistically significant differences in bold. 95% CI = 95% Confidence Interval.

**Table 2.** Recalled lifetime pregnancies, location and type of delivery from PRESSCO 2020 compared with SOSAS 2012.

​	PRESSCO 2020		SOSAS 2012*		Difference *p*-value†
	n	% (95% CI)	n	% (95% CI)	
**Recalled lifetime pregnancies**	2,094	100.0	2,645	100.0	N/A
Total deliveries	1,990	95.0 (94.0–95.9)	2,316	87.6 (86.2–88.8)	**<0.001**
**Location for deliveries**	​	​	​	​	**<0.001**
Home	445	22.4 (20.5–24.3)	1,355	58.5 (56.5–60.5)	​
Facility based deliveries	1,280	64.3 (62.2–66.4)	957	41.3 (39.3–43.4)	​
Other	261	13.1 (11.7–14.7)	0	0.0 (0.0–0.2)	​
Missing	4	0.2 (0.1–0.05)	4	0.2 (0.0–0.4)	​
**Type of delivery**	​	​	​	​	**<0.001**
Caesarean sections	46	2.3 (1.7–3.1)	44	1.9 (1.4–2.5)	​
Assisted vaginal deliveries	45	2.3 (1.7–3.0)	9	0.4 (0.2–0.7)	​
Spontaneous vaginal deliveries	1,892	95.1 (94.0–96.0)	2,263	97.7 (97.0–98.3)	​
Missing	7	0.4 (0.1–0.7)	0	0.0 (0.0–0.2)	​

*Surgeons OverSeas Assessment of Surgical Needs (SOSAS) national data collection in Sierra Leone in 2012.

^†^Missing category was not used in Chi^2^ test. N/A=not available. Statistically significant differences in bold. 95% CI = 95% Confidence Interval.

### PRESSCO 2020 vs. SOSAS 2012 (Table 1 and 2)

Residencies in PRESSCO were identical to the SOSAS eight years earlier (^24^, Table 1). Self-reported health improved markedly; 88.4% (808/914) in 2020 considered themselves healthy compared with 58.1% (575/990) in 2012. Unemployment declined from 27.0% (267/990) to 18.3% (167/914). The findings indicate that female illiteracy rates were higher in 2020 than in 2012, with no statistically significant improvements in educational attainment observed (Table 1). Modern family-planning uptake rose both numerically and proportionally. Method choice shifted towards long-acting options: implants increased, pills and injectables decreased.

Between 2012 and 2020 home deliveries declined markedly and facility deliveries increased similarly (Table 2). Of all facility deliveries, 63.2% (809/1,280) took place in peripheral health units and 36.8% (471/1,280) in hospitals in 2020.

### Assisted vaginal deliveries & C-sections before, during and after the EVD outbreak (Table 3)

Of the 2,094 recalled pregnancies in 2020, 63.9% (1336/2,094), 7.9% (166/2,094) and 28.1% (588/2,094) was categorised as before, during and after the EVD outbreak (Table 3). Compared against pre-EVD outbreak, there was a significant change in mode of delivery during and after. The C-section rate increased from 1.6% (95% CI 1.0–2.5; 21/1279) before EVD to 3.3% (95% CI 1.1–7.5; 5/153) during and 3.7% (95% CI 2.3–5.6; 20/545) after. AVD disappeared entirely during the outbreak but increased from 2.3% (95% CI 1.5–3.2; 29/1279) to 2.9% (95% CI 1.7–4.7; 16/545) of the deliveries before and after the EVD.

**Table 3.** Pregnancy characteristics before, during and after the EVD outbreak in the PRESSCO 2020.

​	Total		Before EVD		During EVD			After EVD		
	n	%	n	%	n	%	*p*-value*	n	%	*p*-value*
Total pregnancies	2,094^†^	100.0	1,336	63.9	166	7.9	​	588	28.1	​
Total deliveries	1,990^††^	100.0	1,279	64.7	153	7.7	​	545	27.6	​
Total babies	2,152^§^	100.0	1,368	64.2	167	7.8	​	596	28.0	​
**Type of pregnancy**	​	​	​	​	​	​	0.342	​	​	0.276
Singleton	1,983	94.7	1,274	95.4	155	93.4	​	554	94.2	​
Twin	51	2.4	34	2.5	4	2.4	​	13	2.2	​
Triplet	1	0.0	1	0.1	0	0.0	​	0	0.0	​
Unknown	54	2.6	27	2.0	7	4.2	​	20	3.4	​
Missing	5	0.2	0	0.0	0	0.0	​	1	0.2	​
**Duration of gestation of pregnancy**	​	​	​	​	​	​	0.243	​	​	**<0.001**
Preterm	153	7.3	85	6.4	15	9.0	​	53	9.0	​
At-term	1,526	72.9	1,017	76.1	115	69.3	​	394	67.0	​
Post-term	389	18.6	222	16.6	34	20.5	​	133	22.6	​
Unknown	20	1.0	10	0.7	2	1.2	​	8	1.4	​
Missing	6	0.3	2	0.1	0	0.0	​	0	0.0	​
**Transportation to health facility**	​	​	​	​	​	​	0.002	​	​	**<0.001**
Ambulance	25	1.2	10	0.7	4	2.4	​	11	1.9	​
Boat	8	0.4	2	0.1	0	0.0	​	6	1.0	​
On foot	896	42.8	557	41.7	78	47.0	​	261	44.4	​
Motorcycle	301	14.4	170	12.7	21	12.7	​	110	18.7	​
Public transport (bus/taxi)	124	5.9	75	5.6	11	6.6	​	38	6.5	​
Car/Taxi/Vehicle	140	6.7	91	6.8	9	5.4	​	40	6.8	​
Home delivery (no transport)	558	26.6	417	31.2	36	21.7	​	105	17.9	​
Other	3	0.1	0	0.0	1	0.6	​	2	0.3	​
Multiple types of transportation	26	1.2	9	0.7	4	2.4	​	13	2.2	​
Missing	13	0.6	5	0.4	2	1.2	​	2	0.3	​
**Location for delivery**	​	​	​	​	​	​	0.056	​	​	**<0.001**
Home	498	23.8	366	27.4	36	21.7	​	96	16.3	​
Peripheral Health Unit	827	39.5	460	34.4	75	45.2	​	292	49.7	​
Hospital	487	23.3	309	23.3	36	21.7	​	142	24.1	​
At traditional birth attendant	255	12.2	187	12.2	16	9.6	​	52	8.8	​
Other	17	0.8	10	0.8	2	1.2	​	5	0.9	​
Missing	10	0.5	4	0.5	1	0.6	​	1	0.2	​
**Mode of delivery**	​	​	​	​	​	​	0.065	​	​	0.019
Spontaneous vaginal delivery	1,886	94.8	1,229	96.1	148	96.7	​	509	93.4	​
Assisted vaginal delivery	45	2.03	29	2.3	0	0	​	16	2.9	​
Caesarean section	46	2.3	21	1.6	5	3.3	​	20	3.7	​
Missing	13	0.7	0	0.0	0	0.0	​	0	0.0	​
**Reported reason for C-sections**	​	​	​	​	​	​	0.045	​	​	**0.013**
Bleeding before birth	1	2.2	1	4.8	0	0.0	​	0	0.0	​
Foetal distress	8	17.4	7	33.3	0	0.0	​	1	5.0	​
Malpresentation	10	21.7	0	0.0	2	40.0	​	8	40.0	​
Multiple pregnancies	2	4.3	1	4.8	0	0.0	​	1	5.0	​
Obstructed labour	16	34.8	8	38.1	3	60.0	​	5	25.0	​
Previous surgery (including C-sections)	2	4.3	0	0.0	0	0.0	​	2	10.0	​
Unknown	6	13.0	3	14.3	0	0.0	​	3	15.0	​
Missing	1	2.2	1	4.8	0	0.0	​	0	0.0	​
**Outcome of delivery**	​	​	​	​	​	​	0.235	​	​	0.078
Miscarriage/abortion	60	2.8	31	2.3	9	5.4	​	20	3.4	​
Late miscarriage^¶^	44	2.0	23	1.7	4	2.4	​	17	2.9	​
Alive	1,890	87.8	1,227	89.7	145	86.8	​	518	86.9	​
Perinatal mortality	106	4.9	72	5.3	7	4.2	​	27	4.5	​
Unknown	4	0.2	2	0.1	0	0.0	​	2	0.3	​
Other	27	1.3	13	1.0	2	1.2	​	12	2.0	​
Missing	21	1.0	0	0.0	0	0.0	​	0	0.0	​
**Self-reported complications**	​	​	​	​	​	​	0.330	​	​	**0.005**
Severe bleeding	88	4.2	41	3.1	7	4.2	​	40	6.8	​
Infection/Fever	20	1.0	9	0.7	4	2.4	​	7	1.2	​
Perineal tear	1	0.0	0	0.0	0	0.0	​	1	0.2	​
No menstrual period	3	0.1	1	0.1	0	0.0	​	2	0.3	​
Different bleeding period	5	0.2	4	0.3	0	0.0	​	1	0.2	​
Other	41	2.0	27	2.0	4	2.4	​	10	1.7	​
Unknown	3	0.1	2	0.1	0	0.0	​	1	0.2	​
No complication	1,922	91.8	1,249	93.5	150	90.4	​	523	88.9	​
Missing	11	0.5	3	0.2	1	0.6	​	3	0.5	​

C-section = Caesarean section. EVD = Ebola viral disease.

*Chi^2^ test comparing between mentioned timeframe and before Ebola, missing category not used. N/A=not available.

^†^For 4 pregnancies period was unknown.

^††^For 13 deliveries period was unknown.

^§^for 21 babies period was unknown.

^¶^Died between 3-6 months of gestation. Statistically significant results in bold.

### Location of delivery and transport before, during and after the EVD outbreak (Table 3)

The trend observed when comparing 2012 with 2020, was also found when observing obstetric practices around the EVD outbreak (Table 3). Before EVD, more than a quarter (366/1,336, 27.4%) of deliveries occurred at home, and two in five women reached care facilities on foot (557/1,336, 41.7%). Motorised travel was limited to motorcycles (170/1,336, 12.7%), cars/taxis (91/1,336, 6.8%) and ambulances (10/1,336, 0.7%). During the EVD outbreak, both location of delivery and transport to health facility changed. Home deliveries decreased from 27.4% (366/1,336) before the EVD outbreak to 21.7% (36/166) during the EVD outbreak and facility delivery rose to 66.9% (111/166), up from 57.6% (769/1,336) before the EVD outbreak. Walking became even more common (47.0%, 78/166) and ambulance uptake went up to 2.4% (4/166), and although small in numbers, this might possibly reflect longer, emergency-driven referral routes. After the EVD outbreak, the shift to facilities consolidated: 73.8% (434/588) of the births occurred in peripheral health units (PHUs) or hospitals, leaving home deliveries at 16.3% (96/588). Walking declined slightly, while motor-cycle transport expanded to 18.7% (110/588) and cars/taxis returned to pre-EVD outbreak figures. Ambulance use, however, remained marginal, and nearly half of post-Ebola patients still walked. The survey did not directly establish why women walked or whether geographic or financial barriers accounted for this pattern.

### Outcomes (Table 4)

The household representatives reported that in the year preceding the study, 12 females in reproductive age in the households died while being pregnant or within 6 weeks after delivery (Table 4), nearly five-fold lower than 2012 SOSAS (53 deaths of females in reproductive age reported). Three out of these 12 women delivered by C-sections with the indications reported to be: hypertension, prolonged/obstructed labour and sickle cell disease.

**Table 4.** Maternal deaths in the household, 1 year preceding the interview.

​	N	%
Maternal deaths in the last year*	12	100
**Complication in pregnancy**		
Bleeding	3	25.0
Prolonged/obstructed labour	2	16.7
Convulsions	1	8.3
Hypertension	1	8.3
Other	3	25.0
Unknown	2	16.7
**Caesarean section**		
Yes	3	25.0
No	9	75.0
**Health seeking behaviour**		
Health care facility	10	83.3
Traditional healer	3	25.0
Neither	1	8.3
**Location of death**		
Home	7	58.3
Health Clinic	2	16.7
Hospital	3	25.0

*Females in reproductive age in the households died while being pregnant or within 6 weeks after delivery.

Two women died in hospital, while one woman died at home following the C-section performed for prolonged/obstructed labour.

## Discussion

This community-based survey identified differences in obstetric care practices among pregnancies occurring before, during, and after the EVD epidemic. Three principal findings emerged. Firstly, the proportion of births by C-section was higher in the post-EVD period than in the pre-EVD period, while no assisted vaginal deliveries were reported during the EVD period. Secondly, facility delivery was more frequent and home delivery less frequent in the post-EVD period. Thirdly, walking and motorcycle transport remained common, while ambulance use was limited. These temporal patterns occurred during a period encompassing both the EVD outbreak and several health-system reforms. Because this was a retrospective cross-sectional study without a contemporaneous control group, the observed differences cannot be attributed causally to the outbreak, NEMS implementation, or other reforms.

### Maternal care before and during the EVD outbreak

The pre-EVD period was characterised by high proportions of home delivery and low C-section use, broadly consistent with the 2013 DHS findings.^15,27^ Previous studies reported substantial disruption of maternal services during the EVD outbreak, including reduced facility use, avoidance of care, and constrained availability of healthcare workers.^3,4,18,28,29^ In our data, however, home delivery declined across the three periods, while PHU delivery increased and hospital delivery was proportionally lower during the EVD period than before and after it. These findings suggest that changes in maternal service use were more complex than a uniform decline and may have involved redistribution of obstetric care from hospitals to PHUs.

Potential explanations include changes in care-seeking, service availability, infection-control concerns, redeployment or loss of healthcare workers, and initiatives intended to maintain essential maternal care services. These factors were not measured in the survey and should therefore be regarded as hypotheses rather than demonstrated explanations. The absence of reported AVD during the EVD period is notable, but without information about clinical indications or service availability, the study cannot determine why no AVDs were reported or whether any C-sections could have been avoided. Our results contribute to the literature by providing population-based evidence from Sierra Leone, highlighting the importance of considering both disruption and adaptation in understanding maternal health service utilisation during crises.

### Results in context

The observed increase in facility delivery is consistent with national survey findings: MICS-6 reported 79% facility delivery in 2017, and the 2019 DHS reported 83%.^30,14^ Despite this trend, ambulance use in our study remained below 2% in the post-EVD period, and walking remained common. By comparison, among a selected hospital cohort of women undergoing emergency C-section in 2016–2017, 36% reported ambulance transport and 6% reported walking only.^12^ Differences in study populations preclude direct comparison, but the contrasting figures demonstrate that transport patterns vary substantially by clinical and sampling context.

National C-section rates have steadily increased in Sierra Leone in the last decade, both well documented in household surveys and facility studies, rising from 1.9% in 2012, 3.4% in 2017, 5% in 2019 and finally 5.3% in 2023.^8,14,27,28^ Although the national C-section rate is well below recommended levels for optimal obstetric outcomes, the tertiary referral hospital reports C-section rates of close to 50% of all deliveries.^31^ The increase in national C-section rates occurred alongside higher facility-delivery rates, expansion of midwifery services, increased surgical care capacity through task-sharing, investments in surgical services, and implementation of NEMS. These concurrent developments may have contributed to the observed trend, but their individual effects cannot be determined from this study.^8,10,20^ It is of great concern that the ambulance service stopped functioning in 2022, possibly increasing second delays again. In contrast to C-section, AVD remains infrequently used in Sierra Leone. Previous studies identify equipment shortages and limited provider training as important barriers.^32–34^ Evidence from Uganda suggests that ensuring equipment availability, reinforcing clinical protocols, and providing repeated workplace training can increase appropriate use of vacuum extraction.^35^

In under-resourced settings, efforts to ensure appropriate use of C-section could include AVD training, perinatal audit, adherence to second-stage labour protocols, and monitoring using the Robson classification.^32,35,36,37,38^

The absence of substantial improvement in female education and literacy is particularly concerning. The ban on visibly pregnant girls attending school from 2010 to 2020 and “thus depriving girls of their right to education” may have contributed to this increase in female illiteracy rates in 2020 compared with 2012.^39^ A 2021 policy focusing on pregnant girls and parent learners is therefore relevant and hopeful, although its effects occurred after the survey and could not be evaluated in this study.^40^

### Strengths and limitations

This study provides a rare community-based perspective on obstetric care across a period of major health-system disruption and recovery. The nationwide sample was stratified by rural and urban residence, trained healthcare workers collected detailed lifetime obstetric histories, and digital data capture with real-time quality control supported data completeness. Comparison with SOSAS 2012 enabled exploration of changes over almost a decade and complemented DHS data, which do not report AVD or transport patterns. Due to methodological differences, comparisons between the two datasets are presented as descriptive and should be interpreted with caution.

The questionnaire was adapted from a validated survey instrument and pilot-tested but was not formally validated after modification, which may have affected the reliability of some measures. Comparisons with the SOSAS 2012 survey are subject to several limitations. Although both studies used cluster-based sampling approaches, differences in survey design, variable definitions, and categorisation limit direct comparability. For example, additional response categories for place of delivery were included in the 2020 survey but not in 2012.

Retrospective reporting of lifetime obstetric histories is susceptible to recall bias and misclassification, particularly for earlier pregnancies and outcomes such as AVD. The parent study was not powered specifically for obstetric outcomes, and the small numbers of C-sections and AVDs limit the precision of estimates. Each pregnancy in the lifetime obstetric histories reporting was analysed separately, and women could therefore contribute more than one pregnancy. Correlation between pregnancies reported by the same woman was not accounted for in the descriptive comparisons or chi-square tests.

Financial barriers to accessing emergency obstetric services were not directly measured.^5^ Maternal mortality data in this study were limited to deaths occurring in the year preceding the survey. These data were not validated against clinical records and could not be temporally assigned to the defined EVD periods. They are intended to provide descriptive contextual information. Finally, the data were collected in 2019–2020 and may not reflect current service use. These limitations should be considered when interpreting the findings and should guide future prospective monitoring.

## Conclusion

This community-based study identified temporal differences in obstetric care in Sierra Leone over a decade encompassing the EVD outbreak and major health system reforms. Facility births and skilled birth attendance increased markedly, C-section rates increased modestly, and assisted vaginal delivery, absent during the EVD outbreak, re-emerged. Transport barriers and minimal ambulance use highlight persistent delays in reaching facility-based delivery care. Girls’ education and literacy levels showed no substantial improvements over the decade. Continued investment in girls’ education, referral systems and multidisciplinary emergency obstetric training, including simulation-based training in assisted vaginal delivery, could accelerate progress towards safer motherhood in Sierra Leone.

## Supplemental material

Supplemental material - Caesarean section rates and obstetric practices in Sierra Leone before, during, and after the Ebola epidemic: Results from a nationwide household study Supplemental material for Caesarean section rates and obstetric practices in Sierra Leone before, during, and after the Ebola epidemic: Results from a nationwide household study by Josien Westendorp, , Hanna M. Mathéron, Alex J. van Duinen, Momoh L. Mansaray, Lora Daskalska, Osman Sankoh, Risa Hoffmann-Lonnée, Reinou S. Groen, Martin P. Grobusch and Håkon A. Bolkan in Women's Health.

Supplemental material - Caesarean section rates and obstetric practices in Sierra Leone before, during, and after the Ebola epidemic: Results from a nationwide household study Supplemental material for Caesarean section rates and obstetric practices in Sierra Leone before, during, and after the Ebola epidemic: Results from a nationwide household study by Josien Westendorp, , Hanna M. Mathéron, Alex J. van Duinen, Momoh L. Mansaray, Lora Daskalska, Osman Sankoh, Risa Hoffmann-Lonnée, Reinou S. Groen, Martin P. Grobusch and Håkon A. Bolkan in Women's Health.

Supplemental material - Caesarean section rates and obstetric practices in Sierra Leone before, during, and after the Ebola epidemic: Results from a nationwide household study Supplemental material for Caesarean section rates and obstetric practices in Sierra Leone before, during, and after the Ebola epidemic: Results from a nationwide household study by Josien Westendorp, , Hanna M. Mathéron, Alex J. van Duinen, Momoh L. Mansaray, Lora Daskalska, Osman Sankoh, Risa Hoffmann-Lonnée, Reinou S. Groen, Martin P. Grobusch and Håkon A. Bolkan in Women's Health.

## Data Availability

All data can be shared upon reasonable request.*
