# Supplementary material for: Caesarean section rates and obstetric practices in Sierra Leone before, during, and after the Ebola epidemic: Results from a nationwide household study
Source: Womens Health (Lond). 2026 Sep 21;22:17455057261487141. doi: 10.1177/17455057261487141 (PMC13731882; doi:10.1177/17455057261487141)
Supplement: Supplementary file 1 [file sj-pdf-1-whe-10.1177_17455057261487141.pdf]

Supplementary material – STROBE checklist

STROBE Item	Recommendation	Manuscript page	Notes
Title & Abstract	Cross-sectional design stated	p.1	Design stated in title and abstract
Background/Rationale	Scientific background and context	p.2–3	
Objectives	Specific objectives stated	p.3	
Study design	Key elements of design	p.4	
Setting	Setting and timeline	p.4	National data collection Oct–Nov 2019, Feb–Mar 2020
Participants	Eligibility and recruitment	p.4–5	Women 12–50 years, cluster sampling
Variables	Outcomes, exposures, confounders	p.5	AVD, C- section, transport, Ebola period defined
Data sources/measurement	Data collection methods	p.4–5	REDCap, trained surveyors
Bias	Efforts to address bias	p.13	Recall bias discussed
Study size	Sample size and rationale	p.4	914 women in 1,854 households
Quantitative variables	Variable definitions	p.5	
Statistical methods	Methods, software	p.5	Descriptive statistics, chi-square, Stata 15.1
Participants/results	Numbers at each stage	p.6	3,618 individuals, 914 women
Descriptive data	Participant characteristics	p.6–7	Table 1

Outcome data	Main outcomes	p.7–10	Tables 2–4
Main results	Key findings & estimates	p.8–10	
Other analyses	Additional analyses	p.7 & p.11	Comparison with SOSAS 2012
Key results	Summary of results	p.11	
Limitations	Study limitations	p.13	
Interpretation	Meaning and context	p.11–13	
Generalisability	External validity	p.13	
Funding	Funding sources	p.18	
Ethics	Ethical approval	p.15 & p.18	
