# Supplementary material for: Caesarean section rates and obstetric practices in Sierra Leone before, during, and after the Ebola epidemic: Results from a nationwide household study
Source: Womens Health (Lond). 2026 Sep 21;22:17455057261487141. doi: 10.1177/17455057261487141 (PMC13731882; doi:10.1177/17455057261487141)
Supplement: Supplementary file 2 [file sj-pdf-2-whe-10.1177_17455057261487141.pdf]

Supplementary appendix

A manual for the enumerators of the PREvalence Study on Surgical COnditions (PRESSCO) 2020

This appendix formed part of the original PRESSCO 2020 submission. With permission, the PRESSCO 2020 supplementary material is based and modelled after the supplementary material provided by: Groen RS, Samai M, Stewart K-A, *et al.* Untreated surgical conditions in Sierra Leone: a cluster randomised, cross-sectional, countrywide survey. Lancet 2012; published online Aug 15. http://dx.doi.org/10.1016/S0140-6736(12)61081-2.

Table of content

I. Summary Study Protocol 4

- Study Overview 4
  - PRESSCO 2020
  - Health topic I: Lower Urinary Tract Symptoms
  - Health topic II: Groin Hernias
  - Health topic III: Wounds
  - Health topic IV: Women's Health
  - Health topic V: Surgical Volume
- Methods 6
  - Study design
  - Sample size calculation
  - Data collection 8
  - Data Management and collection tool 11
- Ethical considerations 11
- References 12

II. Detailed Interviewers Manual (Adapted SOSAS manual) 13

III. Data checking protocol 29

IV. Data Management protocol 31

V. Tablet Manual 33

VI. Referral Letter 39

VII. Patient information sheet 41

VIII. List of abbreviations 43

IX. Definitions 44

Introduction to the enumerator manual

This manual was written to support the enumerators of the PREvalence Study on Surgical Conditions (PRESSCO) 2020. It also serves as a foundation for future researchers, and collaboratives, who wish to use our adapted Surgeons OverSeas Assessment of Surgical Need (SOSAS) tool for research purposes. In this supplementary material we successively describe a summary of the research project, explain the structure of the survey, elaborate on data collection & safety, discuss the use of the survey-tablets and have shared all information available to the members of the interviewed households.

During the four-day thorough training, followed by an assessment and selection day, all the manual documents were reviewed together with the selected enumerators of PRESSCO 2020. An explanation was given about the research design, the rationale of the research, administering the questionnaire and the correct management and transfer of the data. In accordance with the principles of the SOSAS group, we consider this PRESSCO 2020 manual a living document to which changes can be made in the future to improve research on 'surgical need' in low-and-lower-middle-income countries (LLMICs).

On behalf of the entire PRESSCO 2020 working group, and especially the principal investigators: Osman Sankoh, Martin P. Grobusch, and Hakon A. Bolkan.

Kind regards,

Jurre van Kesteren, MD
Lead for the overarching PRESSCO 2020 publication on:
Surgical Conditions in post-Ebola Outbreak Sierra Leone

Summary Study Protocol

The purpose of this study was to assess the prevalence of physical conditions needing surgical attention and how it changed over the past decade in Sierra Leone. The type of reported surgical conditions and associated anatomical location, health seeking behavior, and reasons for not receiving surgical care were investigated.

The study has one overarching theme and five additional health topics. Findings from each health topic will be reported in detail separately:

PRESSCO 2020: Repeat the Surgeon Over Seas Surgical Assessment Survey (SOSAS) from Sierra Leone 2012.[1] SOSAS has been performed in many other low- and lower middle-income countries (LLMICs) but never before conducted twice in the same country. PRESSCO 2020 is the first surgical household survey which can compare its data to an earlier study.

Additional health topics modeled on the SOSAS survey.

1) **LUTS:** Establish the prevalence of lower urinary tract symptoms (LUTS) among men 12 years or older, including an evaluation of health seeking behavior.
2) **Groin hernia:** Establish the prevalence and incidence of groin hernia and health seeking behavior.
3) **Wound:** Establish the prevalence of wounds in Sierra Leone and health-seeking behavior.
4) **Women's Health:** Establish the need for surgical female and obstetrical care in Sierra Leone, describe maternal and neonatal outcomes and their development in the pre-, during and post-Ebola time frame.
5) **Surgical volume:** Establish the rates of surgical procedures performed per year and determine contributions of the public and private sector.

Study Overview

PRESSCO 2020

SOSAS was performed in Sierra Leone in 2012, and subsequently in many other LLMICS revealing a large unmet need for surgical consultations.[1–5] Since 2012, Sierra Leone has experienced shocks to its health system, most notably the 2013 - 2016 West African Ebola Virus Disease (EVD) outbreak during which health care provision was disrupted. Up to 7% of all healthcare workers lost their lives in Sierra Leone[1]. The EVD epidemic severely impacted the provision of healthcare services and caused setbacks in the health seeking behavior, treatment and control of surgical conditions.[6–9]

To assess the prevalence of physical conditions needing surgical attention and how it changed over the past decade in Sierra Leone. This overarching article described the prevalence of physical conditions needing surgical attention, health seeking behavior, care received and reasons for not receiving surgical care. Findings from the other health topics will be reported in detail separately.

Health topic I: Lower Urinary Tract Symptoms (LUTS)

A potential implication of more knowledge of LUTS in Sierra Leone is the possible introduction of transurethral ethanol ablation of the prostate (TEAP). This low-cost therapy has been developed as a minimally invasive procedure for the treatment of patients with symptomatic benign prostatic hyperplasia (BPH).[10] Dehydrated ethanol is injected directly into the prostate, via the transurethral

route. Dehydrated alcohol (ethanol) is inexpensive and easily accessible which makes TEAP a good alternative for treatment of severe LUTS in low-resource countries. Depending on the outcomes of this study, it might be relevant to further investigate the feasibility of offering TEAP in Sierra Leone.

The main objective is to determine the prevalence of severe LUTS among men 12 years or older in Sierra Leone. This study will help to quantify how LUTS affects Quality of Life among the study population. To understand barriers towards health seeking behavior for LUTS. To estimate the need for treatment of LUTS in Sierra Leone.

Health topic II: Groin Hernias

In the SOSAS 2015 study from Nepal, a groin exam was not done, and that may be a reason for the lower-than-expected reported prevalence of inguinal hernias.[5]

To determine the prevalence of groin hernia among children, women and men in Sierra Leone. The prevalence will be determined based on a combination of responses to the questionnaire and a physical examination. A physical examination of the groin area will be performed if household members mention to have a solid/firm and or soft/reducible mass in that area. Furthermore, we will investigate the health seeking behavior of patients with groin hernias and estimate the recurrence rates.

Health topic III: Wounds

A significant proportion of severe wounds, burns, injuries, amputations, contractures and deaths may be preventable by improving knowledge on the perception and usage of modern and traditional medicine for wounds.

To describe the current prevalence of wounds and the status of wound care in Sierra Leone. To describe the prevalence of wounds in Sierra Leone. To study the etiology of wounds (traumatic, infectious, snake bites, burns, vascular). To describe the health seeking behavior of patients with wounds. To describe the surgical need in treatment of wounds. To describe the impact of wounds on daily life. To assess the level of hypertension in Sierra Leone, together with health seeking behavior for the hypertension.

Health topic IV: Women’s Health

This health topic will investigate the general status of women’s health and more specific need for surgical care and family planning methods used in women in their reproductive years at the moment of the interview. This information may help to understand the consequences for female and maternal health during a large EVD epidemic and it will assist us in appropriate allocation planning in the post EVD period.

It is the aim to provide a status update on the access and need for female and obstetrical care for women and mothers in Sierra Leone from a community perspective in the pre, during and post EVD time frame. A description on the amount and outcomes of pregnancies of women surveyed (12-50 years old) will be provided. For all deliveries we describe the actual and preferred place of delivery. Survey the availability of transportation (including the new ambulance transportation system) in the pre- during and post Ebola time frame. The findings will be compared with the findings of the maternal health section of the SOSAS study in 2012. [11]

Health topic V: Surgical volume
The main aim of the study is to determine the annual volume of surgical procedures conducted in Sierra Leone from a population-based perspective. Surgical procedures will be classified using the DCP3 essential surgical package. The contributions of the public and private surgical sectors will be assessed. In addition, 7- and 30 days perioperative mortality will be measured.

Methods

Study design

This is a population-based, cross-sectional household survey of surgical, urological and maternal health conditions. Participants will be recruited nationwide. Besides the overarching PRESSCO study, five topics will be investigated more in-depth to complement the previously designed SOSAS survey. PRESSCO 2020 survey consists of two sections. The first part is established to identify the number of household members, gather baseline data and quantify the number of surgical procedures among all household members. All data is gathered from the household head. In the second part of the survey two additional household members are randomly selected to undergo a head-to-toe verbal and physical examination if required.

Sample size calculation

The sample size for the first part of the survey is based on **Health topic V: Surgical Volume.** The selection of the household members will not be done randomly in this study arm, but all the household members of the randomly selected households will take part and serve as the denominator. All household members who have undergone a surgical procedure in the previous year will be included in the study.

Based on the found surgical met need by Bolkan *et al.,* the expected period prevalence of major surgical procedures is 0.4% in Sierra Leone.[12] Approximately 400 major surgeries per 100,000 population per year were performed in Sierra Leone. For the Surgical Volume we expect that at least twice as many minor procedures are performed. This results in an estimated prevalence (P) of major and minor surgical procedures combined of ± 1% per year. The level of precision is suggested within the range of 20% of the estimated prevalence, which allow us to apply an expected range (L) of (20% x 1% =) 0.002%.

$n = Z^2 p (1-p) / L^2$

n = sample size

Z = Confidence Interval (95% - Z is 1.96)

p = Estimated proportion of the prevalence of the condition looked for

L = Range excepted

$n= (1.96)^2 \times 0.01 \times (1 – 0.01) / (0.002)^2 = 9702$

Under the assumption that some household representatives will not consent to partake in the survey to elucidate on the surgical conditions of the other household members, we have chosen to add a margin

of 5% to the sample size. Hereby we will be able to estimate the population prevalence with a reasonable precision. For the first part of the household survey, we need to include 10,187 individuals

The sample size for the second section of the survey is based on the original SOSAS study protocol. A randomly selection of 1873 households will provide sufficient power to all of the five Health topics of PRESSCO 2020. We applied the original estimated prevalence of 7.3% surgical morbidity as used by Groen *et al*[10]. The estimated prevalence for LUTS, severe wounds and maternal and perinatal health are ±6%, ±1.9% and ±2% respectively[7,8].

The estimated proportion of the prevalence (p) of a surgical condition was set at 7.3%. The accepted range (L) around the estimated prevalence of the disorder is set at 1%. Letter Z is CI (95%–Z is 1·96). In Groen *et al.*'s pilot study from August 2011, 95% of the targeted population was eligible, and the same proportion responded.[13] This provides a response rate (95%) and eligible rate (95%) needed for the sample size correction. The sample size is further corrected for the population size and is multiplied by a design-effect (DEFF) of 1.3, assuming that surgical conditions are not very clustered. The estimated population of Sierra Leone was 7,09 million in in 2015.

$n = Z^2 p (1-p) / L^2$

n = sample size

Z = Confidence Interval (95% - Z is 1.96)

p = Estimated proportion of the prevalence of the condition looked for

L = Range excepted

$n = (1.96)^2 \times 0.073 \times (1 – 0.073) / (0.01)^2 = 2599.6$

Corrected sample = sample size x effect of population size Sierra Leone x DEFF x (1/response rate) x (1/eligible rate)

n x (1 + (n-1)/ 7,090,000) x DEFF x (1/response rate) x (1/eligible rate)

2599.6 x 1.00 x 1.3 x 1/0.95 x 1/0.95 = 3744.6 = 3745

For this second section of the household study, we aim to include 3,745 individuals. 1,873 household visits will be needed as we are including two randomly selected individuals per household

For the first part of the household survey, we need to include 10,187 individuals. The average household consists of 5.5 members; resulting in 1,852 household visits. For the second part of the survey, we need to include 3,745 individuals resulting in 1,873 household visits. By maximizing the household visits at 1,873 it should be sufficient for the first and second part of the survey.

Sampling method for the clusters

Sierra Leone is divided in four provinces, 16 districts, 190 chiefdoms, and 9,671 enumeration areas (EA), the smallest administrative units in Sierra Leone. Sampling will be done through a weighted random cluster design, where the probability of cluster choice is proportional to the population size. The clusters will be randomly chosen in a two-stage sampling process starting with the chiefdoms and

selecting the number of clusters needed out of the chiefdoms proportional to the population size in the chiefdoms to further select, the EA's out of the chiefdoms. Sampling is done by Statistics Sierra Leone, who will also provide maps and coordinates for the assigned clusters.

Assignment of the households within the cluster
If maps of structures of the randomly selected EAs are available, those are used for randomly designing the first household to approach. Thereafter every fifth structure at the right side of the interviewed household while standing with the back to the front door is approached for the survey. In case of more households per structure an on-site listing is made and random assignment of the household is facilitated by the use of the random calculator (available on tablet computer). To be able to have a weighted cluster sample, each cluster should have the same household numbers interviewed. Therefore, if a structure randomly chosen appears to be empty or the household does not give informed consent the next structure will be approached to be interviewed. Records will be kept on households' and individuals' refusal to participate, for the analysis of the results.

Sampling of the individuals for the five health topics
There are five different health topics. Three topics have a similar selection model and three topics only include household members under certain conditions. Inclusion of the household individuals will be according to the 'rolling the dice once rule'. Sampling of the two individuals starts after the determination of the household size. A random number calculator will assign two household members to be surveyed. If one of the selected household members is under the age of 12 years, that person needs a chaperone to assist. This can be any of the present and available household members over the age of 18 years. The two selected individuals receive the PRESCO 2020 survey including additional questions on groin hernia and wounds. If a randomly selected male is > 12 years of age, he will receive the additional LUTS questionnaire. If the selected household members are no men or men < 12 years of age no additional LUTS questionnaire will be used. If a randomly selected individual is a female >12 years <50 years she will receive the additional Women's Health questionnaire. Additionally, the household representative will receive questions about all of the household members that had a surgical operation in the last year.

If a person is selected but not available for the interview, an appointment is made for later that day or the following day (each cluster has a minimum of two interview days). If by the third appointment the person is still not available, this person will be excluded from the analysis. When the (randomly assigned) household member does not provide consent no replacement is sought. If neither household member is available, or do not give consent, the next household will be approached to maintain the weighted sample size of households per cluster (25 households in each cluster).

Data collection

Data will be collected via individual face-to-face interviews. National staff (students/nurses/medical staff and staff from Statistics Sierra Leone (Stats SL) will be recruited and trained to become enumerators. Enumerators will collect data with handheld tablets provided by PRESSCO 2020 research team. The recruited drivers will use a Global Positioning System to get the enumerators to the exact location.

The first part section of the survey is administered to a household representative to establish the number of household members, identify deaths in the household during the previous year, and establish whether any of the deceased household members had any of the following conditions in the week before their death: abdominal distension or pain; bleeding or illness during childbirth; injury; mass, growth, or swelling; acquired deformity; or a wound not due to injury or congenital deformity.

The second section consists of structured interviews of two randomly selected household members who undergo a head-to-toe verbal examination covering six anatomical regions: face, head, and neck; chest and breast; abdomen; groin, genitals, and buttocks; back; and arms and hands and legs and feet. The need for surgical care was recorded on the basis of an individual's response to whether they had a wound, burn, mass, deformity, or other condition needing surgical assessment or care—i.e., the respondent decided whether or not they felt they needed surgical care. A surgical procedure was defined as: wound care, suturing, incision, excision, or other manipulation of tissue, in a safe and painless way. Procedures were deemed major if they required regional or general anesthesia and minor if they required local anesthetics or none.

Additional Health topic specific data collection

Health topic I: LUTS

The randomly selected man or men older than 12 years of age will be asked for consent to participate in the LUTS arm. In addition to the SOSAS repeat, groin hernia and wound questions, they will be interviewed about the presence of lower urinary tract symptoms. The severity of LUTS symptoms will be assessed using the visual prostate symptom score (VPSS) visual questionnaire. VPSS is the visual analogue to the more commonly used international prostate symptom score (IPSS) questionnaire. VPSS evaluates patient symptoms with four pictograms; measuring pollakiuria, nocturia, urinary flow rate and life quality.

Uflowmeter

VPSS scores are evaluated in three categories, mild, moderate and severe. Only participants with a VPSS score >17, indicating a severe LUTS will be asked to complete a questionnaire related to health seeking behaviour. Also for study participants with VPSS score >17 we aim to measure flow of urine using a portable Uroflow (Uflow) meter. An Uflow meter can measure the peak urine flow rate and detect a weak urinary flow. The peak flow rate can be used as an objective measurement for detection of LUTS. The Uflow meter is a reusable plastic shaped funnel consisting of a cup and a spout divided into three chambers. The spout has a 4.6 mm diameter aperture placed at the bottom. When fluid is poured into the cup it will start to fill the funnel as well as flowing out through the aperture. At the time inflow and outflow are equal, it will maintain a constant maximal fluid level within the funnel.

Under assumption that the prevalence of severe LUTS is 2 %, the average household size is 5,5 persons and 65 % of the population above 12 years we estimate that from 1873 households approximately 67 participants will be asked to use the Uflow meter and complete the questionnaire about health seeking behavior.

Health topic II: Groin Hernia

When survey answers raise suspicion of a groin hernia (hard or soft mass), the enumerator will request consent to perform a physical examination of the groin area. The enumerator performing the physical examination for the hernia arm is trained to diagnose this medical condition. The enumerator is

experienced in differentiating between the types of groin hernias and other conditions in the groin and scrotal area; e.g. inguinal hernia, femoral hernia, scrotal hernia, lipoma, lymphadenopathy and hydrocele. He/she is able to differentiate between a direct and indirect hernia, based on physical examination and he/she understands and recognizes the differences between a reducible, non-reducible and strangulated hernia. The enumerator will inspect and palpate the groin area of the participant. Privacy of the participant will be kept in mind and respected by performing the physical examination in a shielded place. Physical examination will be done in a standardized way.

Healt topic III: Wounds

The two household members that are randomly selected will be included in the wound arm after having given consent. The wound survey will continue if the participant has a wound at the time of interview. The wound may be of any etiology, be at least 5 cm in length and/or at least one month old. A minimal limit size was chosen to prevent including participants with minor cuts. To not exclude patients with small but deep wounds, such as those caused by osteomyelitis, the additional inclusion criteria of a wound existing for at least one month was added. Additional consent will be asked to take four photographs: one close up of the wound, one of the affected area/limbs, one of the whole affected and contra-lateral limb or part of the body and one 3D-photo. All photos will be done in the same manner; outside, in front of a clear background, ruler next to the wound and number on a small sheet of paper in close proximity to the wound. The number on a small sheet of paper is part of a coding system whereby the photo can be traced back to the individual for the health analysis. The enumerator has disposable paper rulers, a new one is used for every examination.

The photographs will be taken by password protected Samsung tablets. The pictures will be shot in a way that the individual cannot be traced back; excluding identifiable body parts and/or tattoos. If inevitable at the time of the photo-shooting, during post-processing, the photo will be anonymized. The photo will be uploaded in the REDCAP software. Where possible, a 3D-photo or short video will be added to the three photographs. Photographs will be transferred and deleted from the tablets to a password-protected computer and password protected and encrypted cloud-based system.

Health topic IV: Women's Health

Every female who is among the two randomly selected household members and who is older than 12 years of age and younger than 50 years of age will be included in the Women's Health arm after consent. The selected household member(s) will undergo a verbal interview. For all women that are currently pregnant, the fundal height will be measured with a tape measure.

Health topic V: Surgical Volume

The household representative is solely responsible for this part of the questionnaire. The household representative will be asked to provide an overview of the household members that have undergone a surgical procedure in the past year. This accounts for both major and minor procedures. Through this interview, also all household members who passed away after surgery will be identified.

When the household representative identifies a member of the household who underwent a major surgical procedure within the last year, the survey will be extended with additional questions to extracting in depth information concerning the procedure. These questions focus on the type of the surgical problem and the (type of) facility where the surgical procedure was performed.

Data management and collection tool

Password secured Samsung tablets with mobile Internet access will be used to collect the data. The software used for the data collection is REDCAP (Research Electronic Data Capture). In case the mobile Internet connection is disrupted, the mobile application of REDCAP is able to store the collected data on the tablet. Once the Internet connection is restored, the collected data will be sent to the servers. Databases, web server hosting and technical assistance will be provided by University Medical Center Utrecht, The Netherlands.

All enumerators will only have role-based access to REDCAP to avoid unauthorized data access. Prior to the start of the data collection, all team members will receive training on the use of the application. REDCAP software application employs various methods to prevent security vulnerabilities. The anonymized data will be made accessible for secondary analysis after the closure of the study.

Four research teams will perform the data collection. Each team consists of: one driver, one supervisor, three interviewers and one international junior research assistant. This research assistant will provide backup and coordinate the logistical management. The enumerators will be either local nurses, medical students and or employees of Statistics Sierra Leone. If needed translator is needed one will be recruited on a per day basis.

Ethical considerations

Ethical clearance will be requested from the Masanga Medical Research Unit's Scientific Review Committee, the Norwegian Regional Committee for Medical and Health Research Ethics, and the Sierra Leone Ethics and Scientific Review Committee. Introduction letters from the Ministry of Health and Sanitation will be presented to the village chiefs and/or local administrators before the start of the study. All District Medical Officers will be informed about the survey and the visit of enumerators to their district.

If acute surgical conditions are identified during the survey, the participant or guardian will be informed about possible treatment options. Depending on the urgency, a letter of referral is written and/or referral to the local health facility is arranged. This is the task of the field coordinator. Enumerators can contact their field coordinator in case they meet a very ill person. The field coordinator will arrange transport if immediate referral is needed.

Early termination of the interview on behalf of the interviewed individual will be respected at all times. Privacy and confidentiality of results means that the answers the respondent gives are not shared with anybody. A responder's privacy should be honored at all times and information concerning the (surgical) condition should not be shared with anyone except persons designated by the responder. This means that the interview needs to take place in privacy. None of the published data can be traced back to one person or one location.

At the end of the interview, participants are given a business card with both contact information of the Medical Research Unit's Scientific Review Committee and the Ethical Committee of Sierra Leone for remaining questions or doubts.

References

1. Groen RS, Samai M, Stewart KA, et al. Untreated surgical conditions in Sierra Leone: A cluster randomised, cross-sectional, countrywide survey. *The Lancet*. 2012;380(9847):1082-1087.

2. Petroze RT, Groen RS, Niyonkuru F, et al. Estimating operative disease prevalence in a low-income country: Results of a nationwide population survey in Rwanda. *Surgery*. 2013;153(4):457-464.

3. Varela C, Young S, Groen R, Banza L, Mkandawire NC, Viste A. Untreated surgical conditions in Malawi: A randomised cross-sectional nationwide household survey. *Malawi Medical Journal*. 2017;29(3):231-236.

4. Tran TM, Fuller AT, Butler EK, et al. Burden of Surgical Conditions in Uganda. *Annals of Surgery*. 2017;266(2):389-399.

5. Gupta S, Shrestha S, Ranjit A, et al. Conditions, preventable deaths, procedures and validation of a countrywide survey of surgical care in Nepal. *British Journal of Surgery*. 2015;102(6):700-707.

6. Parpia AS, Ndeffo-Mbah ML, Wenzel NS, Galvani AP. Effects of response to 2014-2015 ebola outbreak on deaths from malaria, HIV/AIDS, and tuberculosis, West Africa. *Emerging Infectious Diseases*. 2016;22(3):433-441.

7. Brolin Ribacke KJ, van Duinen AJ, Nordenstedt H, et al. The impact of the West Africa Ebola outbreak on obstetric health care in Sierra Leone. *PLoS ONE*. 2016;11(2).

8. Bolkan HA, van Duinen A, Samai M, et al. Admissions and surgery as indicators of hospital functions in Sierra Leone during the west-African Ebola outbreak. *BMC Health Services Research*. 2018;18(1).

9. Huizenga E, van der Ende J, Zwinkels N, et al. A modified case definition to facilitate essential hospital care during Ebola outbreaks. Clin Infect Dis. 2019;68(10):1763-1768.

10. Goya N, Ishikawa N, Ito F, et al. Ethanol injection therapy of the prostate for benign prostatic hyperplasia: preliminary report on application of a new technique. Vol 162.; 1999.

11. Groen RS, Solomon J, Samai M, et al. Female health and family planning in Sierra Leone. *Obstetrics and Gynecology*. 2013;122(3):525-531.

12. Bolkan HA, von Schreeb J, Samai MM, et al. Met and unmet needs for surgery in Sierra Leone: A comprehensive, retrospective, countrywide survey from all health care facilities performing operations in 2012. *Surgery*. 2015;157(6):992-1001.

13. Groen RS, Samai M, Petroze RT, et al. Pilot Testing of a Population-based Surgical Survey Tool in Sierra Leone. *World Journal of Surgery*. 2012;36(4):771-774.

Detailed Interviewers Manual

The PREvalence Study on Surgical COnditions 2020 (PRESSCO 2020) was modeled after SOSAS, used the SOSAS survey tool and expanded with additional survey topics and physical examination. With permission of the original authors, the detailed interviewer's manual is **copied** and adjusted from the supplementary material of:

Groen RS, Samai M, Stewart K-A, et al. Untreated surgical conditions in Sierra Leone: a cluster randomised, cross-sectional, countrywide survey. Lancet 2012; published online Aug 15. http://dx.doi.org/10.1016/S0140-6736(12)61081-2.

The PRESSCO survey contains two parts and is further divided in four and ten sections for in depth interviews.

Section I (information from household head)
A Household Information;
B Living Household Members;
C Transportation Means;
D Deceased Household Members.

Section II (information from two randomly assigned household members)
E General Information;
F Face / Head / Neck;
G Breast / Chest;
H Back;
I Abdomen;
J Groin;
K Extremities.
L Women's Health;
M Wounds;
N Measurements.

In addition to SOSAS questions were added on groin hernias, lower urinary tract symptoms, women's health questions, wound analysis physical examination and blood pressure measurements at the end of the survey.

When entering a household, adhere to all known local customs. Building trust and collaboration begins by using appropriate greetings and friendly manners. Ask politely if the head of the household or the household member in charge is available for you; if he or she is not available, ask for another adult of the household. Directly identify yourself as an interviewer and show your PRESSCO ID card. Clearly explain why you are here and what you want (see patient information sheet).

After the interview with the household representative, two of the household members are randomly chosen with a random number calculator. Both household members are interviewed with sections E, F, G, H, I, J, and K. Section JL is for males above 12 years only, section L is for females only above 12 years only and section N is for adults only.

Proper identification of the household denominator; the number of members in the household (including their sex and age) is important. This process should be done securely with the household representative in order to determine randomly the two who need to be interviewed. A household member is any individual that is regularly eating from the same pot. If this definition is not giving a clear distinction you can add ‘and slept in the household the previous night before the interview’. This might be needed for males with more houses and wives. The male will be assigned to the household he slept in the night before the interview takes place.

For example, a niece who eats with the family and slept the night before the interview in the household is eligible. Additionally, a brother who is not available because he is searching for work elsewhere in the country for the last half year is not eligible. However, someone who is not available for the questionnaire due to work outside of the household during the day but sleeps in the household is eligible. This is very important to ensure random respondents.

For children under the age of 12, a parent or guardian should be asked to help answer the questions. If the child is assigned for interview but not available, it is permitted for the parent/guardian to act as surrogate. In other age groups this is not allowed. A person can always choose to have a guardian around during the interview to help, but the person him/herself should be available as well. In the age group between 12 and 18, the child should tell whether he/she wants a parent or guardian around for the interview or not.

Methods of Household Selection

In total 25 households need to be interviewed per cluster. The clusters are identified with the provided maps and are called Enumeration Area or EA.

The essence of the selection of the households is twofold:

1. The selection needs to be random, meaning that you should not choose the households based on their looks or otherwise.

2. The selection needs to be a good representation of the EA, it needs to be well spread over the entire area. If you would just concentrate on one part of the EA, you might end up interviewing all the households belonging to one family with their characteristics.

If an Enumeration Area (EA) has more than one locality or village, the enumerator needs to divide the total of 25 households over the different localities. Asking about the approximate number of households can make you divide the number per locality appropriately. If for example an EA has two

localities, and both have about the same size, you can interview 12 at one locality and 13 at the other locality. If, on the other hand one locality has only 25 households and the other 75 households you can do about 7 in the smaller locality and 18 in the bigger locality. The process described below will be taking place in both localities.

General Instructions of the Questionnaire

All questions must be answered unless otherwise indicated. Choose the multiple-choice option that is best fitting the description of the respondent's answer. If there is an option 'Other, specify_______', there should be new question where you are able to add in the specifics. Note that this only applicable for the ones where it says "specify", not the options where it says "other".

Only complete surveys can be analyzed and therefore it is most important that the survey is checked at the end of the interview. On the Tablet computer the date and your name are automatically saved by checking the box provided.

It is very important to have correct, complete information with this survey. The collected results should represent the reality. If the field coordinator notifies that your surveys are not complete, are done to fast, rushing through the questions, not waiting for appropriate responses from the respondents, you will be given a warning. If, after the warning, the quality of your interviewing is not getting better, you can be expelled from further interviewing.

It is important to let the interview be evolve as natural conversation as possible; it is basically a conversation about the health of the respondent, structured with the questions we put in this survey. The person needs to be comfortable with you to be able to express all the health problems we are interested in. Therefore, don't give the impression that it is a test, or that the person provides 'wrong' answers. Tell them to feel at ease and that it is not a problem for you to repeat a question or explain it better if the person doesn't understand what you are asking.

<u>Specific instructions</u>

- *[Items in italics are instructions to the interviewer, containing definitions, explanations or emphasis. You can use this information to explain the question better if the respondent does not understand you.]*

Paragraphs in the boxes are explanations during the interview for the respondent. Read these out loud and be sure that the person understands the explanation.

How to mark your answer?

- The questions in the paper format can be answered by using the code provided at the question.
  Example: Question L3.4.6. Mode of delivery. What was the mode of delivery?
  S = spontaneous vaginal delivery, V = assisted delivery (Vacuum or forceps), C = Caesarean Delivery / operation
- Or the question can be answered by filling in the blank
  Example: Question E6. Education
  _x_None
  ___Primary (etc)

- Or by checking the right box in the table:

Example: Question F2.1 Face / Head / Neck specifics:
Tell me what problem you have had.
Wound injury related
Wound not injury related
Burn
Mass or growth / goiter
Deformity congenital
Deformity acquired

Problem 1	Problem 2	Problem 3
x		
	x	

- If a question can be skipped this is indicated, see question B.5 Type of surgical procedure (QI) for an example.

PRESSCO 2020 Sections

Section A through D is information about the household provided by the household representative. All the questions from a section are numbered with a letter and a number of the question. Section A starts with Question A1.

Section A HOUSEHOLD INFORMATION

Question A1 asks the number of visits to that household. Sometimes you will have to leave a household and come back at a later time if, for example, the household representative is unavailable at the moment.
Question A2 ask which village/cluster the household is located.
Question A3 asks what type of village it is (rural, urban or slum).
Question A4 asks for the Household code.
Question A5: Here you should write your name or on the tablet your enumerator code.

Introduce yourself and explain the purpose of your visit and what will be happening. Deliver the information sheets and consent forms. Before going through to the next section, you need to get informed consent from the household representative. Without consent you cannot proceed. Make sure the participant understands the purpose of the study. If they do now want to participate, ask why and mark this.

Section B LIVING HOUSEHOLD MEMBERS

The household representative will give you the household denominator; how many people live in the household you are visiting. Remember well the definition of a household member: ‘the person who eats from the same pot and slept here last night.’ Age and sex are asked. Sometimes age must be provided with developmental milestones for children and event calendars for adults.

After collecting the information on the household denominator, you can go through the following steps to assign 2 household members for the questions later on in section E-N.

1. Check if all information is filled in for all the household members, remember the definition: all the persons eating from the same pot and sleeping in that house

2. Make sure that for each household member the age and the sex has been provided.
3. Check the order of the household members. This should be starting with the oldest person and ending with the youngest person, make sure you ask for neonates/babies.
4. Make corrections before you go to step 5.
5. Remember the total numbers of living household members. Open your Random Generator on your tablet: fill in for 'Min':1 and for 'Max': the number of living household members. Press Generate. The two numbers which appears corresponds with the numbers of the household members to interview later on section E-N.

After having randomly assigned the two household members for the questions in section E-N, continue with the part 'Household members after surgical procedure' starting with question B3. These questions will be only about the household members who underwent a surgical procedure in the last 12 months. If any of the household member underwent a major surgical procedure in the last 12 months, a new tab will be opened with extra questions for more details about each major surgical procedure the household member has undergone.

Section C TRANSPORTATION MEANS

Section C contains questions on the travel time and way of the household members to the different hospitals and healthcare facilities (primary, secondary and tertiary).
There are 5 questions to each healthcare level: What is their main transport way? Bus, car, on foot etc. How long does the transportation in total take without delays (waiting/traffic congestion)? How long do you normally have to wait? What does the transportation to the specific facility cost? Are you able to provide that transportation cost for a sick household member?

Definitions used:

Primary health facility:	*Health facility without (functioning) operating room*
Secondary health facility:	*Health facility with functioning operating room*
Tertiary health facility:	*Health facility with functioning operating room and minimal one surgical specialist (Surgeons/Orthopedics/Gynecologist/Urologist)*

During the training period the facility types per cluster will be discussed so that you are able to call the village/city where this type of facility can be found for the person to be able to respond. There is a list of the different tertiary hospitals, so it's easy to know which ones are secondary and tertiary.

Time guideline: one person can walk 3 miles in one hour or 1 mile takes 20 minutes to walk.

Section D DECEASED HOUSEHOLD MEMBERS

This section is also filled in with the household representative; the questions are to investigate the circumstances of deceased persons in that household during the last 12 months. If the household did not experience a household death in the last year (i.e., last 12 months) the questions are not asked. If more than one household member deceased in the last year you need to repeat the questions and fill in a new tab (one tab per deceased household member). Make sure you answer all the questions in D for one deceased household member before going to the next.
Questions about a deceased household member might trigger grief and other emotions. Stay calm, show empathy and let the person first tell their story, before going into detail about the answers you need for the survey.

The following questions are asked: How old was the household member when s/he died? What was the sex of the household member? If she was a female, was she pregnant? Emphasize that we would also
like to know if a baby or newborn died in the family.

Question D3 is asking if there were any signs of surgically treatable conditions in the last week before death. Ask all the options separately.

Question D4 is asking about healthcare seeking behavior of the deceased. Did the deceased household member look for healthcare? Yes / No followed by D4.1 did the deceased household member look for a traditional healer? It is important to ask these questions without judgment or presumptions. These questions are very similar as what you will find in the sections F-K.

Question D5 is a about the type of healthcare received for the problem causing the death. There will be a repetition of several questions about minor and major surgery as asked in section B. Again, if a deceased household member underwent major surgery in the last 12 months, a 'tab' for each major procedure will be opened for further specific questions. This will correspondent to question D8 up until D12.

Question D6 asks about the main reason that the household member did not receive surgical care. Only one answer is allowed, so choose the answers that covers the main reason for not having surgical care. This question will also be seen in a similar way in sections F-K.

Question D7 is about the location of death. At home/in the health facility or elsewhere?

Question D8 until D12 are, as explained above, a repetition of the questions about major surgery in the last 12 months as done in section B.

The last section is an open box where you need to give a short explanation of the circumstances during the death of that household member.

Continue with the next tab, if there was a second deceased household member in the last 12 months before the interview.

Section E through N is information about the two randomly selected individuals and therefore in the printed PRESSCO these sections need to be provided twice for each household. The copies of the paper format should therefore provide section E – N twice. Section JL is only applicable for males over 12 years, section L is only applicable for females above 12 years and section N is only applicable for adults (male or female, 18 years or older).

Section E GENERAL INFORMATION

Question E1. Enter the Household list number. This is the number provided in front of the household list, which you completed earlier with the representative of that household. You are allowed to choose another household member using the random calculator if the household member who was initially assigned is not available after the third visit to the household. If so, mark the box at the end of the answer space for question E1 which says that the person has now been replaced. You are not allowed to choose another household member if the ones who were initially chosen to participate refuse to give consent.

Section E starts after you give an introduction on the survey to the household member, and listed the participation number, his/her sex and age. It is very important to have full cooperation. In order to achieve this, you need to take time to explain the reason for this study. Allow him/her to ask questions if they do not fully understand. A person who fully understands the reason of the survey is more likely to cooperate and to give his/her consent.

Minors (individuals under the age of 18) need their parents or guardians (often the household representative) to consent for them, and they need to give consent themselves as well. (Questions E4 and E5) Only for children under the age of 12, the parent or guardian can respond without the present of the child. In that case you mark E5 as Surrogate consent only. If the child is around, you ask also the permission to participate (assent, question E5) and the interview will be held together with the guardian/parent. If the child is older than 12 the guardian/parent can be around for the interview depending on the wish of the child.

The following questions are about some general demographics; education, illiteracy, occupation and ethnicity and duration of the stay in that household. (Questions E6-E9)

Questions E10-E13 are questions we will not analyze in depth. Those questions are asked to help the person you interview to start thinking about their health. It is important for the person to be comfortable with you and the interview should be as natural as possible; just like a normal conversation about their health.

Questions E14- E19 are questions about different medical conditions and life habits that could be present in the (life of the) household member. Try to find the best possible answer. Even if, at first, someone is saying not to know the exact answer to the questions. Estimations of the amount of, for example, alcohol use, is better to 'rather not say'. By asking your questions in a respectful, non-judgmental way, people are more likely to give honest answers.

Questions in E20 are questions about mobility, self-care, activities, pain and mental status to measure generic health status in a standardized way. Choose only one answer, which is most applicable for this specific household member in this moment.
After question E20.6 an explanation about surgery follows. Read this for the respondent and explain the next step as is provided in the text box ('I'm now going to ask you about all the surgical problems you've had in your lifetime. We'll start with your head and move all the way down to your toes.')

SECTIONS F/G/H/I/J/K	ANATOMICAL LOCATIONS
Section F	FACE, HEAD OR NECK
Section G	CHEST AND BREAST
Section H	BACK
Section I	ABDOMEN
Section J	GROIN, GENITALIA AND BUTTOCKS
Section K	EXTREMITIES

IMPORTANT:
Each section starts with a general question about that anatomical location. If the answer to that question is 'No', the rest of the questions in that specific section can be skipped. However, be careful. The question cannot simply be read. You really need to investigate if the person had a problem with that anatomical location you are talking about. Therefore, start with:

1. Have you ever had a problem with your [face, head or neck]?
2. If 'No', You never had a wound or burn in your [face, head or neck]? Never had a mass, Problem with eating/drinking? Never had a problem with your eyes or ears? Never had an operation in your [face, head, neck]?
3. If all stays 'No', you mark that in the first question, and you continue with the following anatomical section.

1. Have you ever had a problem with your [face, head or neck]?
2. If 'Yes', tell me what happened?
3. Let the person tell their story while you check if that is one of the options given in the first question. If it is one of the options: mark 'yes' and continue with the following questions to further investigate that problem(how many times have you had this problem).
4. If the explanation does not give the impression to be a surgical treatable condition you continue by saying: 'I'm especially interested in whether you had a wound, burn, mass etc. (see above)' and mark the answer option appropriately.

The same way of asking the questions should be applied in the other sections (G-K)

If a respondent has more than one problem with an anatomical location you should mark them in a different tabs (Tab) / columns (paper interview), marked as problem 1, problem 2, problem 3. If there are more than 3 problems per anatomical section, mark the most recent and most severe problems.

Always start with problem 1. Next fill in problem 2 and as third, fill in problem 3.

Section F (Face, head neck) and Section K (Extremities) starts with a question to further specify the location of the problem.

Questions F/G/H/I/J/K-2 are always about what the problem is, categorized into surgical problems identifiable by the patient. Explanations and definitions are given as well next to the question.

Question F/G/H/I/J/K-2.1-2 is about the type of injury, and if it was due to an accident. In that case, what kind of accident. Answer options are provided.

Question F/G/H/I/J/K-3.1.1 is about the timing of the problem. When did it start? Question F/G/H/J/K-3.1.2 asks if it is still an issue. Do they still have the problem now or during the last week? This will give information on surgical problems that are chronic. Question F/G/H/I/J/K-3.2.1-2 is about the size of the burn / wound and how it started. Here you need to measure the wound with a new ruler. It should be measured on the largest diameter in millimeters. 10 mm = 1 cm. See explanations section

<u>Section M. WOUNDS</u>.

Question F/G/H/I/J/K-3.3 is about the initial treatment of burns. Explain that we want to know if they put anything on the wound directly after the burn. Answer options are provided, but if the answer is not there, fill it in under "other". You can present examples like "did you wash it or did you put aloe vera on it?" so that they understand what you are asking.

Question K-3.4 is simply to indicate a broken or dislocated joint. Answer options are 'Yes' or 'No'.

Questions F/G/H/I/J/K-4 are like question D-4 and is described above. It is about healthcare seeking behavior of the respondent. Yes/No, followed by what kind of healthcare.

Questions F/G/H/I/J/K-5 are like question D-5 and is described above. It is about what kind of treatment / healthcare they have received. Answer options are provided.

Questions F/G/H/I/J/K-6 are like question D-6 and is described above. It is about reasons for not having surgical care. If the person did not go to a health facility, did not receive any surgical care or went to a traditional healer, it's important to ask the respondent why. Answer options are provided.

Questions F/G/H/I/J/K-7 asks about disability. Does the problem the respondent presents impact his/her life? Or does this condition make it difficult to carry out daily activities. The respondent might not be familiar with disability as such and therefore you can read out load the answer options. Also, if the person feels ashamed due to the condition this should be marked. The answer options are gradual in order, chose the lowest answer applicable. Meaning someone can feel ashamed about his/her need in help for transportation, but you than only mark 'I need help with transportation'. Another example: if someone needs help with daily living as well as transportation you mark the lowest answer applicable 'I physical need help with daily living'.

<u>Section Jpe PHYSICAL EXAMINATION GROIN</u>

This section is an extension of section J. groin / genitalia / buttocks. If one of the identified problems in
section J. groin / genitalia / buttocks is possible a groin hernia (only if J2.1 is answered "mass (solid)", "mass (soft or reducible)" or "hernia"), follow the two next steps:

1. Ask the questions about the treatment of groin hernias. Questions J8.1.1-4 asks about the history of treatment of groin hernia and if the respondent is aware of the fact that it is an operable condition, if they are previously operated for it, how many times and at which facility the operation was done.
2. Call the field supervisor to let the participant be physical examined. The field supervisor will execute the physical examination. Together with to field supervisor you will fill in the results of the physical examination on the tablet.

Section JL LOWER URINARY TRACT SYMPTOMS

This section is to determine if the participant has any problems with urination. Section JL is only to ask men older than 12 years. This section is divided into two parts. In the first part we use a visual scale to detect the severity of the problem. The form consists of four questions with pictures for explanation.
JL1.1 How many times do you need to pee during daytime? (1-6)
JL1.2 How many times do you need to pee during the night? (1-6)
JL1.3 How does your peeing look like? (1-5)
JL1.4 How much does the peeing affect your life? (0-6)

The score adds up automatically on the tablet. If the score is 17 or higher, offer the participant bags of water and inform them that we want them to pee in a plastic tube so that we can measure the peeing. If they say that they can go to the bathroom and pee right away, try to make them drink a bag of water first anyway. This is so that we know they have a certain amount in their bladder. If they pee with just a small amount of urine in their bladder, this may affect the result of the flow-measurements. Ask the participant to let you know when they have to pee/feel that their bladder is full. Call the field supervisor for arranging the measurement. Only if the score is 17 and above you ask the following questions.

Question JL-2 asks about the timing of the problem. For how many months have you had this problem?
Question JL-3 - JL-7 are like question D4-D6 and is described above.
Question JL-8 are like question F/G/H/I/J/K-7 and is described above.
Question JL9.1-2 asks about penile discharge. Have you ever had penile discharge, and if yes have you ever been treated for penile discharge?

JL10 Uroflow meter will be used to measure the peeing. The field manager will arrange this.

Section L. WOMEN'S HEALTH

Because of the sensitivity of the questions, women above 12 years should be interviewed by female interviewers. Section L is the first section after section E until K that does not focus on a specific anatomical location. This section is only about women that are 12 years or older and as you can understand, these questions are about a private issue. So, it is important to ensure confidentiality of the information given by the woman. This section is divided into 4 different parts.

Part L 1. Eligibility for Reproductive life survey
Part L 2. Gynecologic Complaints survey & Family Planning survey
Part L 3. Reproductive life survey & Pregnancy/Obstetrical History
Part L 4. Perinatal death section (only if loss of a newborn in part L 3.)

The first part L 1. is to find out if the selected household member is applicable for this part of the questionnaire or not. If the answer to one of the questions in Part L.1. is ‘yes’, you can skip all the following parts in this section and go directly to Section M: Wounds.

The second part L 2. Is about menstrual period and family planning.

Question L2.1 until L2.6 are different questions about the household member’s menstrual period. Question L2.7 askes about possible problems regarding the menstrual period and health seeking behavior for these problems. Question L2.8 until L2.11 are questions about use of family planning, possibilities, type and duration of using it.

The third part L 3. of this section focuses on pregnancies. The questions will be about all pregnancies in the past and (if present) the current pregnancy. As some pregnancies might be long ago, it might be difficult for the household member to remember. Try to encourage the participant to answer the questions as detailed as possible. If they don’t know the details, tell the participant not to hesitate to say that they don’t know. Honesty is better than made-up answers.

Part L 3 start with the questions L3.1 until L3.2.7. about the current pregnancy (if present). In some cultures, the woman is not allowed to tell you that she is pregnant, until the abdomen shows her pregnancy. Try to get an honest response, but if the woman doesn’t want to tell, or doesn’t know whether she is pregnant, mark ‘I don’t know’. Question L 3.2.2, L 3.2.3. and L 3.2.3.1 could indicate medical problems that need either referral or transportation to a hospital. To know whether one of these two things is indicated, see the ‘referral and transport’-protocol. Always contact the Field Supervisor if you think one of these options should be considerate for the mentioned medical problem.

Questions L3.2.4 is about the place they are expected to deliver. This could be different from the place they wish to deliver, because of transportation, money or other issues. That is why question L3.2.5 is about the place they would desire to deliver in the ideal situation. Question L3.2. is to clarify who decides where the woman should go for her delivery.

Question L3.2.7: ask the woman if she agrees to the measure the fundal height. Ask her to lie down on her back. With tape measure, you measure the distance from her symphyses until the fundus of the uterus, over the midline (see picture). Write down the distance in centimeters.

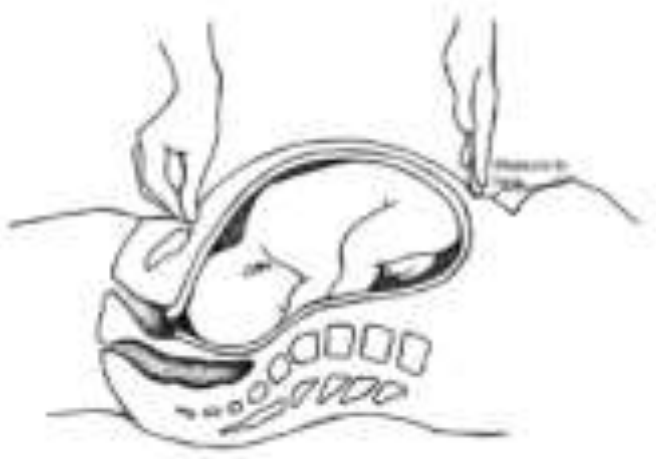

Questions L3.3.1 and L3.3.2 ask if the woman is currently breastfeeding.

With questions L3.4. until L3.5.2 an obstetrical history will be made of all the different pregnancies the participant has had in the past. This means that all questions L3.4.1 until L3.5.2. are asked for every single pregnancy and miscarriage in the past, in order of appearance, starting with the first pregnancy. The first question, L3.4 is very important. Take your time to get to the right number of pregnancies in a chronological order, as the other questions following after will make no sense if there are mistakes in this section.

If the answer to L3.4.3 (Outcome of the pregnancy) is S, D, or E, please check with the participant if she is willing to continue. Tell her that you are sorry to hear if she lost any babies and that you want to ask some more questions about her deceased baby(/babies) later if that is all right with her.

The fourth, and last part of this section L 4. is about perinatal death. This part is only applicable for participants that answered S, D or E to question L 3.4.3. If the answer to this question is <u>not</u> S, D or E, you can skip all the following questions in the remaining part of Section L and go to Section M, Wounds.
In the case that the answer to question L 3.4.3 is S, D or E, start with checking if she is willing to answer some questions about her baby that passed away. Explain again that she can tell you to stop whenever she wants. A respectful, patience and understanding attitude is very important and helpful in this sensitive situation. At the end of part 4, please give an explanation of the story told for every perinatal death if applicable.

<u>Section M. WOUNDS</u>

Explanation about taking photos of the wound:

1. Wear gloves
2. Put the leg/arm on a clean white plastic (apron).
3. Remove the bandage carefully if present.
4. Put a paper with the participant number next to the wound.
5. Measure the wound, take the largest diameter (see photo).
6. Take 3 photos
    a. Of the entire wound. (Get close to the wound, do NOT use zoom).
    b. Of the wound and the surrounding tissue (e.g., the whole lower leg or whole lover arm or whole chest or face or abdomen).
    c. Of the wound and the whole extremity + the other side (e.g., both legs or arms, whole chest and part of face and abdomen).

7. Attempt to not photograph the patient eyes to make sure that the patient is not recognizable afterwards. If the patient has wounds in the face or e.g., a very unique skin mark, then we will try afterwards to edit out any recognizable feature. You do not have to do this yourself.
8. Re-apply the dressing: use a clean gauze with povidone and a new bandage. If there was no dressing present, you do not have to dress it.
9. When in doubt what to advice a person with a large/severe wound, or if you believe the patient is very sick due to the wound: consult the SACHO present and tell them that they should go to a nearby hospital. They are always welcome at Masanga hospital for wound care.

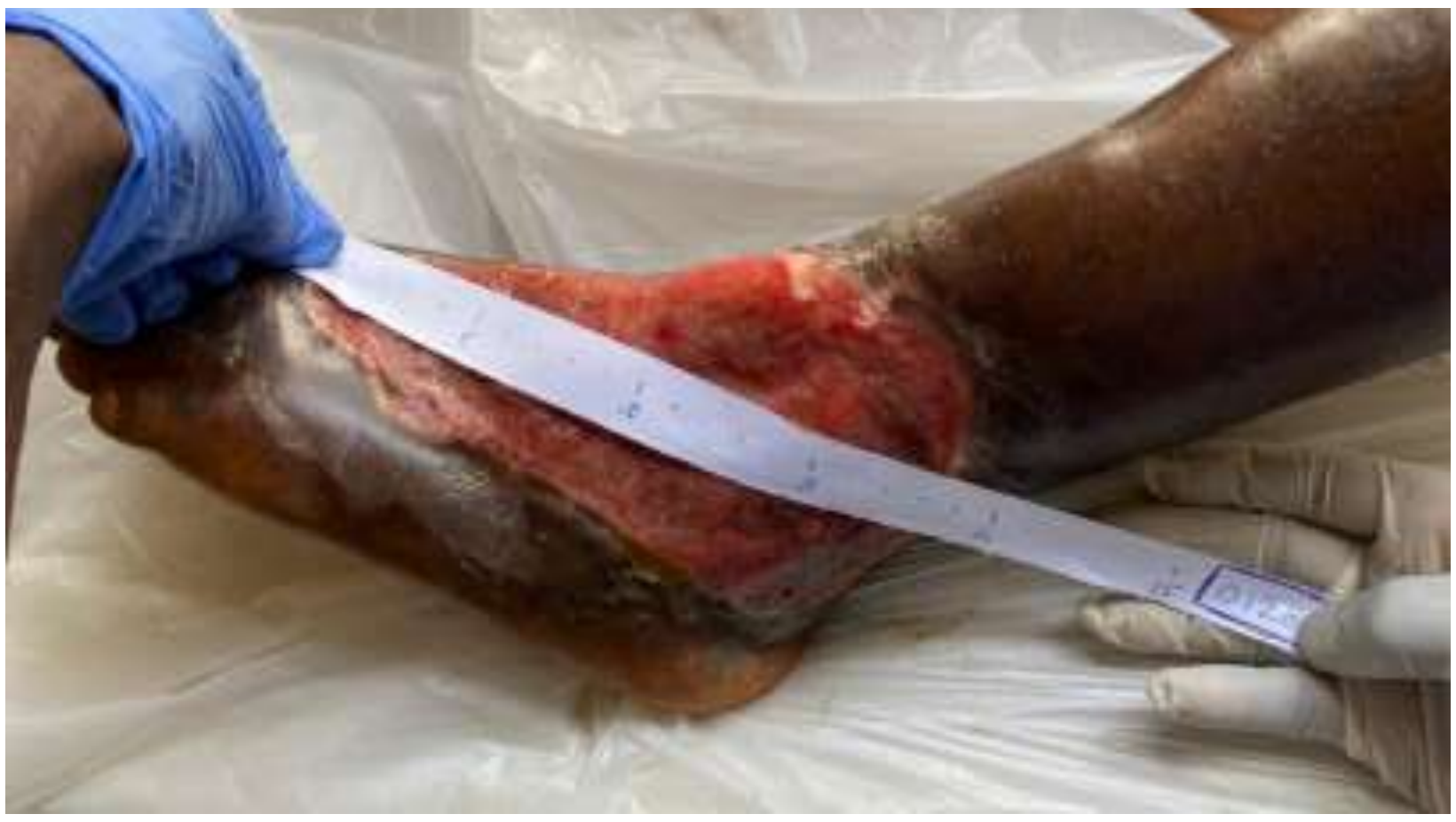

The questions in this section are only asked if there were any wounds present in the previous sections. Question M1 asks if the respondent have any wounds or burns at the moment, thereafter question M1.2 asks about how the amount of wounds and burns.

Question M2 is about the duration of the wound(s). If there are multiple wounds, choose the three that has been present the longest. Only wounds that are larger than 5 centimeters in diameter or wounds that have been present 1 month or longer will be further included.

Question M3 is about the size of the wound. The wound needs to be measured with a new ruler. Take the largest diameter. If there are several wounds, measure the three largest ones. The wound need to be bigger than 5 cm in diameter to be included. Please see the photo above.

Question M4. is to obtain consent for taking photos of the wound. Tell the respondent that it might be used for study purposes and publication in scientific journals and other media. If not done before, explain (again) that photographs will be anonymized as much as possible and are stored on a password protected system. If the participant does not want pictures taken, please ask(respectfully) why not and fill this in (no time/ no willingness /no reason / not seen benefit / other: explain). Only wounds larger than 5 cm (50mm) or wounds that have been present for one month or longer will be included

Question M5. is about possible exposure to the feet of the participant to stagnant water. This is question will be asked in all cases of a present wound, regardless of the location of the wound.

Question M6 and M7 are about pain. For question M.6. please show the 'Wong-Baker FACES Pain Rating Scale' to the participant, explain the meaning of each different face (0 = no hurt, 2 = hurts just a little, 4 = hurts a little bit more, 6 = hurts even more, 8 = hurts a whole lot, 10 = hurts as much as you can imagine, although you don't have to be crying to have this worst pain). Ask the participant to choose the face that best depicts the pain they are experiencing. If they have multiple wounds, choose the one that is most painful.
Question M7.1. is about the number of times per week the household member takes painkillers for the here above described pain. It is not about the number of tablets taken, but about the number of days that painkillers are used in a normal week.

Question M8 asks about how often the wound makes it impossible to work. This could be because of several reasons (pain, not allowed to work from boss, admitted to health care facility, too sick to work, unable to use extremities, fear wound will get worse). It's not important what the exact reason is, but how often this happens (1 = not at all, 2 = not often, 3 = sometimes, 4 = often, 5 = always).

End of the questionnaire

After this last question it is very important to check the entire survey. Go over all the tabs to ensure that you have everything. Ask the questions again which you accidentally skipped. You can add some comments about the interview at the very end. This can be used for questions about some of your responses or if you want to document a medical story a bit more in detail for verification of the categorization you made. Explain to the participant that the survey ends here and, if the household member is 18 years or older, that a colleague will come and take blood pressure measurements. S/he will measure the blood pressure and will explain more about it.

After checking there is no data missing and every question is answered, please sign the survey with date, name and signature. Remember to thank the participant for participating and ask if they have any questions before you leave. You can always ask the Field Supervisor if you don't know the answer to a question yourself. Tell the participant that they're always welcome to contact us on a later moment and wish them a nice rest of the day.

If this was the first randomly chosen household member, you can continue with the second respondent by starting Survey 2 from section E down again. If this was the second household member, let your research team/ Field Supervisor know.

Section N. MEASUREMENTS

Section N is only for participants that are 18 years and older.

Preparations: Always be respectful towards the participant. Make sure the participant feels comfortable. Unless the participant does not want to, take the measurements in a completely private place just like the rest of the interview. Kindly ask the participant to remove excess clothing and heavy accessories, such as coats, scarves, and heavy jewelry before the measurements. Make sure that the left arm is not tightly covered for measuring the blood pressure.

Tell the participant the following: "*Good morning/afternoon/evening, today I am assisting the research nurse that asked you all questions. I would like to measure your blood pressure and your heart rate. Your blood pressure can be healthy, too low or too high. If the blood pressure is too low or too high, doctors and health care workers can help you with establishing a healthy blood pressure. To measure your blood pressure, I will put this cuff around your arm and ask you to follow my instructions. I will take you blood pressure three times in total. Between two measurements we will wait for 2 to 3 minutes. Can I proceed or do you have any questions?*"

Measurement-instructions:

- The participant has to be seated with uncrossed legs and relaxed for 5 minutes before the first blood pressure reading is taken. In case the participant has been seated through the whole interview you can move to the first measurement without waiting those 5 minutes]
- Place the left arm of the participants slightly bent and with the palm facing upwards
- Put the blood pressure measurement instrument around the left wrist of the participant with the instrument on the medial side of the arm
- Assure that the blood pressure measurement instrument is tightly placed on the wrist
- Assure that the arm does not move and ask the person not to talk while taking the measurement
- Press the start button
- Wait until the blood pressure measurement instrument shows the three results
    - Dia
    - Sys
    - Rate
- Write down the results
- Switch off the blood pressure measurement instrument
- Wait for 2 to 3 minutes before taking the second measurement of blood pressure. Instruct the participant to keep seated during this period.
- Press the start button again
- Take the three numbers again and write down the results
- Switch off the blood pressure measurement instrument
- Wait for 2 to 3 minutes before taking the third measurement of blood pressure. Instruct the participant to keep seated during this period.
- Write down the results
- Call the field supervisor when the second and the third measurement of the blood pressure have either:
    - A diastolic (dia) level that is higher than 90 mmHg
    - A systolic (sys) level that is higher than 140 mmHg
    - If the person is pregnant and the BP is systolic > 140 or diast > 90-100
        - Inform the field coordinator and ask the woman about any PE complaints like headache, blurred vision, edema. If present: refer woman to a nearby hospital with a NEMS ambulance. If no complaints she should be advised to seek for medical care the same day at a CHC or hospital.
    - If the person is pregnant and the BP is syst >160 or diast >110 without any complaints, she needs to be referred immediately with a NEMS ambulance. Thank the participant for their cooperation and their time.

VII FINAL NOTES

As you might have noticed, this survey will not make diagnosis as such, since it does not include a physical exam. It gives insight in the prevalence of the need for surgical consultations, and most likely interventions. The quality of the interview is very dependent on how well you know the questions and definitions. Please mark all your questions and remarks below, check if you understand each question and verify everything during the training to make sure that everything is clear before going into the field to interview. In the field the Field Supervisor and the Expat can help you with questions based on situations. Your per diem allows you to use your phone during the interview and during the travel time you can come back on situations.

Kind regards, Masanga Medical Research Unit

SPACE FOR QUESTIONS AND NOTES

Data Checking Protocol

This protocol was used during the field work period of PRESSCO 2020 to ensure the quality of the data. Incoming data was checked on a daily base so that the research teams could continue to the next Enumerator Area (EA) once a EA was finished.

Main rules:

1. As long as there is internet reception, the team doesn't leave the EA until the external checkers give the OK.
2. If there is no internet reception, the team doesn't leave the EA until the data checkers have seen all records and given the OK.

In the field:

1. Interviewer takes interview
2. Interviewer checks own interview: are all questions answered completely and correctly? If there are any mistakes, correct them immediately.
3. Interviewer brings tablet to data checker
4. Data checker checks full interview: are all questions answered completely and correctly?
5. If any mistakes are found, interviewer goes back to household to repair mistakes.
6. When this has been done, data checker uploads record to the server. While record is uploading, interviewer goes to the next household to start the household list.
7. When record is uploaded, data checker sends message in the external checker WhatsApp group for that team, that records are available to be checked. The data checker will also make note of the time they are planning to leave the EA, so the external checker knows how urgent the checking is.
8. All steps are recorded on 'recording sheet' per EA for the team, see attachment.

Out of the field

1. The day will be divided into 2 shifts: 8.30-12.00 and 13.00 to 17.00 (SL time, EU time is 9.30-13.00 and 14.00 to 18.00). One person will be assigned to each time slot.
2. As the message comes in that data is uploaded, this will be checked by the external checker.
3. External checker checks records for incongruencies between sections. They will also note if anything else seems to be missing. Not all questions can be checked for completeness and incongruency because of the large volume of questions. The data checker checks at least these points:
   a. Completeness of all informed consent questions, for household head and household members.
   b. Accuracy of household code.
   c. Congruency in age and sex between household listing and section E and the age and sex of each selected household member in their interview.
   d. Completeness of registration any minor/major procedures per household member (section B)
   e. Completeness section C questions about transportation means
   f. Completeness section D questions about deaths and minor/major procedures
   g. Completeness section E questions about sex / age / consent / general health / alcohol / smoking / medicine.

h. Completeness sections F-K: any problems and if so: where, specifics, visit health facility, still bothering? For section J: LUTS score if participant is male over age of 12.
i. Completeness section L: period stopped, family planning, current pregnancy, months, complications, fundel consent, fundel height, number of pregnancies including/excluding current, neonatal death in last 12 months, outcomes of all pregnancies.
j. Completeness section M: does presence or absence of wounds correspond with earlier sections? Is amount/size/picture filled in?
k. Completeness section N: is BP taken for all participants above 18yrs of age?

4. If there are issues with a record, the external checker will send a query in the WhatsApp group for the team in this format: EA / Interviewer name / Household number / Participant number / Question. Questions should be clear and answerable with yes/no. One query message per record. This query will also be recorded in a Query file on the Dropbox.
5. If there are no problems, this is also feedbacked within the group with following format: Records correct: EA / household numbers, EA / household numbers etc.

Back in the field

1. When Data Checker gets feedback that actions are needed on records in the cluster, he relays these questions to the interviewer.
2. If question is easily answered and doesn't require much extra questioning of household, Data checker immediately feedbacks answer to query through direct response to query in data checking WhatsApp group for that team.
3. If additional interviewing is necessary, the interviewer goes back to the household and reopens the interview to change or add what is missing/wrong.
4. If this is the case: Data Checker uploads corrected record and feedbacks through direct response to query in WhatsApp group that his was done.

Out of the field again

1. If question was answered but record not changed in the field: external checker changes record through RedCap website.
2. If record was changed in the field: external checker checks record again.
3. External checker lets internal checker know if query is resolved.
4. External checker inputs response to query in query Excel on the Dropbox.

Data Management plan

This is the data management plan of Prevalence Study on Surgical Conditions (PRESSCO) 2020. This data management plan is most likely under continues construction until the end of the study so that data management team is able to optimize the data management plan to the circumstances that will occur during period of this study.

Project data flow

All primary quantitative data collected from the household survey will be collated by the study team into the main study REDCap (Research Electronic Data Capture) database hosted at the UMC Utrecht, Utrecht, the Netherlands.

Data base set up

Quantitative data collected from the participants will be collated into the main study REDCap database via validated electronic survey forms which will be developed by the research team of PRESSCO 2020. REDCap is a browser-based, metadata-driven electronic data collection software solution and workflow methodology for designing clinical and translational research databases.

Data collection and transfer

Masanga Hospital, Masanga, Sierra Leone will be the base of the PRESSCO research team. The research teams will travel through Sierra Leone to collect the data at the clusters. Password secured Samsung tablets that will be used to collect the data have mobile internet access. Data will be transferred over the internet using secured data communication protocols. In case the mobile internet connection is disrupted, data will be uploaded as soon as the mobile internet connection is restored. This is possible since the mobile application of REDCap stores the collected data on the device it is installed on when there is no internet connection. All data will be stored automatically and regularly back-ups will make sure that data will never be lost. Databases and web servers of data management systems will be securely hosted in University Medical Center Utrecht, Utrecht, The Netherlands.

Data base security

Data will be collected through the mobile REDCap application that are installed on Samsung tablets. Team members of the research team will get a role-based access to REDCap after they have logged-in using their own username and password. The system will log all data entry steps with timestamps, update reasons and user information. The role-based access to the system will avoid unauthorized data access and prevents users from performing actions that they are not allowed to do. Training will be conducted to all team members of the research team before the start of the data collection. During to data collection, the data will be managed by certain members of the research team that are especially assigned for this task.

All data processing within and beyond the project will be done in compliance with the EU GDPR. Section 10.8 of the signed consortium agreement lays down the processing agreements.

The following general principles will apply to all research conducted PRESSCO’19. Data handling will comply with current national and EU legislation, including: Personally Identifiable Information (PII) is adequately protected. All (pseudonymized) personal data of study participants, is regarded as confidential information. Anonymization/de- identification is conducted appropriately. Ethical review is completed as required

All data storage will be hosted by UMC Utrecht, Utrecht, the Netherlands which maintains highest standards for back-up and security of the system applications and data. Two separate servers will be used, and the database server will be located securely behind a firewall.

Data archiving

At the end of the project, the collected data will be preserved in a secure manner – a continuation of the data storage during the project, meaning encrypted and on a secure server. The connection key to the data will be stored for 5 years for the purpose of control. De-identified data will be kept for a minimum preservation term of 15 years on the secured server of the UMCU, unless otherwise specified in the contracts with the original owners of the routine data in the different participating countries.

User privileges will also be set so that some users may be allowed to export data from the project but will have the data de-identification methods imposed as a means of preventing them from exporting sensitive data, either mistakenly or intentionally.

Data findability and provisions for metadata

Data stored in REDCAP will not contain any participant identifiable data. Each participant will be assigned to a study participant code which was composed by the cluster number, enumerator number, household number and study participant number. This individual code will be used at three different places. Firstly, the enumerators write down the study participant code in the survey in REDCap. Secondly, the study participant code is written down on the consent form. Finally, the study participant code is written down on a business card that is handed over to the study participant at the end of the interview. This business card contains contact details of the study group. If study participant wants to request their given answers, want to withdraw their consent or have any other issues concerning their interview, their record can be found with the study participant code.

Making data openly accessible

A managed access procedure will be developed to allow access to other researchers that wish to use the study data for secondary analysis after the closure of the study. These data will not be made openly accessible in line with national, European and international legal, ethical and privacy concerns. Access to the data will be controlled by principal investigator Håkon A. Bolkan and the senior board of PRESSCO 2020. After the study has been completed and the main study papers are published, researchers can apply to principal investigator Håkon A. Bolkan with proposals to access the study data set for other studies. Access to the data set requires approval from the Steering Committee, which comprises representatives from each consortium partner and is chaired by Håkon A. Bolkan. Researchers will additionally need to sign a Data Sharing Agreement to protect the integrity and confidentiality of the requested data. Any shared data will be further minimized and anonymized as much as possible for the requested purpose.

Tablet Manual

Front view

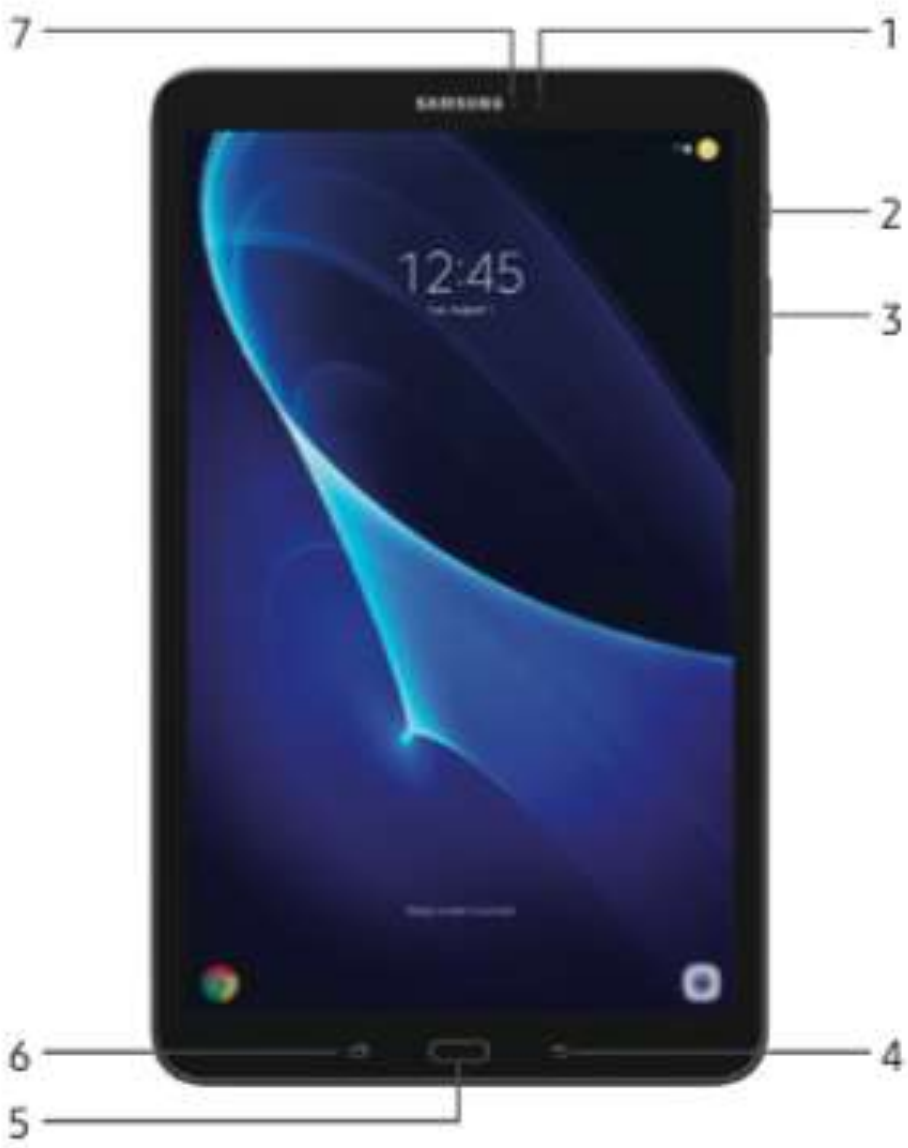

1. **Front camera**: Take self-portraits and record videos of yourself.
2. **Power key**: Press and hold to turn the device on or off, or to restart it. Press to lock or wake up the screen.
3. **Volume key**: Adjust the volume of your device's sounds and audio playback.
4. **Back**: Return to the previous screen or close a dialog box, menu, or keyboard.
5. **Home key**: Return to the Home screen.
6. **Recent apps**: Tap to display recent apps, or touch and hold for Multi window™.
7. **Proximity sensor**: Detect the presence of objects near the device.

How to turn your tablet On and Off

Press and hold the Power/Lock Key that is located on the side of your tablet.

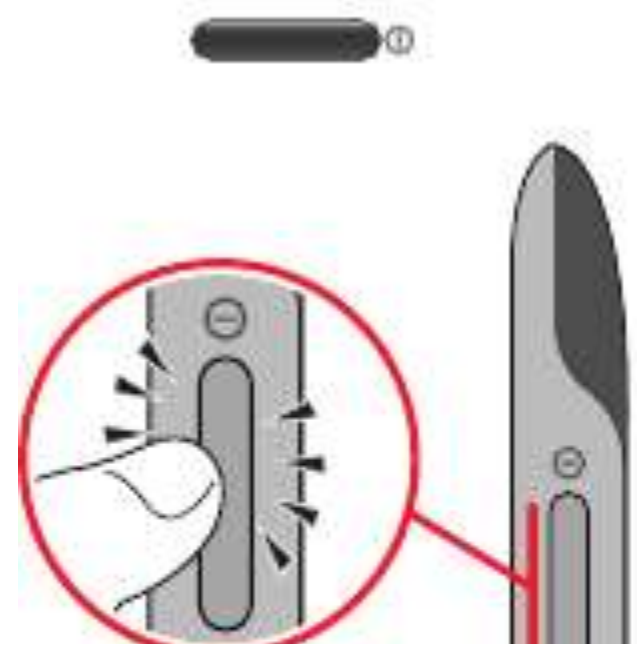

Status bar and battery

The Status bar at the top of the Home screen provides device information (such as network status, battery charge, and time) on the right side and notification alerts on the left.

Status Icons

**Battery full**: Battery is fully charged.	**Mute mode**: Mute mode is enabled.
**Battery low**: Battery is low.	**Wi-Fi active**: Wi-Fi is active.
**Battery charging**: Battery is charging.	**Wi-Fi available**: A Wi-Fi network is available.

Battery Indicator

The Battery icon in the Status Bar shows the battery power level. When battery power is 15% or less, your device prompts you to charge the battery. If you continue to operate the device without charging, the device powers off. The tablet device comes with a Wall/USB Charger to charge your device from any standard power outlet.

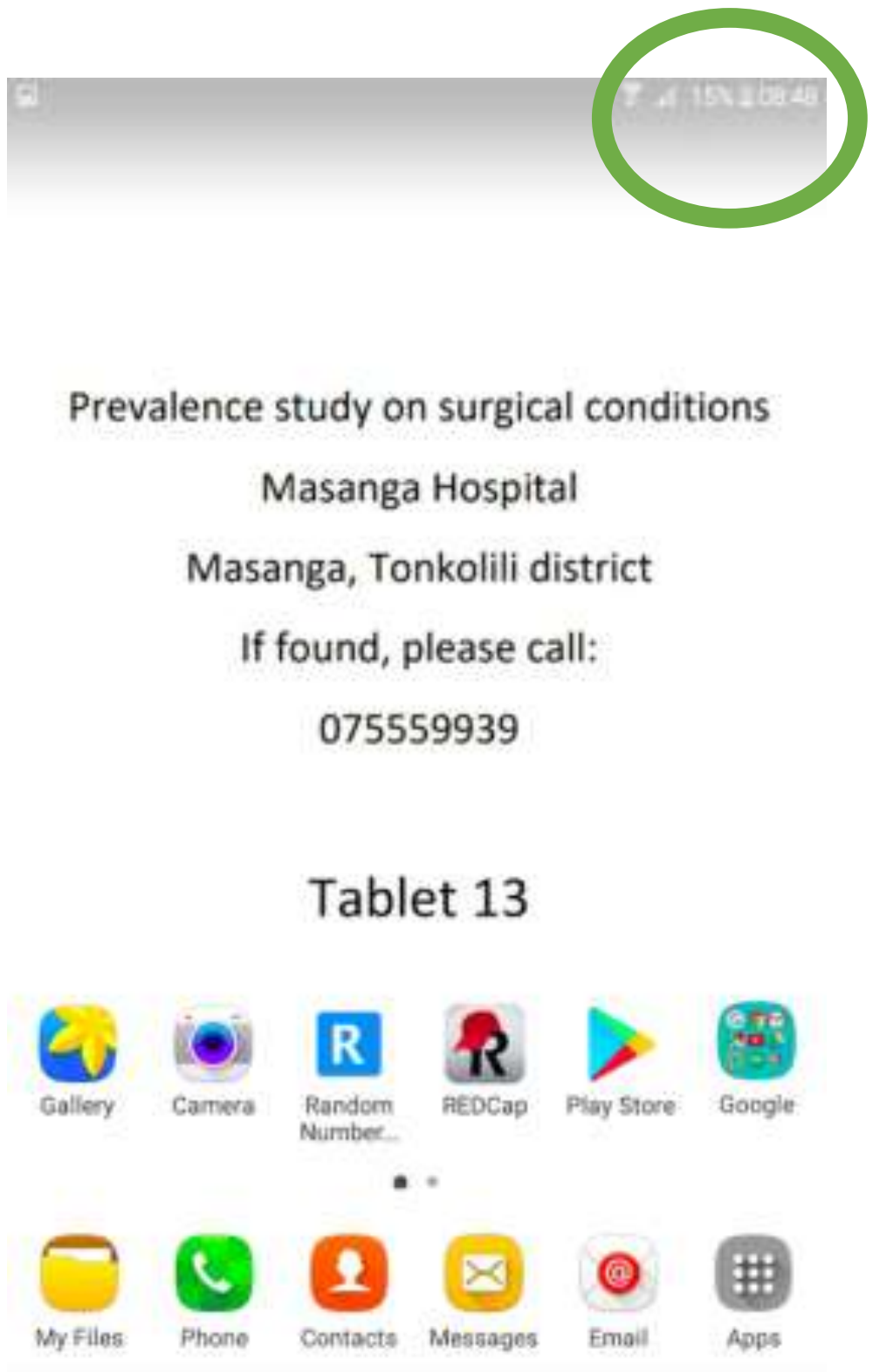

Navigation

How to navigate your device

Touchscreen Navigation

Please note that a touch screen responds best to a light touch from the pad of your finger or a capacitive stylus. Using excessive force or a metallic object on the touch screen may damage the tempered glass surface and void the warranty.

Tap

Lightly tap items to select or launch them.

- Tap the keyboard to enter characters or text.
- Tap an item to select it.
- Tap an app shortcut to launch the application.

Touch and Hold

Touch and hold items on the screen to activate them.

- Touch and hold a widget on a Home screen to move it.
- Touch and hold a field to display a pop-up menu of options.

Swipe

Lightly drag your finger vertically or horizontally across the screen.

- Swipe the screen to unlock the device.
- Swipe the screen to scroll through the Home screens or menu options.

Unlocking the tablet

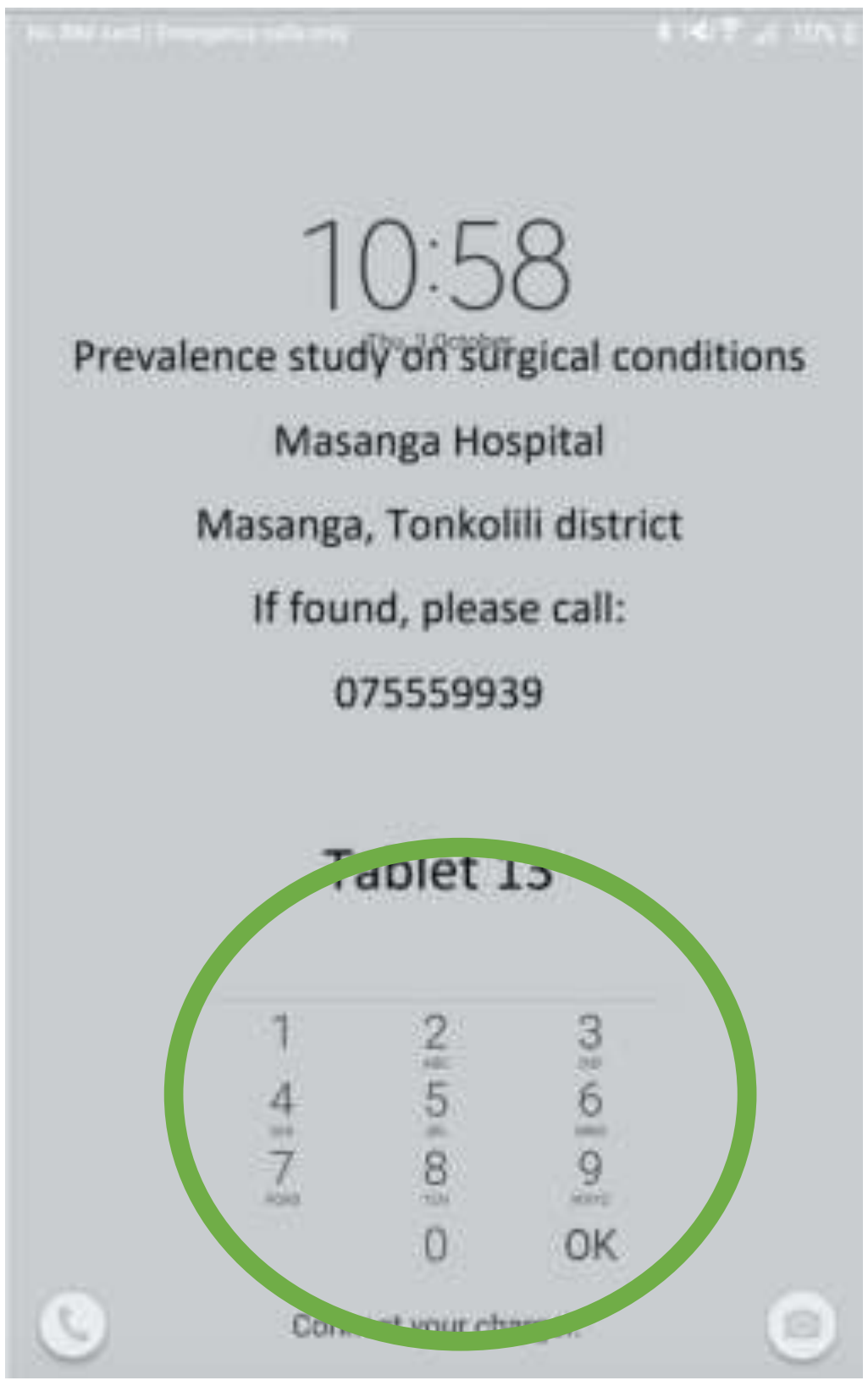

When you press the power button and the tablet comes on, this is what you will see. The tablet is locked, so only the ones with the correct code will be able to use the tablet.
You will get the correct code from us when you are going to use the tablet.
Tap lightly on the number that is the code and then press OK.

How to open applications

If the application you want to open has a shortcut on the home screen, you can open it directly from the home screen by lightly tapping the application.

If the application does not have a short cut on your home screen, you will have to press "Apps", and you can press the application you want use from there.

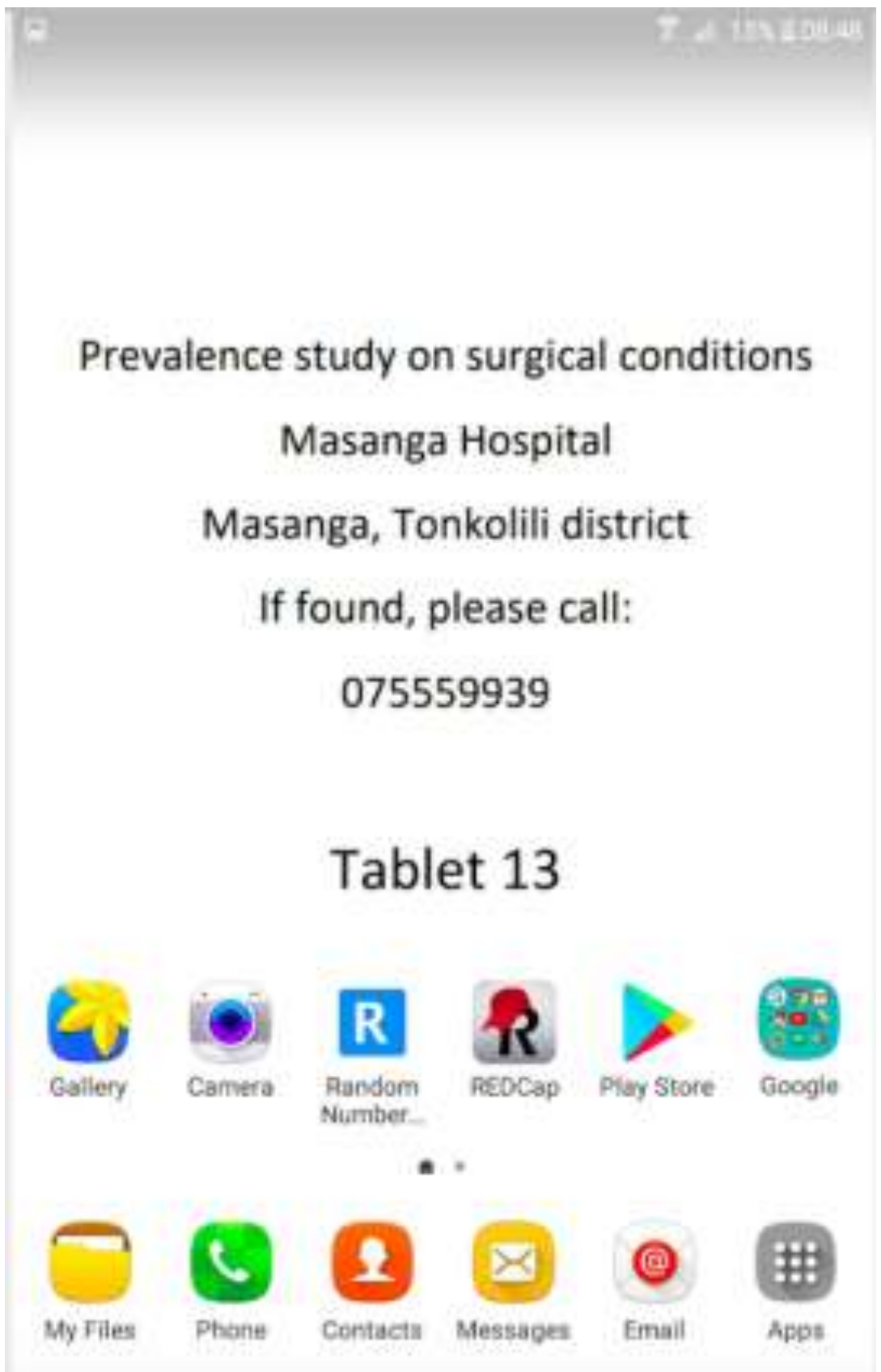

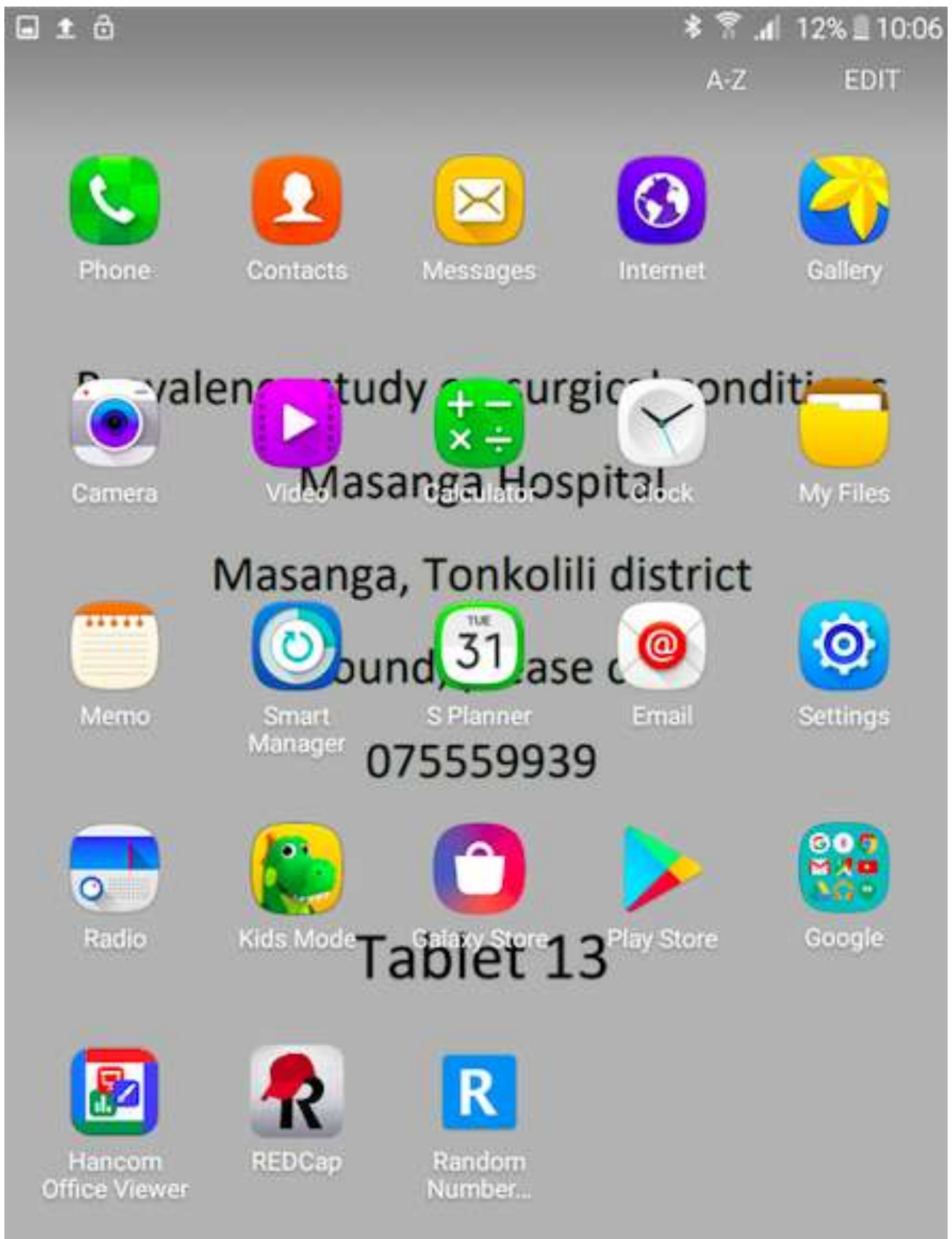

CAMERA

There will be situations where you will have to take pictures of wounds. Go to home screen by clicking the home-screen-button. Then press the camera-app.

How to focus the camera:
When taking a picture its important to have the correct focus to make sure that the quality of the photo is good. To focus the shot, tap the screen on the area you want to take a photo of.

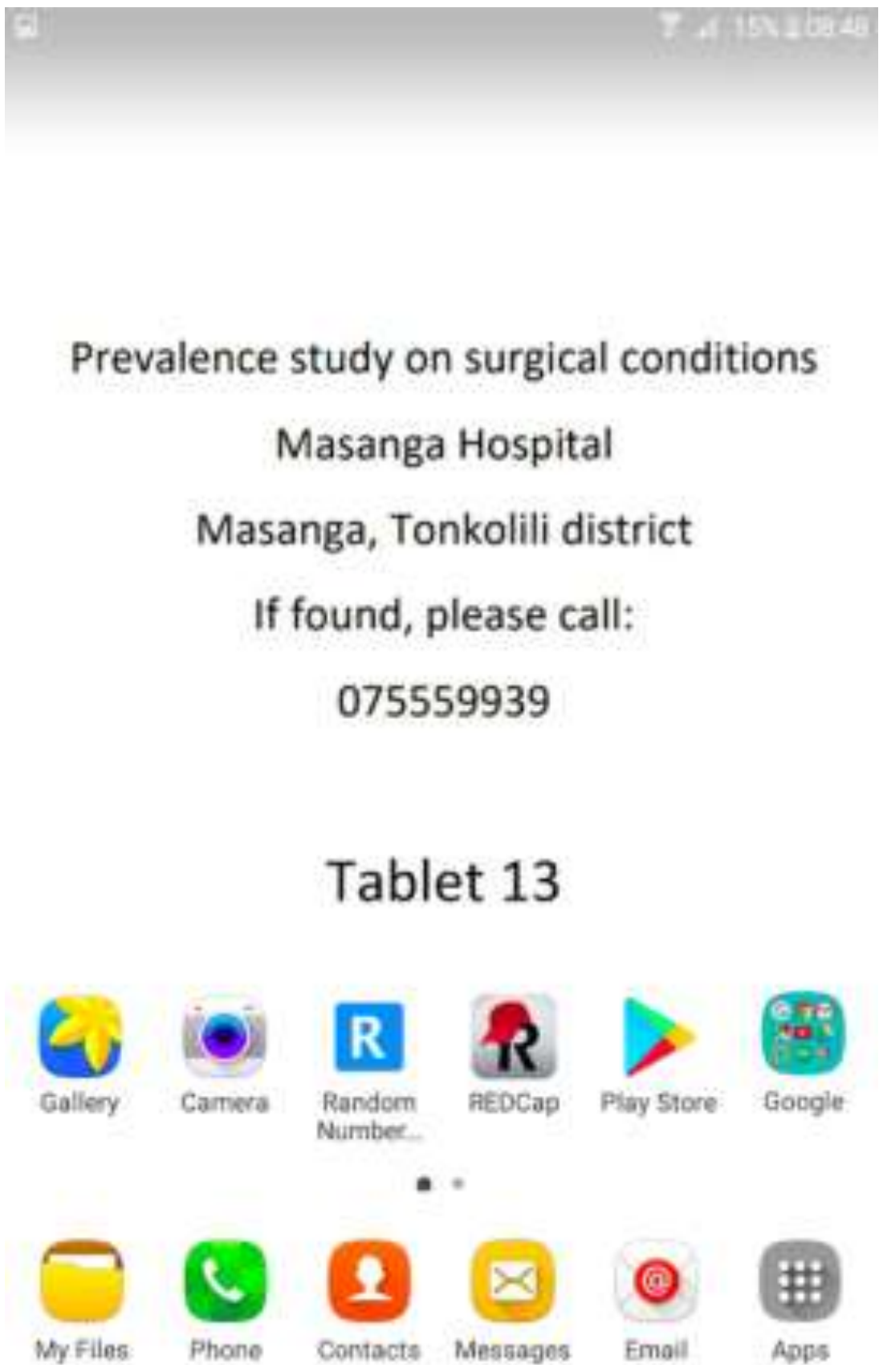

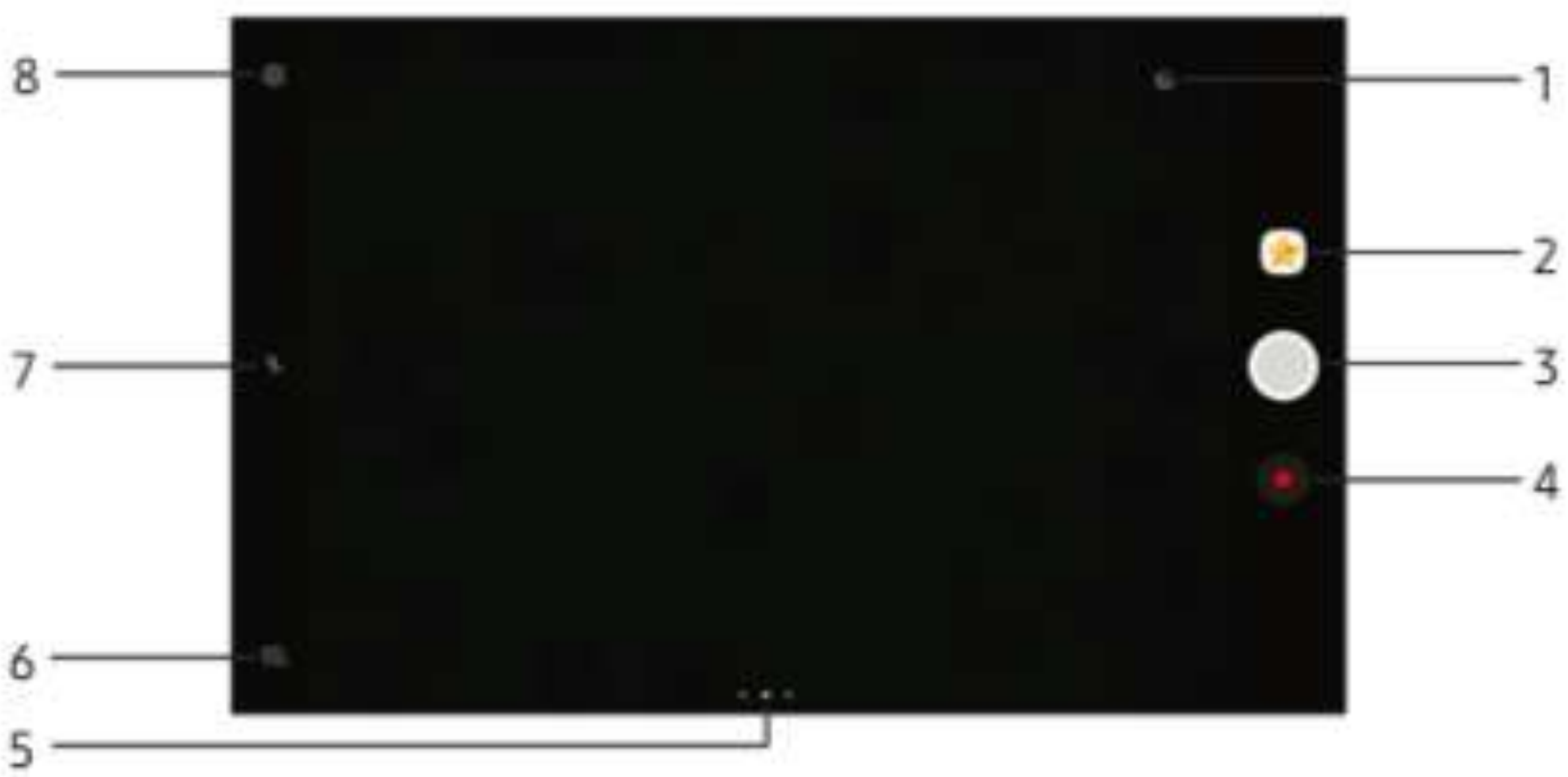

1. **Beauty**: Take a picture with lightened faces for gentler images.
2. **Gallery**: Open the Gallery to view photos and videos.
3. **Take a picture**: Take a picture.
4. **Record video**: Record a video.
5.
6. **Switch cameras**: Switch between the rear and front cameras.
7. **Flash**: Activate or deactivate the flash.
8. **Settings**: Configure other camera settings.

Random calculator

Minimum: 1
This should always say 1.

Maximum: Enter the total number of household members.

How many do you need: 2
This should always say 2.

Press "GENERATE", and the calculator will randomly pick out two numbers for you.

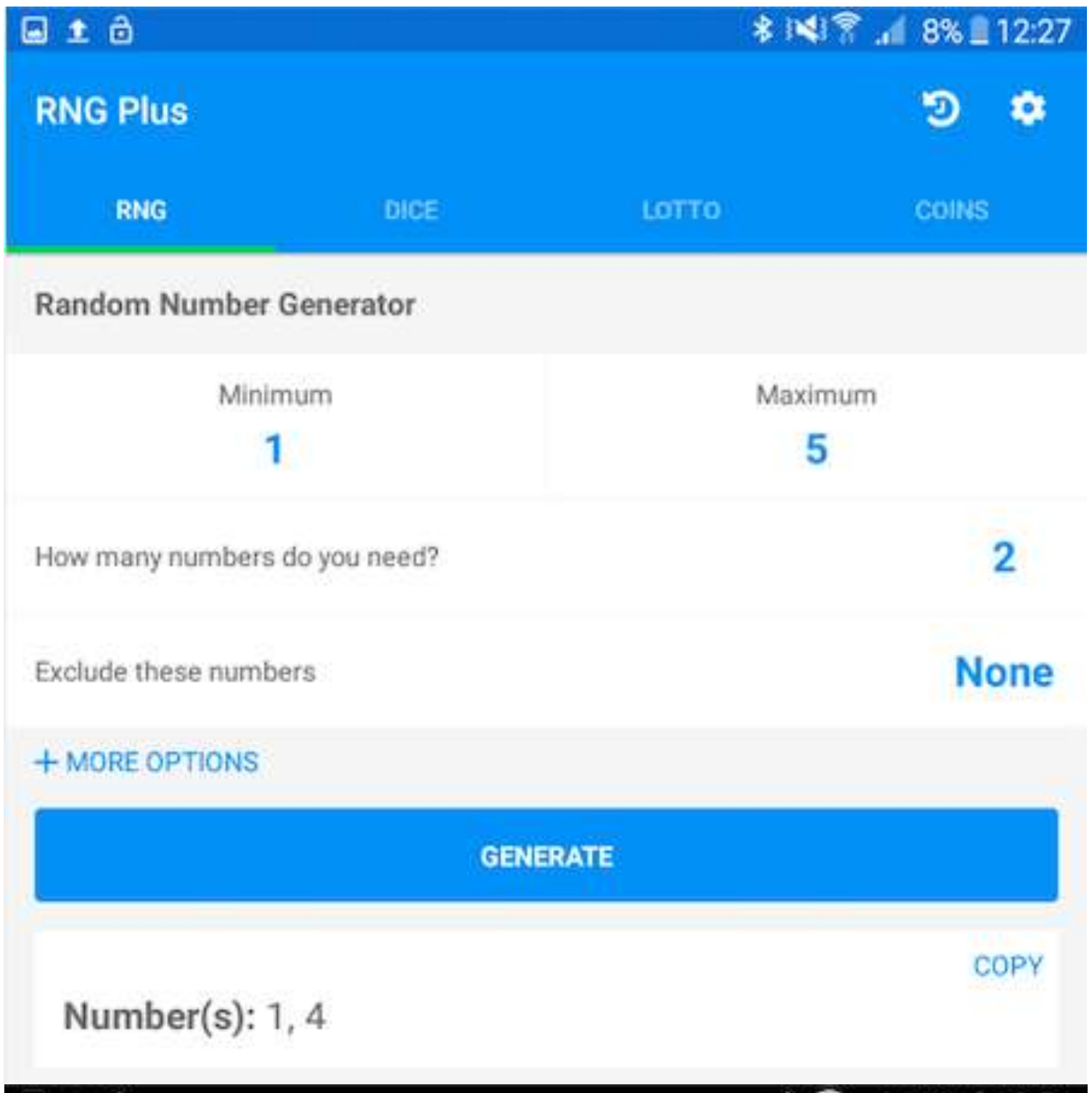

REDCap

Redcap is the program we will be using to collect the data in the data collection.

You will receive a number/username and a PIN to log on to REDCap

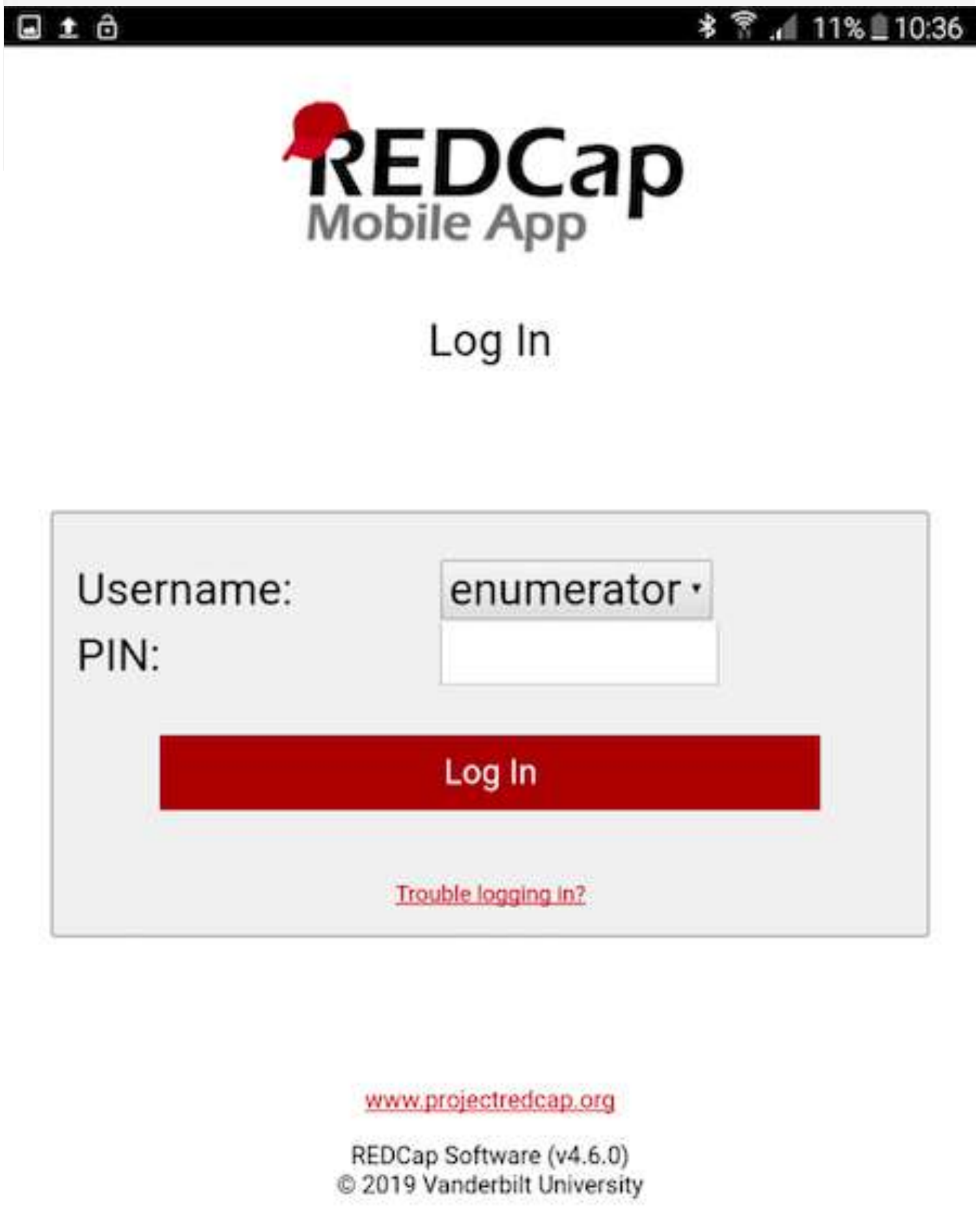

Referral letter

Date of referral:

Place of referral:

Masanga Medical Research Unit	Name patient:
Masanga Hospital	Age:
Tonkolili District, Sierra Leone	Sex: M/F
Referring health worker:	Advised to be seen:
☐Dr. ______________________	☐Urgently
☐Surgical CHO _______________	☐Within 24 hours
☐Nurse _____________________	☐Within a week
☐ _________________________	☐Without any urgency

Dear colleague,

Hereby, I would like to refer this patient to your health care facility for further evaluation and possible treatment.

Reason for referral:

____

Relevant medical history:

____

Current medication:

____

Additional information:

____

Kind regards, Signature:

on behalf of Masanga Hospital.

Patient information sheet

Prevalence Study on Surgical Conditions (PRESSCO) 2020 in Sierra Leone

Invitation to participate in a research project

You are invited to participate in a research project. I work for the Masanga Medical Research Unit in collaboration with Statistics Sierra Leone. We are trying to find out if there are enough doctors in this area, specifically if there are enough surgeons. We won't be offering medical care right now, but we hope that the information you provide will help improve services in the future. All of your responses will be confidential. You have the right to not participate in the survey, or to stop at any time during the interview without any consequence.

Summary

- By taking part in this survey, you help us investigate the need for surgical care in this area
- We will ask question about your medical history
- We do not offer medical care right now, but hope to establish improvement of available healthcare in this area in the future
- Participation is voluntary and consent can be withdrawn at any moment
- Privacy of the participant will always be honoured, now and in the future
- Contact information of the research group will be shared with every participant

What is the interview about?

We will ask questions such as whether someone ever had a wound, a broken bone, a mass or problems concerning maternal health and the health of newborns. Some participants might be asked to be examined if they have a mass in their groin. If a participant currently has a wound, we might want to take a picture of the wound.

For woman above the age 12: we will ask about female related complaints, family planning and pregnancies. In the mournful case that a baby of a participant died, we also would like to ask some questions about this.

For men above the age 12: we will ask about urinating habits and possible problems related to this. We might want to ask the participant to pee in a tube for us.

For the head of the household

I would like to ask you some questions about the people who live in this house. I will also ask you questions about household members that passed away during the last twelve months. I will randomly choose two people from your household and ask them more detailed questions about their health. Informed consent from you and the two randomly selected household members will be obtained. The whole interview will take 45 to 60 minutes.

Foreseeable benefits and predictable risks and burdens of taking part

The asked questions could be experienced as an invasion on someone's privacy. Participating in this research project will take some time out of someone's day. If medical problems are identified during the survey, the participant will be informed about the condition and advised about possible treatment options. Depending on the urgency, referral to the local health facility will be arranged or a letter of referral will be issued.

Voluntary participation and the possibility to withdraw consent

Participation in the project is voluntary. To take part in this study, a declaration of consent needs to be signed. Consent can be withdrawn without reason at any time. Personal data concerning health will be deleted if a withdraw of consent is received. If there is a wish to withdraw consent, contact the research team by telephone on **075559939** or send an email to **masangaresearch@gmail.com**.

What will happen to personal data concerning health?

Any personal data concerning your health will only be used as described in the purpose of the project. You have the right to access information that has been recorded and the right to stipulate any errors in the information that is recorded. Your privacy will be honored at all times and information concerning your health will not be shared with others. All information will be processed and used without your name. A code links your name and the personal data concerning your health via an identifier list. This linking code will never be released and will be deleted after five years. Only the participant and the research group will have access to this code. The research group will provide the participant with their personal code.

In accordance with the General Data Protection Regulation the controller, the Norwegian University of Science and Technology and the project manager Håkon Angell Bolkan are independently responsible to ensure that the processing of your personal data concerning health has a legal basis. This project has legal basis in accordance with the EUs General Data Protection Regulation, article 6a, article 9 nr.2 and your consent. You have the right to submit a complaint on the processing of personal health data concerning health to the Norwegian Data Inspectorate (Telephone (+47) 2239 6900).

Sharing of personal data and transfer of personal data abroad

By agreeing to participate in the study, a participant is also consenting that their information regarding their health collected from the interview and a possible examination can be transferred to another country as a part of research collaboration and publication.

Ethical approval

The Norwegian Regional Committee for Medical and Health Research Ethics has reviewed and approved the Research Project (REC 2019/31932), as well as the Masanga Research Unit's Scientific Review Committee (MMRU-SRC-009-2019), and the Sierra Leone Ethics and Scientific Review Committee (SLESRC2019/October/03).

Complains and feedback

In case you have complains about the research project or any other feedback, please contact us. Contact details are listed below. If you want to speak to Sierra Leone Ethics and Scientific Review Committee dial: **078366493** or send an email to: **efoday@health.gov.sl**.

Contact information

The research group can be contacted any time by telephone on **075559939** or by sending an email to **masangaresearch@gmail.com**.

Abbreviations

BPH	Benign Prostate Hyperplasia
CD	Caesarean Delivery
CHO	Community Health Officers
CS	Caesarean Section
DCP	Disease Control Priorities
EA	Enumerator Area
EVD	Ebola Virus Disease
IPSS	International Prostate Symptom Score
LCoGS	Lancet Commission on Global Surgery
LLMICs	Low- and Lower- and Middle-Income Countries
LMICs	Low- and Middle- Income Countries
LUTS	Lower Urinary Tract Symptoms
MD	Medical Doctor
MMRU	Masanga Medical Research Unit
MoHS	Ministry of Health and Sanitation
NGO	Non-Governmental Organization
NTD	Neglected Tropical Diseases
PHU	Primary Healthcare Unit
QoL	Quality of Life
ROH	Rate of Homogeneity
SDG	Sustainable Development Goals
SOSAS	Surgeons over Seas Assessment of Surgical need
SSA	Sub-Sahara Africa
TEAP	Transurethral Ethanol Ablation of the Prostate
UHC	Universal Health Coverage
UNFPA	United Nations Population Fund
VPSS	Visual Prostate Symptom Score

Definitions

Burn	Wound of the skin caused by heat, chemical or electrical exposure
Cluster	The geographic area where the assigned households are located and where the interviews are hold. In this article, cluster and enumerator area have a similar meaning.
Essential Surgical Procedure	One of the 44 surgical procedures that were deemed as essential on the basis that they address substantial needs, are cost-effective, and can feasibly be implemented. Provision of essential surgical procedures would avert an estimated 1.5 million deaths a year, or 6-7% of all avertable deaths in low- and middle-income countries.
Groin hernia	A weakness in the muscle and tissue of the groin.
Head of household / Household representative	A male or female member of a household recognized as such by the other household members. The head of the household is generally the person who has the economic and social responsibility for the household. All relationships in the household are defined with reference to the head.
Household	Every person who eats from the same pot as the head of household and slept in the household the night before the visit of the enumerator.
Major Surgical Procedure	Surgical procedure that is frequently performed in a first level or referral hospital and usually requires a type of anesthesia.
Met surgical need	The number of surgeries that are actually performed on yearly basis.
Minor Surgical Procedure	Surgical procedure that is frequently performed, and can be safely done, in a community facility or primary health center as described by the third edition of the Disease Control Priorities (DCP3).
Primary health care unit	Healthcare facility with overnight beds and 24-hour staff (as would be needed for e.g., normal vaginal delivery).
Private health facility	Collection of heterogeneous facilities; independent hospital or clinic, informal facility or formal for-profit entity, that may include (un)licensed providers, and non-profit, faith-based and non-governmental organizations.
Public health facility	Facility where health care is usually provided by the government through national health care systems.
Referral and specialized hospital	A facility that has advanced or subspecialized expertise for treatment of one or more surgical conditions, not usually found at lower-level facilities[1].
Surgical Care	The provision of operative, perioperative and non-operative management for all surgical conditions. Surgical care also includes preoperative assessment (including the decision of whether to operate or not), with

	the provision of safe anesthesia, and postoperative care.
Surgical Condition	Any disease, illness or injury in which surgical care can potentially improve the outcome.
Surgical need	The number of surgical procedures that should ideally be performed on yearly basis per 100,000 inhabitants for a particular country in order to keep its inhabitants unrestrained from diseases and disabilities and well recovered from trauma occurring on a yearly basis.
Surgical Procedure	Suturing, incision, excision, or manipulation of tissue; or other invasive procedure performed in an operating theatre or procedure area regardless of anesthesia type or surgical provide.
Traditional Medicine	The knowledge, skills and practices based on the theories, beliefs and experiences indigenous to different cultures, used in the maintenance of health and in the prevention, diagnosis, improvement or treatment of physical and mental illness.
Unmet Surgical need	The surgical need, subtracted the met surgical need.
Wound	‘Open skin’, regardless of size or aspect or etiology.
