# Supplementary material for: Caesarean section rates and obstetric practices in Sierra Leone before, during, and after the Ebola epidemic: Results from a nationwide household study
Source: Womens Health (Lond). 2026 Sep 21;22:17455057261487141. doi: 10.1177/17455057261487141 (PMC13731882; doi:10.1177/17455057261487141)
Supplement: Supplementary file 3 [file sj-pdf-3-whe-10.1177_17455057261487141.pdf]

Prevalence Study on surgical conditions 2019

SOSAS Repeat / Lower urinary tract symptoms / Groin hernia / Chronic wounds / Surgical volume / Maternal and perinatal health

Questionnaire version 1.0 (final) | 2nd September 2019

Håkon A. Bolkan[a,b,c] (Principal investigator)
Martin Grobusch[d,e] (Co-Principal investigator)
Osman Sankoh[f] (Co-Principal investigator)
Jurre van Kesteren[g,h,i]
Mia Nyeng Østensen[a]
Sofie Mack Løvdal[a]
Karel Lindenbergh[g]
Jonathan Vas Nunes[d,e]
Daniël van Leerdam[c,k]
Josien Westendorp[a,c,o]
Hanna Mathéron[d,e]

Collaborators

Alex van Duinen[a,b,c]

Reinou S. Groen[m,r]

Diede van Delft[e]

Dag Halvorsen[a,b,c]

Michael Kamara[c,p]

Daniel Boateng[o]

Helena Bertilsson[a,b]

Thomas Ashley[c,n,s]

Frank Bloemers[h]

Joyce Browne[o]

Marcus Rijken[o]

Kerstin Klipstein-Grobusch[o]

Thaim Kamara[n,p,q]

Jenny Löfgren[l]

Jaap Bonjer[h,i]

Janine Martens[t]

Daan van Herwaarden[t]

Affiliation / Institute

a. NTNU, Norwegian University of Science and Technology, Trondheim Norway
b. St. Olavs Hospital, Trondheim, Norway
c. CapaCare, Trondheim, Norway
d. Universiteit van Amsterdam, Amsterdam, The Netherlands
e. Masanga Medical Research Unit, Tonkolili district, Sierra Leone
f. Statistics Sierra Leone, Freetown, Sierra Leone
g. Faculty of Medicine, VU Vrije Universiteit, Amsterdam, The Netherlands
h. Department of Surgery, Amsterdam UMC, Amsterdam, The Netherlands
i. Global Surgery Amsterdam, Amsterdam, The Netherlands
k. KIT, Royal Tropical Institute, Amsterdam, The Netherlands
l. Karolinska Institutet, Solna, Sweden
m. Johns Hopkins School of Medicine, Baltimore, USA
n. Ministry of Health and Sanitation, Freetown, Sierra Leone
o. UMC Utrecht, Julius Center, Utrecht, The Netherlands
p. College of Medicine and Allied Health Sciences, University of Sierra Leone, Freetown.
q. Department of Surgery, Connaught Hospital, Freetown, Sierra Leone
r. SOSAS – Surgeons Over Seas, New York, USA
s. Kamakwie Wesleyan Hospital, Sierra Leone
t. Faculty of Medicine, University of Utrecht, Utrecht, The Netherlands

INTRO: HOW TO USE THIS QUESTIONNAIRE

Items in bold are the new questions.
Under the bold items the questions to be asked.
[Items in italics are instructions to the interviewer.]

Paragraphs in the boxes are explanations during the interview for the respondent. Read these out loud and be sure that the person understands the explanation.

A. HOUSEHOLD INFORMATION (TAB: Household)

A1. Number of visits: _____ 1 _____ 2 _____ 3 _____ 4 ☐
[Check off each visit to the household, check the box at the end if this is a replaced household]

A2. Village # OR Cluster #: ____________________

A3. Village Type: ____ Rural _____ Urban _____ Slum

A4. Household Code: _____________

A5. Interviewer Name: _____________

'Good morning/evening. My name is ______________. I work for the Masanga Medical Research Unit, that works with Statistics Sierra Leone [*show the information letter*]. We are trying to find out if there are enough doctors in this area, specifically if there are enough surgeons. A surgeon is a medical doctor who performs operations in a hospital.<br><br>To find out if there are enough doctors taking care of these surgical problems in your village, we'd like to ask you and some other members of your household some questions. We will ask questions about your health, such as whether members of your household ever had wounds, broken bones, or masses. We will also ask if anyone in this household have died during the last 12 months and if they had any operations performed. We will measure blood pressure of each adult participant, as well as do examination and take anonymized photos of wounds. We won't be offering medical care right now, but we can offer a referral note to those that need medical attention. We believe the information you provide will help improve health services in the future.<br><br>This survey will take about 45 minutes to 1 hour. All of your responses will be confidential. You have the right to not participate in the survey, or to stop during the interview. First, I will ask you some questions about the people who live in this house. After that, I will randomly choose two people from your household and ask them more detailed questions about their health. This information is confidential and nobody will find out what answers you gave, other than me and my research team. We will not collect your name or address. We have an information sheet for you and we will obtain your consent to participate. Separate consent will be asked from the household members who will participate. Do you have any questions at this moment?

A6. Informed consent:
Would you like to participate in this survey?
___ Yes
___ No (If no, what is the reason? _____________)
(no time / no willingness / no reason / no seen benefit / other: explain….)

[Without informed consent you cannot proceed. Make sure the person understands the purpose of this survey. If they don't want to participate, ask why and mark this.]

B. LIVING HOUSEHOLD MEMBERS

The following questions will be about your household members. I want to include every household member that normally eats from the same cooking pot, and slept here last night. We will start the information of the oldest household member and finish with the youngest, also babies, neonates and disabled household members need to be listed in order of their age.

[Fill in all the household members' age and sex in the table, ordered by age, the oldest household person first. Also the household members who are disabled or ill should be mentioned. At the end of the list of household members you need to ask specifically for the newborns and babies in the family and collect the information for each of them in individual tabs.]

B0. Number of household members:
In total, how many people live in your household? _____

Tabs	**B1. Age:** (years*)	**B2. Sex:** Male/Female	**B3. Surgery last 12 months** y/n
1			
2			
3			
4			
5			
6			
7			
8			
9			
10			
11			
12			
13			
14			
15			
16			
17			
18			
19			
20			
21			
22			
23			
24			
25			
26			
27			
28			
29			
30			

*[*Age for babies:*
<3 months = 0
3 - 6 months = 0.25
6 - 9 months = 0.5
9 - 12 months = 0.75
12 - 24 months = 1 etc.]

Step 1. Check if all information is filled in for all the household members, remember the definition: all the persons eating from the same pot and sleeping in that house
Step 2. Make sure that for each household member the age and the sex has been provided.
Step 3. Check the order of the household members. This should be starting with the oldest person and ending with the youngest person, make sure you ask for neonates/babies.
Step 4. Make corrections before you go to step 5.
Step 5. Remember the total numbers of living household members. Open your Random Generator on your tablet: fill in for 'Min':1 and for 'Max' : the number of living household members. Press Generate. The first number which appears corresponds with the number of the household member to interview. Repeat the procedure also to assign the person for the second interview.

Tab number of the assigned household member for the first interview: _____
Tab number of the assigned household member for the second interview: _____

Household members after surgical procedure

EXPLAIN:
Continuing in this section where we have some specific questions about household members who had an operation in the last 12 months.

The following questions will be about your household members. I want to include all from the list we just made that underwent a surgical procedure in the last 12 months. We will start by making a list of all these household members. We would like to include household members from all ages.

Surgical procedure is also known as an operation.

[Surgical procedure: '... suturing, incision, excision, or manipulation of tissue; or other invasive procedure performed in an operating theater or procedure area regardless of anesthesia type or surgical provider']

[FOLLOWING QUESTIONS ONLY IF THERE WAS A HOUSEHOLD MEMBER WHO UNDERWENT A SURGICAL PROCEDURE IN THE LAST 12 months.]

B4. Minor procedures:
Did the household member undergo any of the following (minor) procedures in the last 12 months:

- incision and drainage of an abscess
- circumcision (if male)
- normal delivery (so no caesarean birth, vacuum extraction or forceps delivery)
- suturing of wounds
- conservative treatment of fractures (so no operation)
- tooth extractions
- other minor surgeries (e.g. uninary catherization, surgical dressings).

[Fill in in table below. All minor procedures are to be included, irrespective of where the procedure was performed (e.g. both hospital, traditional healers, primary health units)]
[Other minor surgeries: any procedures with manipulation of tissue where no anesthesia was used}
[Anesthesia: putting to sleep of a patient during the operation, or numbing of body part, usually with an injection in the back or the specific body part]

**HHM**	**I&D:** Y/N	**Circumcision (male):** Y/N	**Normal Delivery:** Y/N	**Suturing:** Y/N	**Punctures** Y/N	**Conservative Treatment Fractures** Y/N	**Tooth extraction** Y/N	**Other minor surgery** Y/N	**B4. Major Surgery** Y/N
1									
2									
3									
4									
5									

B5. Major procedures:
Did the household member undergo a major procedure that required anesthesia in the last 12 months?
___Yes
___ No
[Fill in in table above]
[Anesthesia: putting to sleep of a patient during the operation, or numbing of body part, usually with an injection in the back or the specific body part]

[if 'No' to B4, the rest of section B can be skipped]
[if 'Yes' to B4 a 'tab' for each household member who underwent surgery will be opened for further specific questions *]*

[FOLLOWING QUESTIONS ARE FOCUSSED ON THE HOUSEHOLD MEMBERS WHO UNDERWENT A MAJOR SURGICAL PROCEDURE IN THE LAST 12 MONTHS. FOR EVERY HOUSEHOLD MEMBER WHO UNDERWENT A SURGICAL PROCEDURE A NEW 'TAB' NEEDS TO BE OPENED]

We will now ask more detailed question about each operation that the household has undergone.

B6. Frequency of surgical procedure:
How many times were you/the household member operated in the last 12 months?
___ number
[if is answered with multiple procedures, fill out the information for each of these surgical procedure in the tabs]

B7. Type of surgical procedure:
Would you be able to tell what kind of surgical procedure was done for you/the household member in the last 12 months?
Dropdown list with recognizable surgical procedures

General Surgical

- Appendectomy
- Galbladder operation
- Colostomy
- Abdominal operation, other
- Groin hernia
- Hydrocele
- Orchidectomy
- Excision of mass/lump/growth
- Heart operation
- Surgical wound debribement

Obstetric, gynecologic, and family planning

- CS
- Tubal ligation
- Hysterectomy, pregnancy related

- Hysterectomy, non-pregnancy related
- Myomectomy
- Uterus prolaps operation
- Vasectomy
- Obstetric fistula

Injury

- Chest drain
- Fracture reduction, conservative
- Fracture reduction, surgical
- External fixator
- Amputations
- Skin grafting

Nontrauma orthopedic

Dental procedures

- Dental operation

Congenital

- Cleft lip
- Club foot
- Hydrocephalus
- Anorectal malformations

Visual impairment

- Eye operation

OTHER

B8. Localization of surgical problem:
Where in the body was the surgical procedure done?

	Problem 1	Problem 2	Problem 3	Problem 4	Problem 5
Face, Head, Neck					
Chest, Breast, Back					
Abdomen					
Groin, Genitalia, Buttocks					
Extremities					
Not sure / unknown					

B9. Type of surgical problem:
Tell me what problem you/the household member had that needed a surgical procedure.
[Fill in the type of problem that suits best with the undergone surgical procedure, more answers could be possible, e.g. 'Injury' and 'Wound' or 'Burn' and 'Deformity (acquired)']

	Problem 1	Problem 2	Problem 3	Problem 4	Problem 5
Injury					
Burn					
Wound					
Problem related to pregnancy or childbirth					
Gynecological					
Abdominal distention or pain					
Inability to urinate					
Mass or growth (soft)					
Mass or growth (solid)					
Deformity (congenital)					
Deformity (acquired)					
Hernia					
Dental					
Visual					
Other					
Unknown					

[Injury	*The person obtained a problem after an accident (e.g. broken bone after fall, or wound after motorcycle crash). Type of injury is not important here.*
Burn	*The person obtained a burn after exposure to fire or boiling water*
Wound	*Open skin; sometimes leaking blood, pus or liquid*
Obstetric	*Bleeding and/or disease around childbirth*
Gynecological	*Problem with the menstrual cycle, the womb or bleeding not related to childbirth*
Abdominal	*The person had pain or distention in the belly, or problems with eating or passing stool*
Mass or growth (soft)	*A mass or growth which feels soft and can be manipulated (e.g. lipoma, hernia)*
Mass or growth (solid)	*A mass or growth which feels firm and usually more difficult to manipulate (e.g. hydrocele, tumor)*
Deformity (congenital)	*The person is born with the problem (e.g. club feet, cleft lip)*

Deformity (acquired) The person had a (spontaneous – not after trauma) deformity of body or limbs later in life (e.g. chronic osteomyelitis)
Dental The person had a problem with the teeth
Visual The person had a problem with eyesight]

B10. Elective or emergency surgical procedure:
Did you/the household member need the surgical procedure urgently due to a rapid onset of severe pain or distress?

	Surgery 1	Surgery 2	Surgery 3	Surgery 4	Surgery 5
Yes					
No					
Unsure / does not remember					

[FOLLOWING QUESTIONS ARE FOCUSSED ON THE LOCATION OR SETTING OF THE SURGICAL PROCEDURE. ALL DIFFERENT PROCEDURES THAT THE HOUSEHOLD MEMBER UNDERWENT NEED TO BE FILLED IN THE TABLE.]

B11. Location of the surgical procedure (Q I):
Was the surgical procedure done at one of the following facilities?

DROPDOWN MENU WITH THE LIST OF SURGICAL FACILITIES PREVIOUSLY IDENTIFIED
- We can categorize these in districts/cities/towns to make it easier to go through the list
- We add an OTHER option

B12. Other surgical facility (Q I)
Where was the surgical procedure performed? *[Only if 'other' is answered in B7]*

	Surgery 1	Surgery 2	Surgery 3	Surgery 4	Surgery 5
Government facility					
Private facility					
Traditional healer					
Other					
Unsure / does not remember					

B14. After undergoing the surgical procedure, does the problem still impact your daily life?

	Problem 1	Problem 2	Problem 3	Problem 4	Problem 5
The condition is not disabling					
I feel ashamed					
I am not able to work					
I need help with transportation					
I need physical help with daily living					

[Disability: a physical problem that impacts your life, or makes it difficult to carry out your daily activities.]
[this question is not meant in a financial way but rather if a patient needs physical help with transportation (wheelchair or walking stick or can not walk small distances) or daily living (such as getting out of bed, brushing teeth, eating]

'Thank you very much for answering these questions. Let me go quickly over the survey to check everything.'

[Go over all the tabs to ensure that you have everything, ask the questions again which you accidentally skipped.]

I checked the household information tab, there is no data missing:
_______________ *[date]* ______________ *[name]* _______________ *[signature of interviewer]*

IF THERE WERE MORE THAN ONE HOUSEHOLD MEMBER WHO UNDERWENT A SURGICAL PROCEDURE, FILL IN THE SECOND OR THIRD PERSONS DETAILS IN THE APPROPRIATE TABS.

C. TRANSPORTATION MEANS

The following questions will be about the availability of health facilities for you and your household members, and the transportation you are able to provide for them in need of health care.

C1.1. TRANSPORT TO PRIMARY HEALTH FACILITY:
What is the main way for you or your household members to go to a primary health facility?
___ Public transport (bus/taxi)
___ Car (motorcar or vehicle)
___ Ambulance
___ Motorcycle
___ Bicycle
___ Boat
___ Animal
___ On foot
___ Carried
___ Other – namely: ________________

[Call the village/city where this type of facility can be found for the person to be able to respond

Primary health facility: Health facility without functioning operating room]

C1.2. Travel time to primary health facility:
How long does it take you in total to get to your primary health facility if you don't have to wait for transportation (so the moment that you depart until the moment that you arrive)? (hours)

_____
_____ Don't know
[Time guideline: one person can walk 3 miles in one hour or 1 mile takes 20 minutes to walk]

C1.3. Waiting time for transport:
How long do you probably have to wait before you can leave to a primary health facility? (hours)

_____
_____ Don't know
[if participants go to health care facility by foot, waiting time is 0]

C1.4. Cost for transport:
What does it cost you to provide transportation to a primary health facility for a sick household member? (Leones, one way direction)
_____ Free (e.g. Ambulance for free, on foot, carried)
______0 – 1.000
_____ 1.001 – 2.000
_____ 2.001 – 5.000
_____ 5.001 – 10.000
_____ 10.001 – 20.000
_____ 20.001 – 50.000
_____ 50.000 – 100.000
_____ 100.000 – 250.000
_____ >250.000
_____ Don't know

C1.5. Transport money available?
Are you always able to provide these means for transport of a sick household member?
_____ Yes
_____ No
_____ Don't know

C2.1. TRANSPORT TO <u>SECONDARY</u> HEALTH FACILITY:
What is the main way for you or your household members to go to a secondary health facility?
___ Public transport (bus/taxi)
___ Car
___ Ambulance
___ Motorcycle
___ Bicycle
___ Boat
___ Animal
___ On foot
___ Carried
___ Don't know

[Name the village/city where this type of facility can be found for the person to be able to respond
Secondary health facility: Health facility with functioning operating room]

C2.2. Travel time to secondary health facility:
How long does it take you in total to get to your primary health facility if you don't have to wait for transportation (so the moment that you depart until the moment that you arrive)? (hours)

___
___ Don't know
[Time guideline: one person can walk 3 miles in one hour or 1 mile takes 20 minutes to walk]

C2.3. Waiting time for transport:
How long do you probably have to wait before you can leave to a primary health facility? (hours)

___
___ Don't know
[if participants go to health care facility by foot, waiting time is 0]

C2.4. Cost for transport:
What does it cost you to provide transportation to a secondary health facility for a sick household member? (Leones, one way direction)
_____ Free (e.g. Ambulance for free, on foot, carried)
_____ 0 – 1.000
_____ 1.001 – 2.000
_____ 2.001 – 5.000
_____ 5.001 – 10.000
_____ 10.001 – 20.000
_____ 20.001 – 50.000
_____ 50.000 – 100.000
_____ 100.000 – 250.000

_____ >250.000
_____ Don't know

C2.5. Transport money available?
Are you always able to provide these means for transport of a sick household member?
_____ Yes
_____ No
_____ Don't know

C3.1. TRANSPORT TO TERTIARY HEALTH FACILITY:
What is the main way for you or your household members to go to your tertiary health facility?
___ Public transport (bus/taxi)
___ Car
___ Ambulance
___ Motorcycle
___ Bicycle
___ Boat
___ Animal
___ On foot
___ Carried
___ Don't know

[Name the village/city where this type of facility can be found for the person to be able to respond
Tertiary health facility: *Health facility with functioning operating room and minimal one surgical specialists (Surgeons/Orthopedics/Gynecologist/Urologist)*
Tertiary health facilities per district:
- *Western area urban*
  - *Freetown*
    - *34 Military Hospital*
    - *Aberdeen Women's Centre*
    - *Princess Christian Maternity Hospital (PCMH)*

C3.2. Travel time to tertiary health facility:
How long does it take you in total to get to your primary health facility if you don't have to wait for transportation (so the moment that you depart until the moment that you arrive)? (hours)

___
___ Don't know
[Time guideline: one person can walk 3 miles in one hour or 1 mile takes 20 minutes to walk]

C3.3. Waiting time for transport:
How long do you probably have to wait before you can leave to a primary health facility? (hours)

_____
___ Don't know
[if participants go to health care facility by foot, waiting time is 0]

C3.4. Cost for transport:
What does it cost you to provide transportation to a tertiary health facility for a sick household member? (Leones, one way direction)
_____ Free (e.g. Ambulance for free, on foot, carried)
_____ 0 – 1.000
_____ 1.001 – 2.000
_____ 2.001 – 5.000
_____ 5.001 – 10.000
_____ 10.001 – 20.000
_____ 20.001 – 50.000
_____ 50.000 – 100.000
_____ 100.000 – 250.000
_____ >250.000
_____ Don't know

C3.5. Transport money available?
Are you always able to provide these means for transport of a sick household member?
_____ Yes
_____ No
_____ Don't know

D. DECEASED HOUSEHOLD MEMBERS

The following questions will be about household members that are not among us anymore. To study the quality of health services in Sierra Leone we need to ask you some questions about household members that have passed away. These questions might be disturbing to you as we need some detailed information. You might not remember everything clearly anymore, so please take your time. If you don't know the answer we would appreciate your honesty instead of guessing.

D0.1 Deaths among household members:
Did you have any household members, including babies and neonates, who passed away last 12 months?
__yes

__no

I am really sorry to hear that

D0.2 Number of deaths among household members:
How many people from your household died in the last 12 months?

____
[Ask specifically for neonates and babies.]

[FOLLOWING QUESTIONS ONLY IF THERE WAS A DECEASED HOUSEHOLD MEMBER IN THE LAST 12 MONTHS, UNDER ROW 1 THE ANSWERS FOR THE FIRST HOUSEHOLD DEATH, CONTINUE WITH THE FOLLOWING ROWS IF THERE WERE MORE HOUSEHOLD DEATHS IN THE LAST 12 MONTHS]

The following questions are about this/these deceased person(s).

D1. Age at death:
How old was the household member when she/he died?

1	2	3	4	5

Age for babies:

Time	*Name*
Before birth / in the "belly"	*X*
During Birth, alive before delivery/birth, not born alive	*0*
Within one day; born alive	*0.001*
1 day	*0.003*
2 day	*0.005*
3 day	*0.008*
4 day	*0.011*
5 day	*0.014*
6 day	*0.016*
1 week	*0.019*
2 week	*0.038*
3 week	*0.058*
4 week	*0.077*
2 months	*0,17*
3 months	*0,25*
4 months	*0,33*
5 month	*0,42*
6 months	*0,50*
7 months	*0,58*
8 months	*0,67*
9 months	*0,75*
10 months	*0,83*
11 months	*0,92*
12 months = 1 year	*1,00*

D2.1 Sex:
What was the sex of the household member?

	1	2	3	4	5
Female					
Male					
Unknown					

[IF AGE AT D1 IS HIGHER THAN 12 YRS AND D2.1 IS 'FEMALE', GO TO D2.2
IF AGE AT D1 IS HIGHER THAN 1 MONTH AND LOWER THAN 12 YRS AND D2.1 IS 'FEMALE' OR AGE AT D1 IS HIGHER THAN 1 MONTH AND D2.1 IS 'MALE', GO TO D3.1.1
IF AGE AT D1 IS LOWER THAN 1 MONTH, GO TO D3.1.3]

D2.2 Pregnant at death: *[only if age at D1 is higher than 12 yrs and D2.1 is 'Female']*
Was the household member pregnant when she died or did she die within 6 weeks after her delivery?

	1	2	3	4	5
Yes					
No					
Unknown					

[IF 'NO'' YOU CAN SKIP THE QUESTION D2.3 AND GO TO QUESTION D3.1.1 or D3.1.2]

I'm sorry to hear about your big loss. The following questions are about the cause of her death. If you don't know the answer to the question, please let me know. We appreciate your honesty.

D2.3 Deaths specifics *[only if D2.1 is answered with yes]*
Did the household member (mother) have any of the following problems less than a week before s/he died?
[multiple answers possible]

D3.1.1 Death specifics: *[only if age at D1 is higher than 1 month and lower than 12 yrs and D2.1 is 'Female' or if age at D1 is higher than 1 month and D2.1 is 'Male']*

	1	2	3	4	5
Bleeding					
Fever/infection					
Prolonged/obstructed labour; (pelvis too small / baby too big					
Convulsions					
A disease that was already there before the pregnancy, e.g. heart disease					
Too high blood pressure (hypertension)					
Complication of abortion					
Other, namely					
Unknown					

Did the household member have any of the following problems less than a week before s/he died?

	1	2	3	4	5
Injury or accident					
Wound not due to an injury					
Mass (Growth or Swelling)					
Deformity congenital					
Deformity acquired					
Abdominal distention or pain					
Other, namely…					
Unknown					

[Show empathy for the story told. Don't be judgmental in any way; let the person tell his/her story. For definitions see Question F2.1]

D3.1.2.1 Death specifics: *[only if D1 is lower than 1 month]*
Did the baby have any of the following problems before s/he died?

	1	2	3	4	5
Fever					
Difficulty breathing					
Baby never cried					
Convulsions					
Not able to breastfeed					
Not able to urinate					
Not able to defecate					
Congenital birth defect					
Other, namely					
Unknown					

D3.1.2.2 Death specifics [if deceased was newborn baby: one month old or younger]: *[Only if D3.1.2 is answered]*
[I am very sorry to hear this story. Can I ask you about the mother of this baby?]

Did his or her mother also die during the delivery or within 6 weeks after the delivery

	1	2	3	4	5
Yes					
No					
Unknown					

D3.1.3 Type of injury / accident: [Only if D3.1.1 is answered with 'Injury or accident']
After what kind of injury or accident did the problem start?

	1	2	3	4	5
Car, truck, bus crash					
Motorcycle crash					
Pedestrian, bicycle crash					
Gunshot / firearm					
Stab / slash / cut / crush					
Bite or animal attack					
Fall					
Open fire / explosion					
Hot liquid / hot object					
Other, namely…					
Unknown					

[Pick the one that best describes the injury / accident.
Pedestrian and bicycle crash: there was no motorized vehicle involved. All can be intentional or unintentional.]

D4.1 Healthcare sought: :

Did the household member go to a health facility or see a doctor/nurse for this problem in the month before she/he died?

	1	2	3	4	5
Yes					
No					
Unknown					

D4.2 Traditional Healer:
Did the household member go to a traditional healer, traditional doctor, witch doctor or bone setter for this problem?

	1	2	3	4	5
Yes					
No					
Unknown					

D4.3 Traditional healer - timing *[Only when D4.2 is answered with yes]*

	Problem 1	Problem 2	Problem 3
Before going to a health care facility			
During treatment in a health care facility			
After treatment in a health care facility			
Unknown			

D4.4 Traditional Healer: ***[only if D4.1 is 'Yes']***
How many weeks did the household member visit a traditional healer, traditional doctor, witch doctor or bone setter for this problem? (weeks)

	1	2	3	4	5
**Unknown**					

D5.0. Type of healthcare received: *[only if D4 is yes]*
What kind of treatment did the household member receive for this problem?

	1…..	2…..	3…..	4…..	5…..
None / No surgical care					
Major procedure					
Minor procedures					
Unknown					

[Major procedure: a procedure which requires regional or general anesthesia
Minor procedure: dressings, wound care, punctures, suturing and I&D including:
- *incision and drainage of an abscess*
- *circumcision (if male)*
- *normal delivery (so no caesarean birth, vacuum extraction or forceps delivery)*
- *suturing of wounds*
- *punctures*
- *conservative treatment of fractures (so no operation)*
- *tooth extractions*
- *other minor surgeries (e.g. dressings).]*

We discussed the treatment that the deceased household member received for their problem concerning the cause the death. I also like to known if s/he received other surgical treatment in the last 12 months before s/he died.

D5.1. Healthcare received in the last 12 months:
Did the household member receive any treatment for other problems in the last 12 months?

	1…..	2…..	3…..	4…..	5…..
None / No surgical care					
Major procedure					
Minor procedures					
Unknown					

[Major procedure: a procedure which requires regional or general anesthesia
Minor procedure: dressings, wound care, punctures, suturing and I&D including:
- *incision and drainage of an abscess*
- *circumcision (if male)*
- *normal delivery (so no caesarean birth, vacuum extraction or forceps delivery)*
- *suturing of wounds*
- *punctures*
- *conservative treatment of fractures (so no operation)*
- *tooth extractions*

other minor surgeries (e.g. urinary catherization, dressings).]

[if D5.0 or D5.1 indicates minor procedure has taken place]
D6.1. Minor procedures:
Which of the following minor procedures has taken place for the household member in the last 12 months?
- incision and drainage of an abscess
- circumcision (if male)
- normal delivery (so no caesarean birth, vacuum extraction or forceps delivery)
- suturing of wounds
- punctures
- conservative treatment of fractures (so no operation)
- tooth extractions
- other minor surgeries (e.g. urinary catherization, dressings).

[Fill in in table below]

**Deceased HHM**	**I&D:** Y/N	**Circumcision (male):** Y/N	**Normal Delivery:** Y/N	**Suturing:** Y/N	**Punctures** Y/N	**Conservative Treatment Fractures** Y/N	**Tooth extraction** Y/N	**Other minor surgery** Y/N	**Δ 5.3 Major Surgery** Y/N
1									
2									
3									
4									
5									

[if 'Yes' to major surgery in **5.0 or 5.1,** in D8 'tab' for each deceased household member who underwent major surgery in the same year will be opened for further specific questions*]*

D6. Reason for not having surgical care: *[Only if D4.1 is 'No' or D5.1.1 is 'No surgical care']*
What was the main reason not to go to a health facility to see a doctor/nurse or not to have an operation or dressings?
*[If the person went to a traditional healer ask why (s)he didn't go to a health care facility and mark that as the answer.
If the person was referred but did not go to the referral hospital, mark the answer why (s)he did not go here.]*

	1	2	3	4	5
No money for health care					
No (money for) transportation					
No time (person died before arrangements)					
Fear / no trust					
Not available (facility/personnel/equipment)					
Traditional healer is treating me					
I feel ashamed					
No money for the treatment					
Not fit for transport					
Not fit for surgery					
No need (condition is not surgical)					
Other, namely…					
Unknown					

D7. Location of death:
Where did the household member die?

	1	2	3	4	5
Home					
Health clinic (PHU/CHC)					
Hospital					
Traditional healing					
Somewhere else					
Unknown					

[FOLLOWING QUESTIONS ONLY IF THERE WAS A DECEASED HOUSEHOLD MEMBER IN THE LAST 12 MONTHS <u>AFTER UNDERGOING A MAJOR SURGICAL PROCEDURE IN THE SAME 12 MONTHS</u>. FOR EVERY DECEASED HOUSEHOLD MEMBER WHO UNDERWENT A SURGICAL PROCEDURE A NEW 'TAB' NEEDS TO BE OPENED

EXPLAIN:
In the previous part some questions were asked regarding household members that passed away last 12 months. We already briefly talked about major surgical procedures. In this section we would have some more specific questions about these major surgical procedures, and we would like to start with those household members who passed away last 12 months and also had an operation in the last 12 months.

\

D8. Frequency of surgical procedure:
How many times was the deceased household member operated in the last 12 months?
---- number
[if Δ1 is answered with multiple procedures, fill out the information for each of these surgical procedure in the tabs]

D9. Timing household death in relation to surgical procedure:
When (in relation to the latest surgical procedure) did the household member pass away?
___Within a week of the surgical procedure
___Within a month of the surgical procedure
___Longer than a month after the surgical procedure
___Unsure / Does not remember

D10. Type of surgical procedure:
Would you be able to tell what kind of surgical procedure was done for the household member in the last 12 months?
Dropdown list with recognizable surgical procedures
(See Q B6 for dropdown option)

D11. Localization of surgical problem:
Where in the body was the surgical problem localized?

Tell me where the surgical problem was localized

	Problem 1	Problem 2	Problem 3	Problem 4	Problem 5
Face, Head, Neck					
Chest, Breast, Back					
Abdomen					
Groin, Genitalia, Buttocks					
Extremities					
Not sure / unknown					

D12. Type of surgical problem:
Tell me what problem the HHM had that needed a surgical procedure.
[Fill in the type of problem that suits best with the undergone surgical procedure, more answers could be possible, e.g. 'Injury' and 'Wound' or 'Burn' and 'Deformity (acquired)']

	Problem 1	Problem 2	Problem 3	Problem 4	Problem 5
Injury					
Burn					
Wound					
Obstetric					
Gynecological; not pregnant					
Abdominal distention or pain					
Inability to urinate					
Mass or growth (soft)					
Mass or growth (solid)					
Deformity (congenital)					
Deformity (acquired)					
Dental					
Visual					
Unknown					

[Injury — *The person obtained a problem after an accident (e.g. broken bone after fall, or wound after motorcycle crash). Type of injury is not important here.*
Burn — *The person obtained a burn after exposure to fire or boiling water*
Wound — *Open skin; sometimes leaking blood, pus or liquid*
Obstetric — *Bleeding and/or disease around childbirth*
Gynecological — *Problem with the menstrual cycle, the womb or bleeding not related to childbirth*
Abdominal — *The person had pain or distention in the belly, or problems with eating or passing stool*
Mass or growth (soft) — *A mass or growth which feels soft and can be manipulated (e.g. lipoma, hernia)*
Mass or growth (solid) — *A mass or growth which feels firm and usually more difficult to manipulate (e.g. hydrocele, tumor)*
Deformity (congenital) — *The person is born with the problem (e.g. club feet, cleft lip)*
Deformity (acquired) — *The person had a (spontaneous – not after trauma) deformity of body or limbs later in life (e.g. chronic osteomyelitis)*
Dental — *The person had a problem with the teeth*
Visual — *The person had a problem with eyesight]*

D13. Elective or emergency surgical procedure:
Did the household member need the surgical procedure urgently due to a rapid onset of severe pain or distress?

	Surgery 1	Surgery 2	Surgery 3	Surgery 4	Surgery 5
Yes					
No					
Unsure / does not remember					

Yes

[FOLLOWING QUESTIONS ARE FOCUSSED ON THE LOCATION OR SETTING OF THE SURGICAL PROCEDURE. ALL DIFFERENT PROCEDURES THAT THE HOUSEHOLD MEMBER UNDERWENT NEED TO BE FILLED IN THE TABLE.]

D14. Location of the surgical procedure (Q I):
Was the surgical procedure done at one of the following facilities?

DROPDOWN MENU WITH THE LIST OF SURGICAL FACILITIES PREVIOUSLY IDENTIFIED
- We can categorize these in districts/cities/towns to make it easier to go through the list
- We add an OTHER option

[if 'Other' to D14, go to question D15

D15. Other surgical facility (Q I)
Where was the surgical procedure performed?

	Surgery 1	Surgery 2	Surgery 3	Surgery 4	Surgery 5
Government facility					
Private facility					
Traditional healer/doctor					
Other					
Unsure / does not remember					

D16. Give a brief explanation of the story told.

'Thank you very much for answering these questions. Let me go quickly over the survey to check everything.'

[Go over all the tabs to ensure that you have everything, ask the questions again which you accidentally skipped.]

I checked the household information tab, there is no data missing:
_______________ *[date]* _______________ *[name]* _______________ *[signature of interviewer]*

'Thank you very much for giving all this information. I have a letter with contact information for you if you would have questions for us about this survey. Can I speak to the household members who I have randomly chosen to ask them more specific questions about their health?'

[Give the letter with the (contact) information about the survey. If the household members are not available now, you should make an appointment for later on that day or the next day.] [TIME: ____ hour ____ min]

IF THERE WERE MORE THAN ONE DECEASED HOUSEHOLD MEMBER, FILL IN THE SECOND OR THIRD PERSONS DETAILS IN THE APPROPRIATE SPACES (THE TABS OR COLLUMNS WITH 2 OR 3 RESPECTIVILY.

THE FOLLOWING QUESTIONS IN SECTION E, F, G, H, I, J, K, L , ARE TO BE ASKED TO THE TWO HOUSEHOLD MEMBERS OF THE FAMILY WHO WERE RANDOMLY SELECTED FOR THE INTERVIEW.

IN THE VERSION ON THE TABLET THIS IS DISPLAYED AS EXTRA TABS, IN THE PAPER FORMAT THIS IMPLIES DOUBLE PRINTING OF THE FOLLOWING QUESTIONS. PERMANENT ATTACHEMENT OF THE FILES TOWARDS THE HOUSEHOLD INFORMATION IS NEEDED TO CONNECT THE INDIVIDUAL DATA WITH THE HOUSEHOLD AND CLUSTER DATA.

E. GENERAL INFORMATION (Survey 1I)

E1. Household list (ID) number:

_____

FIRST RESPONDENT [TIME: ____ hour ____ min]

[from the table with the questions B1 and B2]

[Check this box if this is a replaced household member] ☐

E2. Sex:

___ Male

___ Female

[If a surrogate is answering for a child, ask all the questions about the child.]

E3. Age:

[Age for babies:

<3 months	*= 0*
3- <6 months	*= 0.25*
6- <9 months	*= 0.5*
9- <12 months	*= 0.75*
12-24 months	*= 1 etc.]*

Good morning/evening. My name is _______________. I work for Masanga Medical Research Unit, who works with Statistics Sierra Leone *[show the information letter]*. We are trying to find out if there are enough doctors in this area, specifically if there are enough surgeons. A surgeon is a medical doctor who performs operations.

To find out if there are enough doctors taking care of these problems we'd like to ask you some questions. We will ask you questions about your health, such as whether you have ever had wounds, broken bones, or masses. By asking you these questions, we hope that we can help make more skilled doctors available in your village. We won't be offering medical care right now, but we can offer a referral note to those that need medical attention. We hope that the information you provide will help create improved services in the future.

This survey will take about 30 – 45 minutes. All of your responses will be confidential. You have the right to not participate in the survey, or to stop during the interview. We have an information sheet for you, and we will obtain your consent to participate. Do you have any questions at this moment?

E4. Informed consent: Would you like to participate in this survey? OR Is it okay for your son/daughter to participate in this survey?

___ Yes

___ No, what is the reason? (no time / no willingness / no reason / no seen benefit / other: explain….)

[For minors (individuals under age 18), this consent E4 is obtained from a guardian/parent. Without informed consent you cannot proceed. Make sure the person understands the purpose of this survey. If they don't want to participate, ask why and mark this.]

E.5 Minor assent: *[under 18 years of age only]* Would you like to participate in this survey?

___ Yes

___ No, what is the reason? (no time / no willingness / no reason / no seen benefit / other: explain….)

___ Surrogate consent only

[Surrogate consent only: this can be used in case of a child under 12 years of age, who is not around for the interview. The guardian/parent can answer all the questions for the child.
When the child is around, the permission to participate (assent) is asked and only with the assent of the child the interview is held together with the guardian/parent. For children over the age of 12 the guardian/parent can be around for the interview depending on the wish of the child.]

The following questions are general questions, later on I will ask more about your health.

E6. Education:

What is the highest educational level that you have achieved or are currently following?

___ None (includes nursery)

___ Primary school

___ Secondary school (junior / senior)

___ Tertiary (diploma, colleges, bachelors)

___ Graduate degree (Master degree, PhD)

E6.1 Literacy:

Are you able to read and write in any language?

[For adults and children who are currently learning how to read and write answer: 'No']

___ Yes

___ No

E7. Occupation:
What work do you do now?

___ Unemployed	*[Currently looking for jobs, retiree's, students]*
___ Home maker	*[Housewives]*
___ Domestic helpers	*[Cleaners, housekeepers, watch guards]*
___ Farmer	*[Herders, agriculture, pastoralist]*
___ Self-employed / small-business	*[Small business owners like: shops, kiosks, food traders]*
___ Government employee	*[Police officer, accountant, teachers, health care workers]*
___ Non-government employee	*[Cooperation managers, NGO-staff]*
___ Other, namely…	

E8. Tribe?
What is your tribe? *[In case of refusal to answer, ask whether the person was born in Sierra Leone]*
_____ Creole
_____ Fullah
_____ Kissi
_____ Kono
_____ Koranko
_____ Limba
_____ Loko
_____ Mandingo
_____ Mende
_____ Sherbro
_____ Susu
_____ Temne
_____ Vai
_____ Yalunka
_____ Sierra Leonean
_____ Other African
_____ Non African

E9. Length of stay in house:
How many years have you lived in this household?
_____ *[years]*

E10. Health status:
Are you generally healthy?
___ Yes
___ No
[if 'Yes' to E10. The following questions E11, E12 and E13, can be skipped]

E11. Time ill:
In total how many weeks have you been ill during the last 12 months?
_____ *[weeks]*

E12. Number of health facility visits:
How many times have you visited a clinic or hospital, or nurse / medical doctor in the last 12 months?

E13. Recovery from illness:
Have you recovered fully from all the illnesses you had?
___ Yes
___ No

E14 Diabetes
Have you ever been screened for diabetes (glucose measurement, sugar sickness)?
___ Yes
___ No
___ Unknown

E14.1 Test results for diabetes
Did the test show you have diabetes? [*only if E14 is 'Yes'*]
___ Yes
___ No
___ Unknown

E15.1 Drinking alcohol
Do you regularly drink alcohol?
___ Yes
___ No
___ Rather not say

E15.2 Amount of alcohol per week *[only if E15.1 is 'yes']*
How many units do you drink per week?
[1 packet gin or rum = 1 unit

1 normal beer = 1 unit
1 bottle palm wine (1,5L) = 6 units
1 gallon palm wine = 18 units]
___ 1 - 2
___ 3 - 5
___ 6 - 10
___ 11 - 15
___ 16 - 25
___ >25
___ Rather not say

E16. Smoking
How many tobacco do you smoke per week?
___ none
___ 1-5 tobacco per week
___ 6-20 tobacco per week
___ 21 – 40 tobacco per week
___ 41 – 60 tobacco per week
___ 61 – 100 tobacco per week
___ > 100 per week

E17. Hypertension *[only if at E3 age is higher than 18 yrs]*
Have you ever been told by a doctor or another health care worker that you have raised blood pressure?
___ Yes
___ No
___ Unknown

E17.1 Diagnose of hypertension *[only if E17 is 'yes']*
When did you know you had raised blood pressure for the first time?
___ years ago
___ I don't know

E17.2 Measurement of hypertension *[only if E17 is 'yes']*
How did you get to know your hypertension status?
___ Screened and diagnosed at a health center
___ Screened and diagnosed at a referral hospital
___ Screened at a health center and diagnosed at a referral hospital
___ Screened at a health center or a hospital and diagnosed by a private provider
___ Self diagnosed
___ Other

[private provider: private clinic, private pharmacy, traditional healer]

E18. Current medication
Do you use medication now?
___ Yes
___ No

E18.1 Medication and condition [multiple answers possible]
For which condition do you currently take medication? [only if E18 is 'Yes'?
___ High blood pressure (=Hypertension)
___ Heart problems
___ Malaria
___ Infection (not a wound)
___ Wound healing
___ Wound pain
___ Headache
___ Abdominal pain
___ Pain (other)
___ Vitamins
___ Iron supplement
___ HIV medication
___ Other, namely…

E18.2 Medication for hypertension [*only if E18.1 is 'high blood pressure']* **[multiple answers possible]**
Which medication for high blood pressure have you taken during the last twelve months?
___ Respondents are to provide answers

- Enalapril
- Amlodipine
- Nifedipine
- Atenolol
- Metoprolol
- Labetalol
- Hydrochlorthiazide

- Furosemide
- Spironolactone
- ASA = aspirin (not for pain but for pressure)

___ Tick-box
___ Other
___ Unknown
___ Unclear

E18.3 Other medication for hypertension *[only if E18.2 is 'other']*
Which other medication for high blood pressure have you taken during the last twelve months?

_____

E19. Sickle cell disease
As a child, did you have frequent bone pain and/or have you ever been tested positive for sickle cell disease?
___ Yes
___ No
___ Unknown

E19.1 Medical history other [multiple answers possible]
To the best of your knowledge, from which of the following conditions were you suffering from in the past or now?
___ Leprosy
___ Tuberculosis = TB = Dry cough
___ Heart problems
___ CVA = stroke = sudden loss of strength in limbs or difficulty with speech
___ Elephantiasis (one very swollen limb for more than a year while other side is not swollen)
___ Unknown

E20.1 EQ5D - Mobility
Do you have any problems walking now?
___ I have no problems in walking about
___ I have some problems in walking about
___ I am confined to bed

E20.2 EQ5D – Self-care
Do you have any problems washing or dressing yourself now?
___ I have no problems with self-care
___ I have some problems washing or dressing myself
___ I am unable to wash or dress myself

E20.3 EQ5D – Activities
Do you have any problems with your usual activities *(e.g. work, study, housework, family or leisure activities) now?*
___ I have no problems with performing my usual activities
___ I have some problems with performing my usual activities
___ I am unable to perform my usual activities

E20.4 EQ5D – Pain
Do you have any pain now?
___ I have no pain or discomfort
___ I have moderate pain or discomfort
___ I have extreme pain or discomfort

E20.5 EQ5D – Mental status
Are you afraid without a real reason (=anxious) or unhappy for longer period (=depressed) now?
___ I am not anxious or depressed
___ I am moderately anxious or depressed
___ I am extremely anxious or depressed

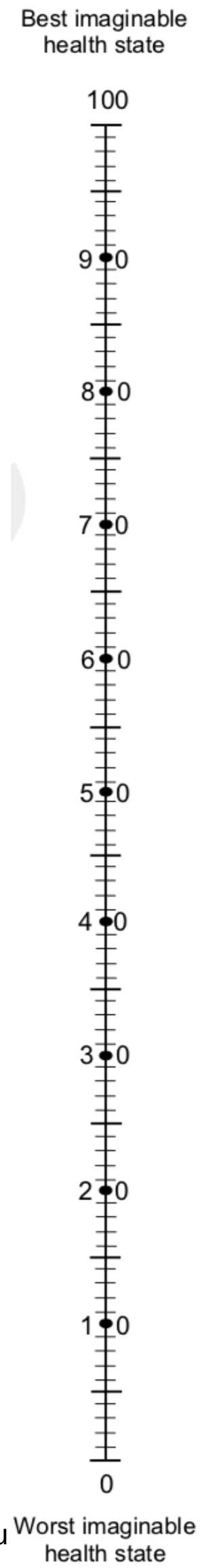

[To help people say how good or bad a health state is, 100 we have drawn a scale (rather like a thermometer) on which the best state you can imagine is marked 100 and the worst state you can imagine is marked 0. We would like you to indicate on this scale how good or bad your own health is today, in your opinion. Please do this by drawing a line from the box below to whichever point on the scale indicates how good or bad your health state is today.]

E20.6 EQ5D – Scale
Please draw on the scale.

EXPLAIN:

Earlier we talked about surgery. Some people who break a bone or have a wound, may not have an operation but still need to be seen by a doctor or stay in the surgical ward of a hospital. Since this does not include an operation, but includes surgical consultation, we call it surgical care.<br><br>Now I'm going to ask you about all the surgical problems you've had in your lifetime. We'll start with your head and move all the way down to your toes.

F. FACE / HEAD / NECK

F1.1 Face / head / neck:
Have you ever had a wound, burn, mass / goiter, deformity, problem with eating/drinking, a problem with your eyes or ears or an operation on your face, head, or neck?
___ Yes
___ No

[IF THERE WERE/ARE NO PROBLEMS WITH THIS ANATOMICAL SECTION YOU CAN CONTINUE WITH SECTION G. IF THE PERSON HAD A PROBLEM WITH THIS ANATOMICAL SECTION YOU QUESTION FIRST ON PROBLEM 1 ALL THE QUESTIONS ON THIS PAGE AND GO FURTHER IF HERE ARE MORE PROBLEMS]

F1.2 Face / head / neck location:
On what part of your head / neck / face did the problem occur?

	Problem 1	Problem 2	Problem 3
Eye			
Ear			
Nose			
Throat			
Dental			
Lips			
Mouth			
Neck			
Head			

F2.1 Face / head / neck specifics:
Tell me what problem you have had.

	Problem 1	Problem 2	Problem 3
Wound injury related			
Wound not injury related			
Burn			
Mass or growth / goiter			
Deformity congenital			
Deformity acquired			
Dental problems/abscess			

[Wound: Open skin; sometimes leaking blood, pus or liquid
Burn: Wound due to thermal, chemical or electrical cause
Deformity: An abnormal tissue arrangement or malformation
Congenital: The person is born with the problem. Think about: cleft lips, hydrocephalus etc.
Acquired: The person got the problem later in life. Think about: scars and broken bones]

F2.2 Type of injury / accident:
Did the problem start after an injury or accident? What kind of accident?
[Pick the one that best describes the injury / accident. Pedestrian and bicycle crash definition: there was no motorized vehicle involved. All can be intentional or unintentional.]

	Problem 1	Problem 2	Problem 3
No, it was not due to an injury / accident			
Car, truck, bus crash			
Motorcycle crash			
Pedestrian, bicycle crash			
Gunshot / firearm			
Stab / slash / cut / crush			
Bite or animal attack			
Fall			
Open fire / explosion			
Hot liquid / hot object			
None of the above			
Unknown			

F3.1.1 Timing:
When did this problem start?

	Problem 1	Problem 2	Problem 3
In the last month			
During the past 12 months but longer than a month ago			
Longer than 12 months ago			

F3.1.2 At this moment:
Do you have this problem now (or during the last week)?

	Problem 1	Problem 2	Problem 3
Yes			
No			

F3.2.1 Size of wound or burn: *[only if F2.1 is 'wound injury related', 'Wound not injury' related or 'burn' and F3.1.2 is 'yes']*
[Needs to be measured by the interviewer with a new ruler. Take largest diameter of largest wounds in millimeters, 10 mm = 1 cm]

	Problem 1	Problem 2	Problem 3
Size in millimeters			

F3.2.2 Start of wound: *[only if F2.1 is 'wound not due to injury/accident']*
How did your wound start?

	Problem 1	Problem 2	Problem 3
Swelling around the wound			
Swelling of the whole limb			
Redness around the wound			
Bump (nodule)			
Less than one month of pus			
More than one month of pus			
Other, namely…			
Don't know			

F3.3 Initial treatment of burn: *[only if F2.1 is 'burn']*
What was the first thing you did when you got the burn?

	Problem 1	Problem 2	Problem 3
washing with water			
covering with mud			
banana leaves			
papaya = pawpaw leaves			
papaya = pawpaw fruit			
raw eggs			
Salt			
Urine			
palm oil			
gentamycin violet			
aloe vera			
Honey			
Povidon and bandage			
none			
Other, namely…			
Don't know			

F4.1.1 Healthcare sought:
Did you go to a health facility or see a doctor/nurse for this problem?

	Problem 1	Problem 2	Problem 3
Yes			
No			

F4.1.2 Traditional Healer:
Did you go to a traditional healer, traditional doctor, witch doctor or bone setter for this problem?

	Problem 1	Problem 2	Problem 3
Yes			
No			

F4.1.3 Traditional healer - timing *[Only when F4.1.2 is answered with yes]*
[Multiple options possible]
When did you visit a traditional healer?

	Problem 1	Problem 2	Problem 3
Before visiting to a health care facility			
During treatment in a health care facility			
After visiting in a health care facility			
Unknown			

F4.1.4 Traditional Healer: *[only if F4.1.2 is 'Yes']*
For how long did you visit a traditional healer, traditional doctor, witch doctor or bone setter for this problem? (months)

	Problem 1	Problem 2	Problem 3
Months			
Don't know			

F5.1.1 Type of healthcare received: *[Only when 'yes' to F4.1.1]*
What kind of treatment did you receive?

	Problem 1	Problem 2	Problem 3
None / No surgical care			
Major procedure			
Minor procedures			
Don't know			

[Major procedure: a procedure which requires regional or general anesthesia
Minor procedure: dressings, wound care, punctures, suturing and I&D]

F5.1.2 Type of treatment received wounds: *[only if G2.1 is 'wound injury related', 'Wound not injury' related or 'burn' and G3.1.2 is 'yes']*
What kind of treatment did you have for your wound or burn?

	Problem 1	Problem 2	Problem 3
Traditional medicine by mouth (to eat or drink or swallow)			
traditional medicine such as leaves or ointment			
modern medicine tablets (any oral drugs) self bought from a pharmacy or drug peddler			
modern medicine ointment / crème self bought from a pharmacy or drug peddler			
Honey			
gentamycin violet (bright purple)			
X-ray			
Dressing at PHU			
Dressing at hospital			
Surgical debridement / operation by traditional healer			
Surgical debridement / operation by PHU or hospital			
Skin grafting			
None			
Other, namely...			
Don't know			

F5.1.3 Coverage of wound by skin graft: *[only if F5.1.2 is 'Skin graft]*
Was your wound covered completely by the skin graft when you left the health facility?
___ Yes
___ No

[a skin graft is a surgery where healthy skin is taken (almost always from the upper leg) to cover a wound or bur)]

F5.1.4 Location skin graft: *[only if F5.1.2 is 'Skin graft]*
Where was your skin graft done?
____ (name of health facility where skin graft was done)
DROP DOWN MENU TO BE ADDED

F6.1. Reason for not having surgical care: *[Only if F5.1.1 is 'none / no surgical care' or F4.1.1 is 'no']*
What was the main reason not to go to a health facility to see a doctor/nurse or not to have an operation or dressings?
[If the person went to a traditional healer ask why (s)he didn't go to a health care facility and mark that as the answer.
If the person was referred but did not go to the referral hospital, mark the answer why (s)he did not go here.]

	Problem 1	Problem 2	Problem 3
No money for health care			
No (money for) transportation			
No time			
Fear / no trust			
Not available (facility/personnel/equipment)			
Traditional healer is treating me			
I feel ashamed			
I am currently saving money for a treatment			
Because it was during the Ebola period			
No need (condition is not surgical)			
Other, namely…			
Unknown			

F7.1. Disability:
Does this problem still impact your daily life?

	Problem 1	Problem 2	Problem 3
The condition is not disabling			
I feel ashamed			
I'm not able to work like I used to			
I need help with transportation			
I need physical help with daily living			

[this question is not meant in a financial way but rather if a patient needs physical help with transportation (wheelchair or walking stick or can not walk small distances) or daily living (such as getting out of bed, brushing teeth, eating]
[Disability: a physical problem that impacts your life, or makes it difficult to carry out your daily activities.]

[ASK IF THE PERSON HAD ANOTHER PROBLEM REGARDING THIS ANATOMICAL LOCATION. IF SO, GO TO THE FOLLOWING ROW, PROBLEM 2 OR 3, TO REGISTER THIS PROBLEM. IF HERE ARE NO OTHER PROBLEMS REGARDING TO THIS LOCATION YOU CAN GO TO THE FOLLOWING ANATOMICAL LOCATION. IN CASE OF MORE THAN 3 PROBLEMS, MARK THE ONES WHICH ARE MOST RESENT AND MOST RELEVANT FOR THE REPONDENT]

G. CHEST / BREAST (TAB: Survey Part II)

G1 Chest / breast:
Have you ever had a wound, burn, breast mass, deformity, or an operation on your chest (including heart or lungs) or breast?
___ Yes
___ No

[If there were/are no problems with this anatomical section you can continue with the following. If the person had a problem with this anatomical section you question first on problem 1 all the questions on this page and go further if here are more problems]

G2.1 Chest / breast specifics:
Tell me what problem you have had.

	Problem 1	Problem 2	Problem 3
Wound injury related			
Wound not injury related			
Burn			
Breast mass or growth			
Deformity congenital			
Deformity acquired			
Heart operation			
Lung/chest drainage			

[Wound: Open skin; sometimes leaking blood, pus or liquid
Burn: Wound due to thermal, chemical or electrical cause
Deformity: An abnormal tissue arrangement, malformation
Congenital: The person is born with the problem. Example: heart malformation
Acquired: The person got the problem later in life]

G2.2 Type of injury / accident:
Did the problem start after an injury or accident? What kind of accident?
[Pick the one that best describes the injury / accident. Pedestrian and bicycle crash definition: there was no motorized vehicle involved. All can be intentional or unintentional.]

	Problem 1	Problem 2	Problem 3
No, it was not due to an injury / accident			
Car, truck, bus crash			
Motorcycle crash			
Pedestrian, bicycle crash			
Gunshot / firearm			
Stab / slash / cut / crush			
Bite or animal attack			
Fall			
Open fire / explosion			
Hot liquid / hot object			
None of the above			
Unknown			

[Pedestrian and bicycle crash: there was no motorized vehicle involved. All can be intentional or unintentional.]

G3.1.1 Timing:
When did this problem start?

	Problem 1	Problem 2	Problem 3
In the last month			
During the past 12 months but longer than a month ago			
Longer than 12 months ago			

G3.1.2 At this moment:

Do you have this problem now (or during the last week)?

	Problem 1	Problem 2	Problem 3
Yes			
No			

G3.2.1 Size of wound or burn: *[only if G2.1 is 'wound injury related', 'Wound not injury' related or 'burn' and G3.1.2 is 'yes']*
[Needs to be measured by the interviewer with a new ruler. Take largest diameter of largest wounds in millimeters, 10 mm = 1 cm]

	Problem 1	Problem 2	Problem 3
Size in millimeters			

G3.2.2 Start of wound:
How did your wound start? *[only if wound not due to injury/accident]*

	Problem 1	Problem 2	Problem 3
Swelling around the wound			
Swelling of the whole limb			
Redness around the wound			
Bump (nodule)			
Less than one month of pus			
More than one month of pus			
Other, namely…			
Don't know			

G3.3.1 Initial treatment of burn: *[only if G2.1 is 'Burn']*
What was the first thing you did when you got the burn?

	Problem 1	Problem 2	Problem 3
washing with water			
covering with mud			
banana leaves			
papaya = pawpaw leaves			
papaya = pawpaw fruit			
raw eggs			
Salt			
Urine			
palm oil			
gentamycin violet			
aloe vera			
None			
Other, namely...			
Don't know			

G4.1.1 Healthcare sought:
Did you go to a health facility or see a doctor/nurse for this problem?

	Problem 1	Problem 2	Problem 3
Yes			
No			

G4.1.2 Traditional Healer:
Did you go to a traditional healer, traditional doctor, witch doctor or bone setter for this problem?

	Problem 1	Problem 2	Problem 3
Yes			
No			

G4.1.3 Traditional healer - timing *[Only when G4.1.2 is answered with yes]*
When did you visit a traditional healer?

	Problem 1	Problem 2	Problem 3
Before going to a health care facility			
During treatment in a health care facility			
After visiting in a health care facility			
Unknown			

G4.1.4 Traditional Healer: *[only if F4.1.2 is 'Yes']*
How long did you visit a traditional healer, traditional doctor, witch doctor or bone setter for this problem? (months)

	Problem 1	Problem 2	Problem 3
Months			
Don't know			

G5.1.1 Type of healthcare received: *[Only when 'Yes' to G4.1.1]*
What kind of treatment did you receive?

	Problem 1	Problem 2	Problem 3
None / No surgical care			
Major procedure			
Minor procedures			
Don't know			

[Major procedure: a procedure which requires regional or general anesthesia
Minor procedure: dressings, wound care, punctures, suturing and I&D]

G5.1.2 Type of treatment received wounds: *[only if G2.1 is 'wound injury related', 'Wound not injury' related or 'burn' and G3.1.2 is 'yes']*
What kind of treatment did you have for your wound or burn?

	Problem 1	Problem 2	Problem 3
Traditional medicine by mouth (to eat or drink or swallow)			
traditional medicine such as leaves or ointment			
modern medicine tablets (any oral drugs) self bought from a pharmacy or drug peddler			
modern medicine ointment / crème self bought from a pharmacy or drug peddler			
Honey			
gentamycin violet (bright purple)			
X-ray			
Dressing at PHU			
Dressing at hospital			
Surgical debridement / operation by traditional healer			
Surgical debridement / operation by PHU or hospital			
Skin grafting			
None			
Other, namely…			
Don't know			

G5.1.3: Coverage of wound by skin graft *[only if G5.1.2 is 'Skin graft]*
Was your wound covered completely by the skin graft when you left the health facility?
___ Yes
___ No

[a skin graft is a surgery where healthy skin is taken (almost always from the upper leg) to cover a wound or bur)]

G5.1.4: Location skin graft *[only if G5.1.2 is 'Skin graft]*
Where was your skin graft done?
____ (name of health facility where skin graft was done)
DROPDOWN MENU

G6.1. Reason for not having surgical care: *[Only if G5.1.1 is 'none / no surgical care' or G4.1.1 is 'no']*
What was the main reason not to go to a health facility to see a doctor/nurse or not to have an operation or dressings?
[If the person went to a traditional healer ask why (s)he didn't go to a health care facility and mark that as the answer.
If the person was referred but did not go to the referral hospital, mark the answer why (s)he did not go here.]

	Problem 1	Problem 2	Problem 3
No money for health care			
No (money for) transportation			
No time			
Fear / no trust			
Not available (facility/personnel/equipment)			
Traditional healer is treating me			
I feel ashamed			
I am currently saving money for a treatment			
Because it was during the Ebola period			
No need (condition is not surgical)			
Other, namely…			
Unknown			

G7.1. Disability:
Does this problem still impact your daily life?

	Problem 1	Problem 2	Problem 3
The condition is not disabling			
I feel ashamed			
I'm not able to work like I used to			
I need help with transportation			
I need physical help with daily living			

[This question is not meant in a financial way but rather if a patient needs physical help with transportation (wheelchair or walking stick or can not walk small distances) or daily living (such as getting out of bed, brushing teeth, eating.
Disability: a physical problem that impacts your life, or makes it difficult to carry out your daily activities.]

[ASK IF THE PERSON HAD ANOTHER PROBLEM REGARDING THIS ANATOMICAL LOCATION. IF SO, GO TO THE FOLLOWING ROW, PROBLEM 2 OR 3, TO REGISTER THIS PROBLEM. IF HERE ARE NO OTHER PROBLEMS REGARDING TO THIS LOCATION YOU CAN GO TO THE FOLLOWING ANATOMICAL LOCATION. IN CASE OF MORE THAN 3 PROBLEMS, MARK THE ONES WHICH ARE MOST RESENT AND MOST RELEVANT FOR THE REPONDENT]

H. BACK

H1. Back:
Have you ever had a wound, burn, mass, deformity, or an operation on your back?
___ Yes
___ No

[If there were/are no problems with this anatomical section you can continue with the following. If the person had a problem with this anatomical section you question first on problem 1 all the questions on this page and go further if here are more problems]

H2.1. Back specifics:
Tell me what problem you have had.

	Problem 1	Problem 2	Problem 3
Wound injury related			
Wound not injury related			
Burn			
Mass or growth			
Deformity congenital			
Deformity acquired			

[Wound: Open skin; sometimes leaking blood, pus or liquid
Burn: Wound due to thermal, chemical or electrical cause
Deformity: An abnormal tissue arrangement, malformation
Congenital: The person is born with the problem.
Acquired: The person got the problem later in life]

H2.1.1 Type of injury / accident:
Did the problem started after an injury or accident? What kind of accident?
[Pick the one that best describes the injury / accident. Pedestrian and bicycle crash definition: there was no motorized vehicle involved. All can be intentional or unintentional.]

	Problem 1	Problem 2	Problem 3
No, it was not due to an injury / accident			
Car, truck, bus crash			
Motorcycle crash			
Pedestrian, bicycle crash			
Gunshot / firearm			
Stab / slash / cut / crush			
Bite or animal attack			
Fall			
Open fire / explosion			
Hot liquid / hot object			
None of the above			
Unknown			

F3.1. Timing:
When did this problem start?

	Problem 1	Problem 2	Problem 3
In the last month			
During the past 12 months but longer than a month ago			
Longer than 12 months ago			

H3.1.1 At this moment:
Do you have this problem now (or during the last week)?

	Problem 1	Problem 2	Problem 3
Yes			
No			

H3.2.1 Size: How large is your wound or burn? [only if wound or burn currently present]
[Needs to be measured by the interviewer with a new ruler. Take largest diameter of largest wounds in millimeters, 10 mm = 1 cm]

	Problem 1	Problem 2	Problem 3
Size in millimeters			

H3.2.2 Start of wound:
How did your wound start?
[only if wound not due to injury/accident]

	Problem 1	Problem 2	Problem 3
Swelling around the wound			
Swelling of the whole limb			
Redness around the wound			
Bump (nodule)			
Less than one month of pus			
More than one month of pus			
Other, namely…			
Don't know			

H3.3.1 Initial treatment of burn: *[only if H2.1.1 is 'Burn']*
What was the first thing you did when you got the burn?

	Problem 1	Problem 2	Problem 3
washing with water			
covering with mud			
banana leaves			
papaya = pawpaw leaves			
papaya = pawpaw fruit			
raw eggs			
Salt			
Urine			
palm oil			
gentamycin violet			
aloe vera			
none			
Other, namely...			
Don't know			

H4.1. Healthcare sought:
Did you go to a health facility or see a doctor/nurse for this problem?

	Problem 1	Problem 2	Problem 3
Yes			
No			

H4.1.1. Traditional Healer:
Did you go to a traditional healer, traditional doctor, witch doctor or bone setter for this problem?

	Problem 1	Problem 2	Problem 3
Yes			
No			

H4.1.2 Traditional healer - timing *[Only when H4.1.1 is answered with yes]*
When did you visit a traditional healer?

	Problem 1	Problem 2	Problem 3
Before going to a health care facility			
During treatment in a health care facility			
After treatment in a health care facility			
Unknown			

H4.1.3. Traditional Healer: ***[only if F4.1.1 is 'Yes' to one or more of the problems]***
For how long did you visit a traditional healer, traditional doctor, witch doctor or bone setter for this problem? (months)

	Problem 1	Problem 2	Problem 3
Months			
Don't know			

H5.1. Type of healthcare received: *[Only when 'Yes' to H4.1]*
What kind of treatment did you receive?

	Problem 1	Problem 2	Problem 3
None / No surgical care			
Major procedure			
Minor procedures			
Don't know			

[Major procedure: a procedure which requires regional or general anesthesia
Minor procedure: dressings, wound care, punctures, suturing and I&D]

H5.1.1. Type of treatment received wounds: *[only if wound or burn present]*
What kind of treatment did you have for your wound or burn?

	Problem 1	Problem 2	Problem 3
Traditional medicine by mouth (to eat or drink or swallow)			

traditional medicine such as leaves or ointment			
modern medicine tablets (any oral drugs) self bought from a pharmacy or drug peddler			
modern medicine ointment / crème self bought from a pharmacy or drug peddler			
Honey			
gentamycin violet (bright purple)			
X-ray			
Dressing at PHU			
Dressing at hospital			
Surgical debridement / operation by traditional healer			
Surgical debridement / operation by PHU or hospital			
Skin grafting			
None			
Other, namely…			
Don't know			

H5.1.3: Coverage of wound by skin graft *[only if H5.1.2 is 'Skin graft]*
Was your wound covered completely by the skin graft when you left the health facility?
___ Yes
___ No

[a skin graft is a surgery where healthy skin is taken (almost always from the upper leg) to cover a wound or bur)]

H5.1.4: Location skin graft *[only if H5.1.2 is 'Skin graft]*
Where was your skin graft done?
____ (name of health facility where skin graft was done)
DROPDOWN MENU

H6.1. Reason for not having surgical care: *[Only when 'none / no surgical care' to H5.1 or 'no' to H4.1]*
What was the main reason not to go to a health facility to see a doctor/nurse or not to have an operation or dressings?
[If the person went to a traditional healer ask why (s)he didn't go to a health care facility and mark that as the answer. If the person was referred but did not go to the referral hospital, mark the answer why (s)he did not go here.]

	Problem 1	Problem 2	Problem 3
No money for health care			
No (money for) transportation			
No time			
Fear / no trust			
Not available (facility/personnel/equipment)			
Traditional healer is treating me			
I feel ashamed			
I am currently saving money for a treatment			
Because it was during the Ebola period			
No need (condition is not surgical)			
Other, namely…			
Unknown			

H7.1. Disability:
Does this problem still impact your daily life?

	Problem 1	Problem 2	Problem 3
The condition is not disabling			
I feel ashamed			
I'm not able to work like I used to			
I need help with transportation			
I need physical help with daily living			

[this question is not meant in a financial way but rather if a patient needs physical help with transportation (wheelchair or walking stick or can not walk small distances) or daily living (such as getting out of bed, brushing teeth, eating]
[Disability: a physical problem that impacts your life, or makes it difficult to carry out your daily activities.]

[ASK IF THE PERSON HAD ANOTHER PROBLEM REGARDING THIS ANATOMICAL LOCATION. IF SO, GO TO THE FOLLOWING ROW, PROBLEM 2 OR 3, TO REGISTER THIS PROBLEM. IF HERE ARE NO OTHER PROBLEMS REGARDING TO THIS LOCATION YOU CAN GO TO THE FOLLOWING ANATOMICAL LOCATION. IN CASE OF MORE THAN 3 PROBLEMS, MARK THE ONES WHICH ARE MOST RESENT AND MOST RELEVANT FOR THE REPONDENT]

I. ABDOMEN

I1. Abdomen:
Have you ever had a wound, burn, mass, deformity, inability to urinate, bleeding from your bottom, abdominal delivery, or an operation on your abdomen?
___ Yes
___ No
[If there were/are no problems with this anatomical section you can continue with the following. If the person had a problem with this anatomical section you question first on problem 1 all the questions on this page and go further if here are more problems]

I2.1. Abdomen specifics:
Tell me what problem you have had.

	Problem 1	Problem 2	Problem 3
Wound injury related			
Wound not injury related			
Burn			
Mass or growth (solid)			
Mass or growth (soft reducible)			
Deformity congenital			
Deformity acquired			
Abdominal distention or pain			
Inability to urinate			
Bleeding (per rectum)			
Bleeding (per penis or vagina)			
CS / operation during childbirth			

I2.1.1 Type of injury / accident:
Did the problem started after an injury or accident? What kind of accident?
[Pick the one that best describes the injury / accident. Pedestrian and bicycle crash definition: there was no motorized vehicle involved. All can be intentional or unintentional.]

	Problem 1	Problem 2	Problem 3
No, it was not due to an injury / accident			
Car, truck, bus crash			
Motorcycle crash			
Pedestrian, bicycle crash			
Gunshot / firearm			
Stab / slash / cut / crush			
Bite or animal attack			
Fall			
Open fire / explosion			
Hot liquid / hot object			
None of the above			
Unknown			

F3.1. Timing:
When did this problem start?

	Problem 1	Problem 2	Problem 3
In the last month			
During the past 12 months but longer than a month ago			
Longer than 12 months ago			

I3.1.1 At this moment:
Do you have this problem now (or during the last week)?

	Problem 1	Problem 2	Problem 3
Yes			
No			

I3.2.1 Size: How large is your wound or burn? [only if wound or burn currently present]
[Needs to be measured by the interviewer with a new ruler. Take largest diameter of largest wounds in millimeters, 10 mm = 1 cm]

	Problem 1	Problem 2	Problem 3
Size in millimeters			

I3.2.2 Start of wound:
How did your wound start? *[only if wound not due to injury/accident]*

	Problem 1	Problem 2	Problem 3
Swelling around the wound			
Swelling of the whole limb			
Redness around the wound			
Bump (nodule)			
Less than one month of pus			
More than one month of pus			
Other, namely…			
Don't know			

I3.3.1 Initial treatment of burn: *[only if I2.1 is 'Burn']*

What was the first thing you did when you got the burn?

	Problem 1	Problem 2	Problem 3
washing with water			
covering with mud			
banana leaves			
papaya = pawpaw leaves			
papaya = pawpaw fruit			
raw eggs			
Salt			
Urine			
palm oil			
gentamycin violet			
aloe vera			
none			
Other, namely…			
Don't know			

I4.1. Healthcare sought:
Did you go to a health facility or see a doctor/nurse for this problem?

	Problem 1	Problem 2	Problem 3
Yes			
No			

I4.1.1. Traditional Healer:
Did you go to a traditional healer, traditional doctor, witch doctor or bone setter for this problem?

	Problem 1	Problem 2	Problem 3
Yes			
No			

I4.1.2 Traditional healer - timing *[Only when I4.1.1 is answered with yes]*
When did you visit a traditional healer?

	Problem 1	Problem 2	Problem 3
Before going to a health care facility			
During treatment in a health care facility			
After treatment in a health care facility			
Unknown			

I4.1.3 Traditional Healer: ***[only if F4.1.1 is 'Yes' to one or more of the problems]***
How long did you visit a traditional healer, traditional doctor, witch doctor or bone setter for this problem? (months)

	Problem 1	Problem 2	Problem 3
Months			
Don't know			

I5.1. Type of healthcare received: *[Only when 'Yes' to I4.1]*
What kind of treatment did you receive?

	Problem 1	Problem 2	Problem 3
None / No surgical care			
Major procedure			
Minor procedures			
Cesarean section			
Don't know			

[Major procedure: a procedure which requires regional or general anesthesia
Minor procedure: dressings, wound care, punctures, suturing and I&D
Cesarean section: Abdominal delivery. The baby is born with an abdominal operation]

I5.1.1. Type of treatment received wounds: *[only if wound or burn present]*
What kind of treatment did you have for your wound or burn?

	Problem 1	Problem 2	Problem 3
Traditional medicine by mouth (to eat or drink or swallow)			
traditional medicine such as leaves or ointment			
modern medicine tablets (any oral drugs) self bought from a pharmacy or drug peddler			
modern medicine ointment / crème self bought from a pharmacy or drug peddler			
Honey			
gentamycin violet (bright purple)			
X-ray			
Dressing at PHU			
Dressing at hospital			
Surgical debridement / operation by traditional healer			
Surgical debridement / operation by PHU or hospital			
Skin grafting			
None			

Other, namely…			
Don't know			

I5.1.3: Coverage of wound by skin graft *[only if I5.1.2 is 'Skin graft]*
Was your wound covered completely by the skin graft when you left the health facility?
___ Yes
___ No

[a skin graft is a surgery where healthy skin is taken (almost always from the upper leg) to cover a wound or bur)]

I5.1.4: Location skin graft *[only if I5.1.2 is 'Skin graft]*
Where was your skin graft done?
____ (name of health facility where skin graft was done)
DROPDOWN MENU

I6.1. Reason for not having surgical care: *[Only when 'none / no surgical care' to i5.1 or 'no' to I4.1]*
What was the main reason not to go to a health facility to see a doctor/nurse or not to have an operation or dressings?
[If the person went to a traditional healer ask why (s)he didn't go to a health care facility and mark that as the answer. If the person was referred but did not go to the referral hospital, mark the answer why (s)he did not go here.]

	Problem 1	Problem 2	Problem 3
No money for health care			
No (money for) transportation			
No time			
Fear / no trust			
Not available (facility/personnel/equipment)			
Traditional healer is treating me			
I feel ashamed			
I am currently saving money for a treatment			
Because it was during the Ebola period			
No need (condition is not surgical)			
Other, namely…			
Unknown			

I7.1. Disability:
Does this problem still impact your daily life?

	Problem 1	Problem 2	Problem 3
The condition is not disabling			
I feel ashamed			
I'm not able to work like I used to			
I need help with transportation			
I need physical help with daily living			

[this question is not meant in a financial way but rather if a patient needs physical help with transportation (wheelchair or walking stick or can not walk small distances) or daily living (such as getting out of bed, brushing teeth, eating]
[Disability: a physical problem that impacts your life, or makes it difficult to carry out your daily activities.]

[ASK IF THE PERSON HAD ANOTHER PROBLEM REGARDING THIS ANATOMICAL LOCATION. IF SO, GO TO THE FOLLOWING ROW, PROBLEM 2 OR 3, TO REGISTER THIS PROBLEM. IF HERE ARE NO OTHER PROBLEMS REGARDING TO THIS LOCATION YOU CAN GO TO THE FOLLOWING ANATOMICAL LOCATION. IN CASE OF MORE THAN 3 PROBLEMS, MARK THE ONES WHICH ARE MOST RESENT AND MOST RELEVANT FOR THE REPONDENT]

J. GROIN / GENITALIA / BUTTOCKS

J1. Groin / genitalia / buttocks:
Have you ever had a wound, burn, mass, deformity, leaking of urine or feces, bleeding from your bottom, bleeding from your penis, or an operation on your groin, genitalia or buttocks?
___ Yes
___ No

J2.1.1 Groin / genitalia specifics:
Tell me what problem you have had.

	Problem 1	Problem 2	Problem 3
Wound injury related			
Wound not injury related			
Burn			
Mass or growth (solid)			
Mass or growth (soft reducible)			
Hernia			
Deformity congenital			
Deformity acquired			
Leaking of urine or feces			
Bleeding (per rectum)			
Bleeding (per penis or vagina)			

[Mass or growth (solid): Testicular cancer or hydrocele/cystocele
Mass or growth (soft reducible): Groin hernia
Deformity: An abnormal tissue arrangement, malformation
Congenital: The person is born with the problem. Think about: born without anus, hypospadias etc.
Acquired: The person got the problem later in life]

J2.1.2 Localization *[only if J.2.1.1 is 'Mass or growth (solid)' or 'mass or growth (soft reducible)'*
Where do you have this problem?

	Problem 1	Problem 2	Problem 3
On the left side			
On the right side			
Bilateral			

[bilateral: both sides of the body]

J2.1.3 Type of injury / accident:
Did the problem started after an injury or accident? What kind of accident?
[Pick the one that best describes the injury / accident. Pedestrian and bicycle crash definition: there was no motorized vehicle involved. All can be intentional or unintentional.]

	Problem 1	Problem 2	Problem 3
No, it was not due to an injury / accident			
Car, truck, bus crash			
Motorcycle crash			
Pedestrian, bicycle crash			
Gunshot / firearm			
Stab / slash / cut / crush			
Bite or animal attack			
Fall			
Open fire / explosion			
Hot liquid / hot object			
None of the above			
Unknown			
Other, namely…			
Don't know			

J3.1. Timing:
When did this problem start?

	Problem 1	Problem 2	Problem 3
In the last month			
During the past 12 months but longer than a month ago			
Longer than 12 months ago			

J3.1.1 At his moment:
Do you have this problem now (or during the last week)?

	Problem 1	Problem 2	Problem 3
Yes			
No			

J3.1.2 Pain at this moment: *[only when 'Mass or growth (solid)' and 'Mass or growth (soft or reducible)' to J2.1 and J3.1.1 is 'yes']*
Estimate the worst pain you felt due to this problem now (or during the last week)?

	Problem 1	Problem 2	Problem 3
No pain			
Pain present but can easily be ignored			
Pain present, cannot be ignored but does not interfere with daily activities			
Pain present, cannot be ignored and interferes with concentration and activities			
Pain present, interferes with most activities			
Pain present, necessitates bed rest			
Pain present, prompt medical advice sought			

J3.2.1 Size: How large is your wound or burn? [only if wound or burn currently present]
[Needs to be measured by the interviewer with a new ruler. Take largest diameter of largest wounds in millimeters, 10 mm = 1 cm]

	Problem 1	Problem 2	Problem 3
Size in millimeters			

J3.2.2 Start of wound: *[only if wound not due to injury/accident AND J3.1.1 is 'Yes']*
How did your wound start?

	Problem 1	Problem 2	Problem 3
Swelling around the wound			
Swelling of the whole limb			

Redness around the wound			
Bump (nodule)			
Less than one month of pus			
More than one month of pus			
Other, namely…			
Don't know			

J3.3.1 Initial treatment of burn: *[only if J2.1.1 is 'Burn']*
What was the first thing you did when you got the burn?

	Problem 1	Problem 2	Problem 3
washing with water			
covering with mud			
banana leaves			
papaya = pawpaw leaves			
papaya = pawpaw fruit			
raw eggs			
salt			
urine			
palm oil			
gentamycin violet			
aloe vera			
none			
Other, namely…			
Don't know			

J4.1. Healthcare sought:
Did you go to a health facility or see a doctor/nurse for this problem?

	Problem 1	Problem 2	Problem 3
Yes			
No			

J4.1.1 Traditional Healer:
Did you go to a traditional healer, traditional doctor, witch doctor or bone setter for this problem?

	Problem 1	Problem 2	Problem 3
Yes			
No			

J4.1.2 Traditional healer - timing *[Only when J4.1.1 is answered with yes]*
When did you visit a traditional healer?

	Problem 1	Problem 2	Problem 3
Before going to a health care facility			
During treatment in a health care facility			
After treatment in a health care facility			
Unknown			

J4.1.3 Traditional Healer: ***[only if F4.1.1 is 'Yes' to one or more of the problems]***
How long did you visit a traditional healer, traditional doctor, witch doctor or bone setter for this problem? (months)

	Problem 1	Problem 2	Problem 3
Months			
Don't know			

J5.1. Type of healthcare received: *[Only when 'Yes' to J4.1]*
What kind of treatment did you receive?

	Problem 1	Problem 2	Problem 3
None / No surgical care			
Major procedure			
Minor procedures			
Don't know			

[Major procedure: a procedure which requires regional or general anesthesia
Minor procedure: dressings, wound care, punctures, suturing and I&D]

J5.1.1. Type of treatment received wounds: *[only if wound or burn present]*
What kind of treatment did you have for your wound or burn?

	Problem 1	Problem 2	Problem 3
Traditional medicine by mouth (to eat or drink or swallow)			
traditional medicine such as leaves or ointment			
modern medicine tablets (any oral drugs) self bought from a pharmacy or drug peddler			
modern medicine ointment / crème self bought from a pharmacy or drug peddler			

Honey			
gentamycin violet (bright purple)			
X-ray			
Dressing at PHU			
Dressing at hospital			
Surgical debridement / operation by traditional healer			
Surgical debridement / operation by PHU or hospital			
Skin grafting			
None			
Other, namely…			
Don't know			

J5.1.3: Coverage of wound by skin graft *[only if J5.1.2 is 'Skin graft]*
Was your wound covered completely by the skin graft when you left the health facility?
___ Yes
___ No

[a skin graft is a surgery where healthy skin is taken (almost always from the upper leg) to cover a wound or bur)]

J5.1.4: Location skin graft *[only if J5.1.2 is 'Skin graft]*
Where was your skin graft done?
____ (name of health facility where skin graft was done)
DROPDOWN MENU

J6.1. Reason for not having surgical care: *[Only when 'none / no surgical care' to J5.1 or 'no' to J4.1]*
What was the main reason not to go to a health facility to see a doctor/nurse or not to have an operation or dressings?
[If the person went to a traditional healer ask why (s)he didn't go to a health care facility and mark that as the answer. If the person was referred but did not go to the referral hospital, mark the answer why (s)he did not go here.]

	Problem 1	Problem 2	Problem 3
No money for health care			
No (money for) transportation			
No time			
Fear / no trust			
Not available (facility/personnel/equipment)			
Traditional healer is treating me			
I feel ashamed			
I am currently saving money for a treatment			
Because it was during the Ebola period			
No need (condition is not surgical)			
Other, namely…			
Unknown			

J7.1. Disability:
Does this problem still impact your daily life?

	Problem 1	Problem 2	Problem 3
The condition is not disabling			
I feel ashamed			
I'm not able to work like I used to			
I need help with transportation			
I need physical help with daily living			

[this question is not meant in a financial way but rather if a patient needs physical help with transportation (wheelchair or walking stick or can not walk small distances) or daily living (such as getting out of bed, brushing teeth, eating]
[Disability: a physical problem that impacts your life, or makes it difficult to carry out your daily activities.]

J8.1.1 Treatment – possible treatment *[only when 'Mass or growth (solid)', 'Mass or growth (soft or reducible)' or 'Hernia' to J2.1]*
Have you ever been operated for a groin hernia?

	Problem 1	Problem 2	Problem 3
Yes			
No			

J8.1.2 Treatment – possible treatment *[only when J3.1.1 is 'Yes' and J8.1.1 is 'No']*
The mass or growth in the groin might be a groin hernia. If this is a groin hernia, are you aware of the fact that you can be operated for it in the hospital?

	Problem 1	Problem 2	Problem 3
Yes			
No			

J8.1.1 Treatment – amount of treatments *[only if J8.1.1 is 'Yes']*
How many times have you been operated for a groin hernia?

	Problem 1	Problem 2	Problem 3
Unknown			

J8.1.4 Treatment – Location of the surgical procedure *[only if J8.1.1 is 'yes']*
Was the surgical procedure done at one of the following facilities?

DROPDOWN MENU WITH THE LIST OF SURGICAL FACILITIES PREVIOUSLY IDENTIFIED
- We can categorize these in districts/cities/towns to make it easier to go through the list
- We add an OTHER option
- If OTHER, we specify
    - Government (I got feedback from a tutor from Ghana that people normally know the distinction between gov & private)
    - Private
    - It was suggested to remove traditional healer as it is earlier proposed that they do not perform major surgery
- If OTHER, I propose not to look for the village/location. After all, we are doing this to potentially describe a phenomenon, not to witch-hunt unregulated surgical facilities. This makes life a lot easier in terms of the questionnaire, and we have less chance to enter a sensitive topic.
- If OTHER, we could still determine if the operation was done at an operation theatre?

[ASK IF THE PERSON HAD ANOTHER PROBLEM REGARDING THIS ANATOMICAL LOCATION. IF SO, GO TO THE FOLLOWING ROW, PROBLEM 2 OR 3, TO REGISTER THIS PROBLEM. IF HERE ARE NO OTHER PROBLEMS REGARDING TO THIS LOCATION YOU CAN GO TO THE FOLLOWING ANATOMICAL LOCATION. IN CASE OF MORE THAN 3 PROBLEMS, MARK THE ONES WHICH ARE MOST RESENT AND MOST RELEVANT FOR THE REPONDENT. IF ONE THE PROBLEMS IS POSSIBLE AN GROIN HERNIA (SO WHEN MASS OR GROWTH (SOLID)' OR 'MASS (SOFT OR REDUCIBLE)' TO J2.1), CALL THE FIELD SUPERVISOR TO LET THE PARTICIPANT PHYSICAL EXAMINADED]

Jpe.- PHYSICAL EXAMINATION GROIN [ONLY WHEN 'MASS (SOLID)', 'MASS (SOFT OR REDUCIBLE)' OR 'HERNIA' TO J2.1]

Patient should be examined while s/he is standing
Jpe1.1 – Scar
Is there a scar in the groin area?

	Problem 1
No	
Left	
Right	
Bilateral	

[In general the scar will be slightly cranially located in relation to the groin area. Note that the scar can have an oblique, transverse or longitudinal shape
bilateral: both sides of the body]

Jpe2.1 – Mass
Is there a mass in the groin area?

	Problem 1
No	
Left	
Right	
Bilateral	

[bilateral: both sides of the body]

Jpe2.2 – Mass during Valsalva manoeuvre
Is there a mass in the groin area during Valsalva manoeuvre?

	Problem 1
No	
Left	
Right	
Bilateral	

[bilateral: both sides of the body]

Jpe2.4 – Impression testis
What is the impression of the testis?

	Problem 1
Normal	
Atrophy	
Other	

Jpe3.1 – Clinical impression [multiple answers possible)
What is the clinical impression of the condition found?
[FILL IN THE OUTCOME OF THE PHYSICAL EXAMINATION ON THE LEFT SIDE OF THE PATIENT IN THE COLUMN 'LEFT' AND OUTCOME OF THE PHYSICAL EXAMINATION ON THE RIGHT SIDE OF THE PATIENT IN THE COLUMN 'RIGHT']
[Multiple answer possible]

	Left	Right
No condition found		
Suspected inguinal hernia		
Inguinal hernia, reduces spontaneously on lying down		
Inguinal hernia, reduces completely with gentle manipulation		

Inguinal hernia, inguinoscrotal, reducible with manual manipulation		
Inguinal hernia, irreducible		
Inguinal hernia, incarcerated/strangulated		
Suspected femoral hernia		
Femoral hernia, reduces spontaneously on lying down		
Femoral hernia, reduces completely with gentle manipulation		
Femoral hernia, inguinoscrotal, reducible with manual manipulation		
Femoral hernia, irreducible		
Femoral hernia, incarcerated/strangulated		
(Suspected) lipoma		
(Suspected) lymphadenopathy		
(Suspected) hydrocele		
Inexplicable		
Other, specify		

[Inguinal hernia: a part of the intestine or peritoneum pushes through a defect in the abdominal wall above the inguinal ligament
Femoral hernia: a part of the intestine or peritoneum pushes through a defect in the abdominal wall below the inguinal ligament
Lipoma: adipose/fat tissue that is mobile, soft to the touch, typically painless and present subcutaneously
Lymphadenopathy: enlargement of the inguinal lymph nodes
Hydrocele: abnormal accumulation of fluid in the pelvis, groin, labia majora or scrotum

Jpe3.2 – Circumference inguinoscrotal component *[only if at Jpe3.1 a hernia is detected]*
What is the circumference of the inguinoscrotal component?

	Problem 1
No inguinoscrotal component	
Circumference inguinoscrotal component < 10cm	
Circumference inguinoscrotal component 10 – 20 cm	
Circumference inguinoscrotal component > 20cm	
Other, specify	

Jpe4.1 – recommendation
As a health worker, what would you recommend the participant to do?
[FILL IN THE OUTCOME OF THE PHYSICAL EXAMINATION ON THE LEFT SIDE OF THE PATIENT IN THE COLUMN 'LEFT' AND OUTCOME OF THE PHYSICAL EXAMINATION ON THE RIGHT SIDE OF THE PATIENT IN THE COLUMN 'RIGHT']

	Left	Right
Neither watchful waiting or surgical hernia repair needed		
Watchful waiting		
Surgical hernia repair		
Recurrent groin hernia, referral to surgeon		
Surgical hernia repair not possible due to other disease/condition		
Other, specify		

Jpe5.1 – comments
Comments on physical examination of participant

	Problem 1
Comment	

[IF A GROIN HERNIA IS DIAGNOSED AND NEEDS TREATMENT, WRITE A REFFERAL LETTER FOR THE PARTICIPANT AND ADVICE THE PARTICIPANT IN WHICH CENTER HE OR SHE CAN BE TREATED]

[Only if man over 12yrs of age]

If you are all right I would like to continue to ask you questions specific for men and your peeing habits. Please tell us if you need clarification or if you want to stop. If you are not sure about the answer do not hesitate to tell us, this is important information as well. We will ask you to look at some pictures and ask you to point at the pictures that in most days is correct for your peeing habits.

J1.1 VPSS Visual scale

1. **How many times do you need to pee during daytime (when the sun is up) (1-6)**
2. **How many times do you need to pee during the night (when the sun is down) (1-6)**
3. **How does your peeing look like (1-5)**
4. **How much does the peeing affect your life (1-6)**

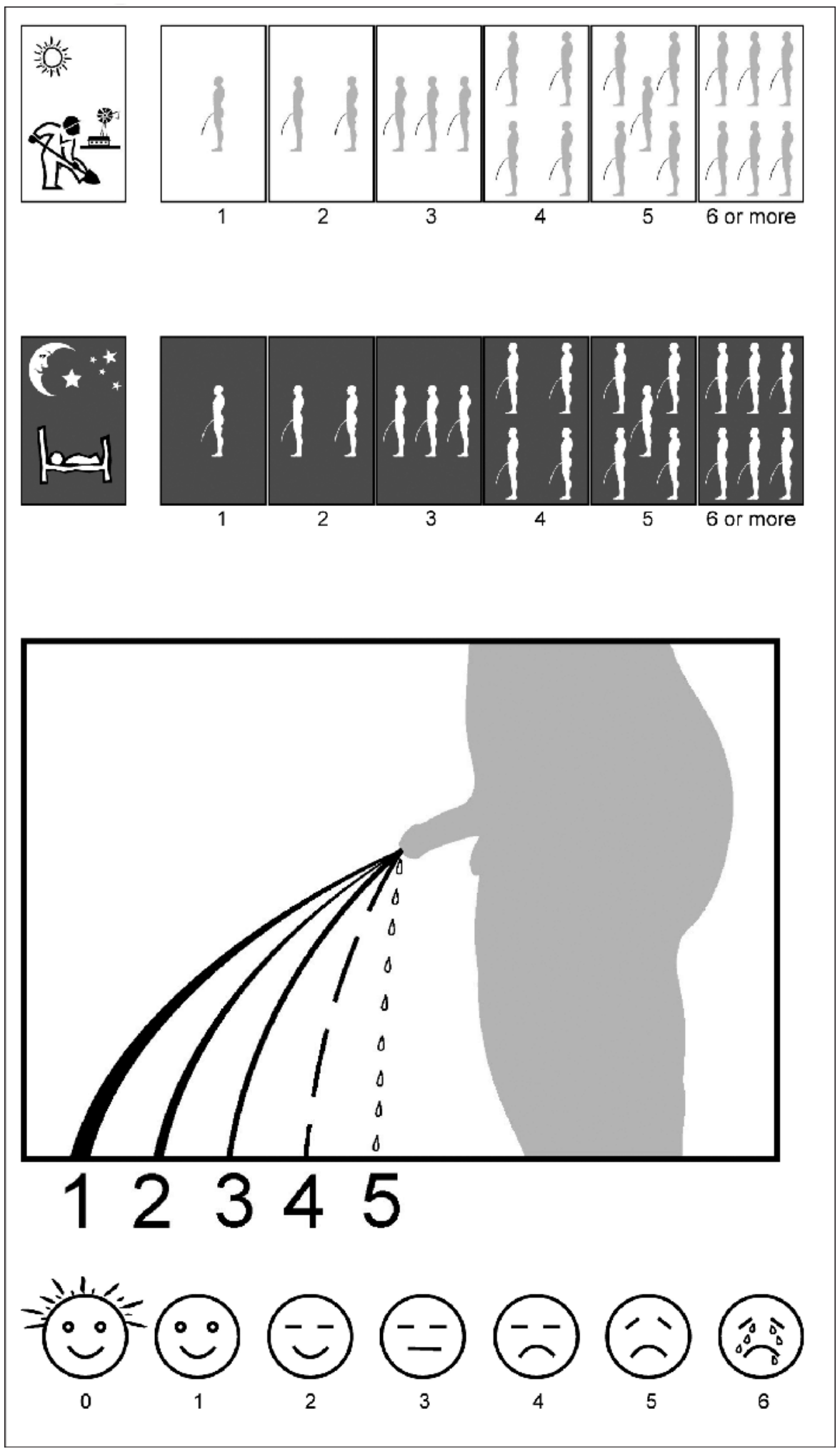

Present household member with VPSS. What is the outcome of the score? ___

If score is above 17, proceed to section JL.
[OFFER THE PARTICIPANT BAGS OF WATER]

[If the answer to JL1.1 is 17 or higher, continue with JL2.1. If the answer to JL1.1 is below the threshold OR the answer to JL1.1 is no, skip any further questions in section JL]

JL2.1. Timing:
For how long have you had this problem? ___months (if less than one month, write '0'. One year = 12 months)

	Problem 1
Number of months	
Unknown	

JL3.1. Healthcare sought:
Did you go to a health facility or see a doctor/nurse for this problem?

	Problem 1
Yes	
No	

JL4.1.1 Type of healthcare facility sought:
[Only when 'Yes' to JL3.1]
What kind of health facility did you seek healthcare?
DROPDOWN MENU FOR HEALTH CARE FACILITIES!!

JL5.1.1 Type of healthcare received:
[Only when 'Yes' to A4.1]
What kind of treatment did you receive?

	Problem 1
Major procedure	
Minor procedures	
Trans-urethral catheter	
Supra-pubic catheter	
No surgical care – medicines only	

No surgical care - no treatment offered	
Don't know	

[Major procedure: a procedure which requires regional/general anesthesia
Minor procedures: dressings, wound care, punctures, suturing and I&D
Trans-urethral catheter: Tube through the penis to drain urin
Supra-pubic catheter:] Tube through the abdomen to drain urin

JL5.1.3 Type of medical care received:
[Only when 'Yes' to "Trans-urethral catheter" in JL5.1.1]
What kind of Medicines did you receive?

	Severe LUTS
Antibiotics	
Other medications, namely…	
I do not know	

JL6.1.1 Traditional Healer:
Did you go to a traditional healer, traditional doctor, witch doctor or bone setter for this problem?

	Severe LUTS
Yes	
No	

JL6.1.2 Traditional healer - timing *[Only when JL6.1.1 is answered with yes]*

	Problem 1	Problem 2	Problem 3
Before going to a health care facility			
During treatment in a health care facility			
After treatment in a health care facility			
Unknown			

JL6.1.1 Type of health care received:
[Only when 'Yes' in A6.1.1]
What kind of treatment did you receive?

	Severe LUTS
Herbal treatment to eat/drink	
Herbal treatment to put on skin	
Surgical/invasive treatment	
Other	
Don't know	

JL7.1. Reason for not having surgical care:
[Only when 'no' to A4.1 or 'no surgical care' to A5.1]
What was the main reason not to go to a health facility to see a doctor/nurse or not to have an operation or dressings?
[If the person went to a traditional healer ask why (s)he didn't go to a health care facility and mark that as the answer.
If the person was referred but did not go to the referral hospital, mark the answer why (s)he did not go here.]

	Severe LUTS
No money for health care	
No (money for) transportation	
No time	
Fear / no trust	
Not available (facility/personnel/equipment)	
Traditional healer is treating me	
I feel ashamed	
I am currently saving money for a treatment	
No need (condition is not surgical)	
Other, namely…	
Unknown	

JL8.1. Disability:
Does this problem still impact your daily life?

	Severe LUTS
The condition is not disabling	
I feel ashamed	
I'm not able to work like I used to	
I need help with transportation	
I need physical help with daily living	

[this question is not meant in a financial way but rather if a patient needs physical help with transportation (wheelchair or walking stick or can not walk small distances) or daily living (such as getting out of bed, brushing teeth, eating]
[Disability: a physical problem that impacts your life, or makes it difficult to carry out your daily activities.]

JL 9 Penile discharge
Did you ever have penile discharge?
___Yes
___No

JL 9.1 Penile discharge [only if JL 2.2 is 'yes']
Have you been treated for penile discharge?

___Yes
___No

We detected that you have some problems with your peeing. I would like to ask you to pee in a plastic tube so that we can measure your peeing. If you do not got to pee now, please drink some more water. Please let me know when you have to pee, so we can do the measurment.

JL10 Uroflow
……….. ml/min
9 = not taken

1K. EXTREMITIES

K1. Extremities:
Have you ever had an injury, burn, wound, mass, deformity, broken bone, or an operation on your hands, feet, arms, or legs?
___ Yes
___ No
[If there were/are no problems with this anatomical section you can continue with the following. If the person had a problem with this anatomical section you question first on problem 1 all the questions on this page and go further if here are more problems]

K2.1. Extremity location:
On what part of your body did the problem occur?

	Problem 1	Problem 2	Problem 3
Finger(s)			
Thumb / Hand			
Forearm			
Upper Arm			
Foot			
Ankle inside			
Ankle outside			
Lower leg other			
Upper leg			

[If the problem is right and left: make two separate tabs to document the problem.
If the problem is based on a joint you need to choose the most proximate location to the abdomen.
For example: an elbow fracture is documented as the upper arm, a knee skin contracture is documented as the upper leg.]

K3.1. Extremity specifics:
Tell me what problem you have had.

	Problem 1	Problem 2	Problem 3
Wound injury related			
Wound not injury related			
(Recurrent) drainage / discharge			
Burn			
Mass growth			
Swelling of the whole limb			
Bone fracture or joint dislocation without a wound			
Deformity congenital			
Deformity acquired			

[Recurrent) drainage / discharge from small sore or opening in the skin: a high suspicion for osteomyelitis, infected bone
Deformity: An abnormal tissue arrangement, malformation
Congenital: The person is born with the problem. Think about: clubfeet
Acquired: The person got the problem later in life. Think about: broken bones and scar contractures]

K3.1.1 Type of injury / accident:
Did the problem start after an injury or accident? What kind of accident?
[Pick the one that best describes the injury / accident. Pedestrian and bicycle crash definition: there was no motorized vehicle involved. All can be intentional or unintentional.]

	Problem 1	Problem 2	Problem 3
No, it was not due to an injury / accident			
Car, truck, bus crash			
Motorcycle crash			
Pedestrian, bicycle crash			

Gunshot / firearm			
Stab / slash / cut / crush			
Bite or animal attack			
Fall			
Open fire / explosion			
Hot liquid / hot object			
None of the above			
Unknown			
Other, namely…			
Don't know			

K3.1. Timing:
When did this problem start?

	Problem 1	Problem 2	Problem 3
In the last month			
During the past 12 months but longer than a month ago			
Longer than 12 months ago			

K4.1.2 At this moment:
Do you have this problem now (or during the last week)?

	Problem 1	Problem 2	Problem 3
Yes			
No			

K4.2.1 Size: How large is your wound or burn? [only if wound or burn currently present]
[measure largest diameter of largest wounds in millimeters, 10 mm = 1 cm]

	Problem 1	Problem 2	Problem 3
Size in millimeters			

K4.2.2 Start of wound:
How did your wound start? *[only if wound not due to injury/accident]*

	Problem 1	Problem 2	Problem 3
Swelling around the wound			
Swelling of the whole limb			
Redness around the wound			
Bump (nodule)			
Less than one month of pus			
More than one month of pus			
Other, namely…			
Don't know			

K4.3.1 Initial treatment of burn: *[only if K3.1 is 'Burn']*
What was the first thing you did when you got the burn?

	Problem 1	Problem 2	Problem 3
washing with water			
covering with mud			
banana leaves			
papaya = pawpaw leaves			
papaya = pawpaw fruit			
raw eggs			
Salt			
Urine			
palm oil			
gentamycin violet			
aloe vera			
none			
Other, namely…			
Don't know			

K5.1. Fracture *[only if k3.1 is 'Wound injury related']:*
Did you break a bone or dislocate a joint?

	Problem 1	Problem 2	Problem 3
Yes			
No			

K6.1. Healthcare sought:
Did you go to a health facility or see a doctor/nurse for this problem?

	Problem 1	Problem 2	Problem 3
Yes			
No			

K6.1.1. Traditional Healer:
Did you go to a traditional healer, traditional doctor, witch doctor or bone setter for this problem?

	Problem 1	Problem 2	Problem 3
Yes			
No			

K6.1.2 Traditional healer - timing *[Only when K4.1.1 is answered with yes]*
When did you visit a traditional healer?

	Problem 1	Problem 2	Problem 3
Before visiting to a health care facility			
During treatment in a health care facility			
After visiting a health care facility			
Unknown			

K6.1.3 Traditional Healer: ***[only ifF4.1.1 is 'Yes' to one or more of the problems]***
How long did you visit a traditional healer, traditional doctor, witch doctor or bone setter for this problem? (months)

Problem 1	Problem 2	Problem 3

K7.1. Type of healthcare received: *[Only when 'Yes' to K6.1]*
What kind of treatment did you receive?

	Problem 1	Problem 2	Problem 3
None / No surgical care			
Major procedure			
Amputation			
Minor procedures			
Manipulation / casting / sling			
Traction			
Don't know			

[Major procedure: a procedure which requires regional or general anesthesia
Minor procedure: dressings, wound care, punctures, suturing and I&D
Internal / external fixation: the bone is fixed with an operation. Sometimes the metal needs to be removed afterwards.
Manipulation / casting / sling: the broken bone was immobilized for some time.
Traction: the patient needed to stay in a hospital bed for a long time, the broken limb was fixed to the bed or a heavy object.]

K7.1.1. Type of treatment received wounds: *[only if wound or burn present]*
What kind of treatment did you have for your wound or burn?

	Problem 1	Problem 2	Problem 3
Traditional medicine by mouth (to eat or drink or swallow)			
traditional medicine such as leaves or ointment			
modern medicine tablets (any oral drugs) self bought from a pharmacy or drug peddler			
modern medicine ointment / crème self bought from a pharmacy or drug peddler			
honey			
gentamycin violet (bright purple)			
X-ray			
Dressing at PHU			
Dressing at hospital			
Surgical debridement / operation by traditional healer			
Surgical debridement / operation by PHU or hospital			
Skin grafting			
None			
Other			
Don't know			

K8.1.3: Coverage of wound by skin graft *[only if K5.1.2 is 'Skin graft]*
Was your wound covered completely by the skin graft when you left the health facility?
___ Yes
___ No

[a skin graft is a surgery where healthy skin is taken (almost always from the upper leg) to cover a wound or bur)]

K8.1.4: Location skin graft *[only if K5.1.2 is 'Skin graft]*
Where was your skin graft done?
____ (name of health facility where skin graft was done)
DROPDOWN MENU

K9.1. Reason for not having surgical care: *[Only when 'none / no surgical care' to K7.1 or 'no' to K6.1]*
What was the main reason not to go to a health facility to see a doctor/nurse or not to have an operation or dressings?

	Problem 1	Problem 2	Problem 3
No money for health care			
No (money for) transportation			
No time			
Fear / no trust			
Not available (facility/personnel/equipment)			
Traditional healer is treating me			
I feel ashamed			
I am currently saving money for a treatment			
Because it was during the Ebola period			
No need (condition is not surgical)			
Other, namely…			
Unknown			

[If the person went to a traditional healer ask why (s)he didn't go to a health care facility and mark that as the answer. If the person was referred but did not go to the referral hospital, mark the answer why (s)he did not go here.]

K10.1. Disability:
Does this problem still impact your daily life?

	Problem 1	Problem 2	Problem 3
The condition is not disabling			
I feel ashamed			
I'm not able to work like I used to			
I need help with transportation			
I need physical help with daily living			

[this question is not meant in a financial way but rather if a patient needs physical help with transportation (wheelchair or walking stick or can not walk small distances) or daily living (such as getting out of bed, brushing teeth, eating]
[Disability: a physical problem that impacts your life, or makes it difficult to carry out your daily activities.]

[ASK IF THE PERSON HAD ANOTHER PROBLEM REGARDING THIS ANATOMICAL LOCATION. IF SO, GO TO THE FOLLOWING COLLUMN, PROBLEM 2 AND/OR 3, TO REGISTER THIS PROBLEM. IF HERE ARE NO OTHER PROBLEMS REGARDING TO THIS LOCATION YOU CAN GO TO THE FOLLOWING ANATOMICAL LOCATION. IN CASE OF MORE THAN 3 PROBLEMS, MARK THE ONES WHICH ARE MOST RESENT AND MOST RELEVANT FOR THE REPONDENT]

L. WOMEN'S HEALTH
[SECTION L ONLY IF WOMAN OF 12 YEARS OR OLDER]

Part L 1. Eligibility for Reproductive life survey
Part L 2. Gynecologic Complaints survey & Family Planning survey
Part L 3. Reproductive life survey & Pregnancy/Obstetrical History if applicable with perinatal death section
Part L 4. Perinatal death section

Part L 1. Eligibility for Reproductive life survey

L1.1 Gender[Are you a male?]
___ Yes
___ No
[IF 'YES'' YOU CAN SKIP ALL THE FOLLOWING QUESTIONS AND GO TO SECTION M; WOUNDS]

L.1.2. Age[Are you a girl below the age of 12 years?]
___ Yes
___ No
[IF 'YES'' YOU CAN SKIP ALL THE FOLLOWING QUESTIONS AND GO TO SECTION M; WOUNDS]

> I would like to continue to ask you questions specific for women and mothers. Please tell us if you need clarification or if you want to stop. This is of course allowed any time at any question. If you are not sure about the answer do not hesitate to tell us, this is important information as well. Do you have any questions on forehand?
>
> First we want to start with questions concerning your bleeding periods and family planning and later we want to ask you about your possible current and past pregnancies (Check if the woman agrees to that).

L.1.3 Reproductive Age
[Have you stopped seeing your bleeding periods for more than one year?]
____ Yes
____ Yes, but I'm pregnant or I could be pregnant now
____ Yes, my womb was removed during my last delivery and I am less than 50 years old
____ Yes, my womb was removed not pregnancy related and I am less than 50 years old
____ No

[IF 'answer is YES'' YOU CAN SKIP ALL THE FOLLOWING QUESTIONS AND GO TO SECTION M; WOUNDS
IF answer is "NO" , or "YES, BUT I'M PREGNANT OR I COULD BE PREGNANT"", go to question 2.1
IF answer is "YES, BUT MY WOMB WAS REMOVED DURING MY LAST DELIVERY" or "YES MY WOMB WAS REMOVED DUE TO

OTHER REASONS THAN PREGNANCY AND I AM LESS THAN 50 YEARS OLD" continue with question 3.1

Part L 2. Gynecologic Complaints survey & Family Planning survey

The following questions are about your menstrual period and family planning

L2.1. Length of period
How long does your period last? (number of days average)

_____
_____ Don't know

L2.2 Regularity
Does your period come regularly?
___ Yes
___ No
___ Don't know

L2.3 Intermittent bleeding
Do you have small bleedings in between your period?
___ Yes
___ No
___ Don't know

L2.4 Pads or towels/cloths
Do you use pads or towels/cloths or something else?
___ Pads
___ Towels/cloths
___ Tampons
___ Other; namely:

L2.5 Pads/towels:
How many sanitary pads/towels/tampons do you use on the heaviest day of your period? (number of pads/towels/tampons)

_____
_____ Don't know

L2.6 Pain:
Do you have pain during your period so that you cannot work?
___ Yes
___ No

L2.7.1 Health care needed
Do you need help for menstrual problems?
___ Yes
___ No

L2.7.1.1 Health care needed [*if L2.7.1 is yes]*
Did you search for help for menstrual problems?
___ Yes
___ No

[If answer is "Yes" continue with question L 2.7.2., If answer is "No" continue with question L 2.7.3.]

L2.7.2 Health care seeking behaviour *[only if L 2.7.1.1 is answered "Yes"]*
Where did you go with your menstrual problems?
___ Female family member
___ Traditional birth attendant
___ Traditional healer
___ Primary health care center (PHU)
___ Hospital
___ Other, please specify:

L2.7.3 Possibilities for health care *[only if L 2.7.1.1 is anwered "No"]*
Why did you not seek help for menstrual problems?
___ No, no need
___ No, no money for health care
___ No, no (money for) transportation
___ No, no time
___ No, because of fear / no trust
___ No, not available (facility/personnel/equipment)
___ Don't know

L2.8 Family Planning
Do you use a family planning method at the moment? *[Not including traditional methods]*
___ Yes [go to question L2.10]
___ No but I am currently pregnant [go to question L3.1]
___ No because I want to become pregnant now

___ No but I would like to use family planning [go to question L2.9]
___ No and I don't want to get pregnant now [go to question L2.9]
___ No because I have been operated so I cannot become pregnant anymore

L2.9 Possibilities for family planning *[only if L2.8 is answered No]*
Do you have the possibility to arrange family planning for yourself?
___ Yes, but no wish for family planning
___ No, no money for this
___ No, no transportation
___ No, no time
___ No, because of fear / no trust
___ No, not available (facility/personnel/equipment)
___ Other
___ Don't know

L2.10 Type of family planning *[only if L2.8 is answered with 'Yes']*
What method do you use currently?
___ Contraceptive pills
___ Implant
___ Injectable
___ Intra uterine device / coil
___ Condom
___ Other (surgical methods)
___ Don't know

L2.11 Duration of Family planning *[only if L2.8 is answered with 'Yes']*
Since how many years do you use this method? (number of years)
___ < 1 year
___ years

Part L 3. Reproductive life survey & Pregnancy/Obstetrical History if applicable

The following questions will be regarding previous pregnancies or your current pregnancy. As some pregnancies might be long ago, it might be difficult to remember but please try to answer as detailed as possible. If you do not know the details, then do not hesitate to say that you do not know.

L3.1 Gravida:
How many times, including the miscarriages and possible current pregnancy, have you been pregnant?
___ x amount
___ I don't know

[[IF '0', YOU CAN SKIP ALL THE FOLLOWING QUESTIONS AND GO TO SECTION WOUNDS: M]

L3.2 Pregnant:
Are you currently pregnant?
___ Yes
___ No
___ I don't know

[IF 'NO' OR 'I DON'T KNOW' YOU CAN SKIP ALL THE FOLLOWING QUESTIONS TILL QUESTION 3.3.1 ABOUT BREAST FEEDING AND OBSTETRICAL HISTORY.]

L3.2.1 Gestational Age: *[Only for those who are currently pregnant.]*
How many months are you pregnant?
___ x months
___ I don't know

L3.2.2 Complications in Pregnancy *[Only for those who are currently pregnant.]*
Have you had any problems during your current pregnancy?
___ Yes [if yes, go to question L3.2.3]
___ No [if no, go to question L3.2.4]

L3.2.3 Complications in Pregnancy
What kind of problem(s) do you have? *(multiple answers possible)*
___ Vaginal bleeding
___ Hypertension; high blood pressure
___ Malaria
___ Fever
___ Vaginal discharge
___ Urinary tract infection
___ Abdominal pain
___ Other, namely:

L3.2.3.1 Complications in Pregnancy (foetal)
Do you feel fetal movements / fetal kicks?

___ Yes
___ No

L3.2.4. Place of delivery (actual)
Where are you planning to deliver? *[Only for those who are currently pregnant.]*
___ Home
___ Hospital
___ Peripheral Health Unit (PHU)
___ Not decided yet

L3.2.5 Place of delivery (desired)
If you could choose yourself, where would you deliver? *[Only for those who are currently pregnant.]*
___ Home
___ Hospital
___ Peripheral Health Unit (PHU)
___ Other
___ Don't know yet

L3.2.6 Who takes the decision where you will deliver? *[Only for those who are currently pregnant.]*
___ Myself
___ Mother
___ Mother in law
___ Husband
___ Other person
___ Don't know

L3.2.7 Current pregnancy (gestation)

Measure fundal height (cm) in every pregnant women (ask consent first):

____ cm

For the Enumerator: If you wish to make a comment on these questions you can do this below:

____
____
____
____
____

> The following questions will be mostly about your past pregnancies. This can be pregnancies which resulted in a live new born, but also about possible miscarriages or still births. If you are ok with this, we would like to know more about all these past pregnancies and your deliveries. The first questions will be about your first pregnancy, then the same questions about your second, etc. We will start with two questions about breastfeeding than we will start with the past pregnancies.

L3.3.1 Breastfeeding:
Are you currently breastfeeding?
___ Yes
___ No

[IF 'NO' YOU CAN SKIP THE FOLLOWING QUESTIONS TILL QUESTION AND CONTINUE WITH THE OBSTETRICAL HISTORY QUESTION 1.2.4.]

L3.3.2 For how long/how many months are you breastfeeding?
___ Less than 3 months
___ Between 3-6 months
___ 6-9 months
___ 9 months and 1 year
___ More than 1 year
___ Don't know

Obstetrical History
[Same questions for every pregnancy the women had, start with the first pregnancy see table]

L3.4 How many pregnancies did you have? [encircle the amount in the table, for every pregnancy answer the question on the rows, start with first pregnancy first, fill all rows than continue to next column]

	1st pregnanc y	2nd pregnanc y	3rd pregnan y	4th pregnanc y	5th	6th pregnanc y	7th pregnanc y	8th pregnanc y	9th pregnanc y	10th pregnanc y

					pregnanc y					
3.4.1.										
3.4.2.										
3.4.3.										
3.4.4.										
3.4.5.										
3.4.6.										
3.4.7.										
3.4.8.										
3.5.1										
3.5.2										

3.4.1 Time [b,d or a] Type [1,2 or 3]

L3.4.1 Time of pregnancy
When did this pregnancy take place? [add answer in table column of correct pregnancy]
b = before Ebola period
d = during Ebola period
a = after Ebola period

3.4.2 Type [1,2 or 3]

L3.4.2 Type of pregnancy

How many babies were you pregnant of? [add answer in table column of correct pregnancy]
1 = singleton (1 baby)
2 = twin (2 babies)
3 = triplet (3 babies)
4 = unknown

3.4.3 Outcome [M,LM,A,S,D,E,U,O]

L3.4.3. Outcome of pregnancy
What was the outcome of this/your pregnancy? [Please choose the corresponding letter according to her answer]
M = Miscarriage; before 3 months of gestation
LM = Late Miscarriage; died between 3-6 months of gestation
A = Alive baby (in case of singleton) [indicate AA, if twin and both babies alive]
S = Stillbirth; Death baby (in case of singleton) in the womb; died before delivery [indicate SS, if twin and both babies died in the womb]
D = Baby died During delivery [indicate DD if twin and both babies died during delivery]
E = Baby died within 1 week of delivery; early neonatal Death [indicate EE if twin and both babies died within 1 week of delivery]
U = Unknown
O = Other

[Please check if she is still all right to continue] Can I ask you if you are still all right to continue? [If she lost any babies please tell her that you are sorry to hear this]. If the L3.4.2 indicates any perinatal death tell her that you want to ask her some questions about her deceased baby later if that is all right with her.]

[Only If answered with M or LM please go to question L3.4.9 and L3.4.10]

L3.4.4 Gestation [0,1 or 2]
At how many months did you deliver? [add answer in table column of correct pregnancy]
0 = before 9 (10) months
1 = around or at 9 (10) months
2 = after expected date
3 = Unknown

3.4.5 Place [V,P,H,O]

L3.4.5 Place of delivery
Where did the delivery take place?
V = Home
P = Primary health care center (PHU)
H = Hospital
O = Other

3.4.6 Mode [S,V,C]

L3.4.6 Mode of delivery
What was the mode of delivery?
S = spontaneous vaginal delivery
V = assisted delivery (Vacuum or forceps)
C = Caesarean Delivery / operation [If C go to *question 3.4.7]*

3.4.7 Indication if CS ***[B,BD,F,M,O,R,U]***

L3.4.7 Indication for CS
Do you recall the reason for the Caesarean Delivery / operation?
B = bleeding before birth
BD = bleeding during birth
F = fetal distress [for the baby]
M = Malpresentation
O = obstructed labour ['pelvis too small / baby too big']
R= Related to previous surgery (including CD)
U = unknown

3.4.8 Transportation **[A,B,C,F,H,M,P,T, O]**

L3.4.8 Transportation

What mode of transport did you use for this delivery?
A = Ambulance system
B = Boat
C = Carried
F = On foot
H = Animal
M= (Motor)cycle
P = Public transport (bus/taxi)
T = Car/Taxi
O = Other or no transport (in case of home delivery)

L 3.4.9 Did you have any of the following complaints before you had the miscarriage? *[Only ask this questions in case of a miscarriage (M or LM)]:*
___ Fever
___ Bleeding
___ Vaginal discharge
___ Abdominal pain
___ Other
___ Don't know

L 3.4.10 What do you think is the reason for the miscarriage?
___ Fever
___ Bleeding
___ Vaginal discharge
___ Abdominal pain
___ intentional abortion (due to medication, herbs or vaginal penetration)
___ Other; please specify
___ Don't know

L 3.4.11 Mode of intentional miscarriage/abortion *[Only if L3.4.10 answer was intentional abortion]*
What happened or did you take medicine/herbs to induce the miscarriage?
___ herbs
___ tablets (misoprostol) in the hospital
___ tablets (misoprostol) outside the hospital
___ vaginal penetration
___ other, namely:

L3.5.1 Complications after delivery or miscarriage?
Did any problems / complications occur after your delivery?
___ No
___ Yes, continue with question 3.5.2
___ I do not remember

Only if question 3.5.1 is answered 'yes'
L3.5.2 If Yes, what kind of problem / complication?
___ severe bleeding
___ infection/fever
___ perineal tear
___ wound infection
___ no menstrual period
___ different bleeding period
___ other; please specify
___ Unknown

For the Enumerator: If you wish to make a comment on these questions belonging to the table you can do this below:

____
____
____
____
____

If at question L3.4.3. Outcome of pregnancy at one pregnancy or more the answer was S,D or E please continue with section L4 Perinatal Death section.

*[IF ""***no S,D or E answered on L3.4.3***" YOU CAN SKIP ALL THE FOLLOWING QUESTIONS AND GO TO SECTION M, WOUNDS]*

Part L 4. Perinatal death section

[Please check if she is still all right to continue] I am very sorry to hear about the loss of your baby during pregnancy or delivery. Can I ask you if you are still all right to ask about what happened? You can tell me to stop whenever you want to.

[Please indicate which pregnancy/pregnancies you talk about and what the outcome of this pregnancy was? [Answer all questions in section L4 for every S,D or E answer the women gave]

L4.1 **Timing of stillbirth / perinatal death?**
Did one of the babies die less than 1 year ago?
___ Yes
___ No
___ I don't know

L4.2 Pregnancy / Sex and Outcome *[Only if L4.1 is answered with Yes]*

Please indicate which pregnancy [1-10]; Remember to include twins and triplets
What was the sex of the baby who passed away? [please indicate in table below: Male [M] or Female [F]
Outcome [repeat from question L 3.4.2, see beforere

Pregnancy (1-10)				
Para"How many children?				
Male (M) / Female (F)				
Outcome (see L3.4.2.)				

L4.3 *[Only if L4.1 is answered with Yes]*
Did you experience any of the following complaints during the end of your pregnancy or during the delivery of this baby?

	1	2TWin	3TRIPLE
Bleeding			
Fever/infection			
Prolonged/obstructed labour			
Breech presentation / Malpresentation			
Malaria			
Vaginal discharge			
Convulsions			
Previous illness for example Heart disease			
High blood pressure: Hypertension			
Other, namely			
Unknown			

[I want to check if you are still allright with me asking these question. Check with the woman how she feels and if she is allright to continue]

L4.4 Did the baby have any of the following problems less than a week before s/he died? *[Only ask this question if the answer the pregnancy outcome (L3.4.3) is 'E']*

	1…..	2…..	3…..
Fever			
Difficulty breathing			
Baby never cried			
Convulsions			
Not able to breastfeed			
Not able to urinate			

Not able to defecate			
Congenital birth defect			
Other, namely			
Unknown			

L4.5 Primary cause of perinatal death according to mother
What do you think could be the main reason for his/her death?
[For the Enumerator: Please give an explanation of the story told for every perinatal death if applicable.]

____
____
____
____

M. WOUNDS – IF ANY WOUNDS PRESENT IN PREVIOUS SECTIONS

M.1 Wound photographs. *[if any wounds present in any of the areas]*
Informed consent:
May we take photographs of your wound(s) and use them for expert consultation and publication in scientific journals and other media?
(*explanation to study subject, photographs will be anonymized as much as possible and are stored on a password protected system)*
___ Yes
___No (If no, what is the reason? _____________)
(no time / no willingness / no reason / no seen benefit / other: explain….

M.2 Duration of wound
How many months do you have your wound? If there are multiple wounds, choose the one that is there already the longest.
___ months

M.3 Exposure to stagnant water
How often in the week are you working with your feet in stagnant water?
___ never
___1-2 days/week
___ 3-4 days/week
___ 5-6 days/week
___ 7 days/week

M.4 Pain at the wound site
How painful is your wound? (if multiple wounds, choose the one that is most painful)
___ (0 = not painful, 2 = hurts little bit, 4 = hurts little more, 6 = hurts even more 8 = hurts whole lot, 10 = hurts worst)

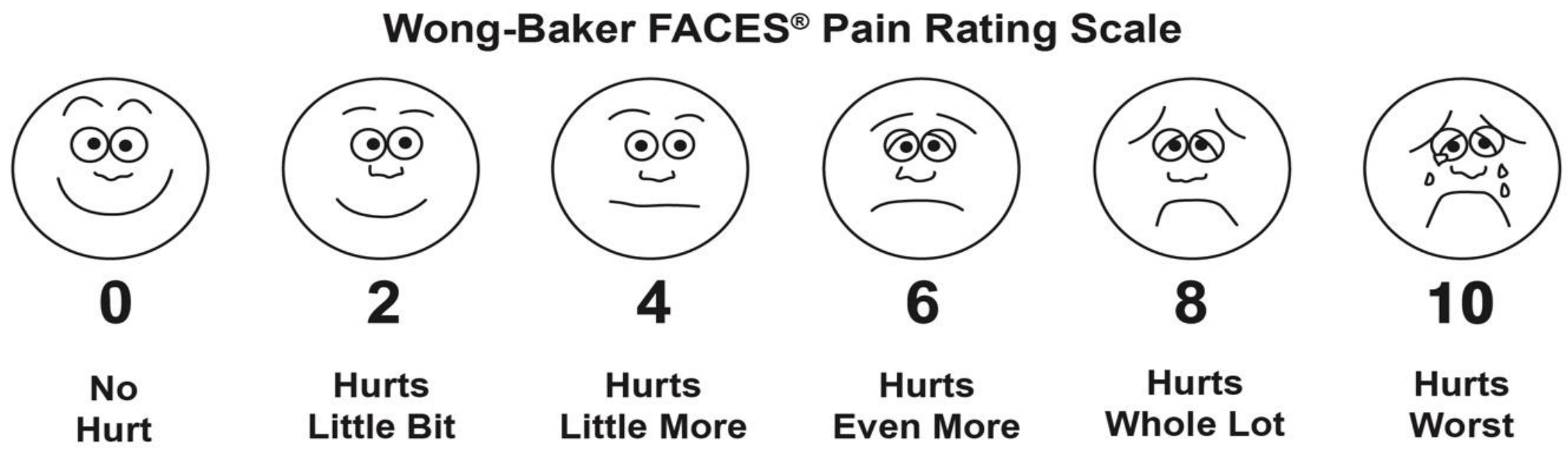

©1983 Wong-Baker FACES Foundation. www.WongBakerFACES.org
Used with permission.

Instructions for Usage

Explain to the person that each face represents a person who has no pain (hurt), or some, or a lot of pain.

Face 0 doesn't hurt at all. Face 2 hurts just a little bit. Face 4 hurts a little bit more. Face 6 hurts even more. Face 8 hurt a whole lot. Face 10 hurts as much as you can imagine, although you don't have to be crying to have this worst pain.

Ask the person to choose the face that best depicts the pain they are experiencing.

M.4.1 Painkillers for wound
How often in the week do you take painkillers for your wound?
___ never
___ 1-2 days/week
___ 3-4 days/week
___ 5-6 days/week
___ 7 days/week

M.5 Working with wounds
Is your wound/ are your wounds preventing you from working?
___ (1 = not at all, 2 = not often, 3 = sometimes, 4 = often, 5 = always)

Thank you very much for answering these questions. Let me check everything.

[Go over all the tabs to ensure that you have everything. Ask the questions again which you accidentally skipped.

I checked the full Survey, there is no data missing:
______________ *[date]* _______________ *[name]* _______________ *[signature of interviewer]*

I see that I have all the answers to the questions that I need; your survey is complete. My college will take some measurements. She/he will measure your blood pressure. She/he will tell you more about it. Thanks again on behalf of the research team. Do you have questions for me? You are always welcome to contact us. Have a nice day.

[Give or refer to the letter with the (contact) information about the survey.] [TIME: ____ hour ____ min]

If you wish to make a comment for this interview you can do that below:
____
____
____
____

N. Measurements

[THIS SECTION IS ONLY FOR PARTICIPANTS THAT ARE 18 YEAR OR OLDER. THIS SECTION SHOULD BE SKIPPED FOR EACH PARTICIPANT THAT IS 17 YEARS OLD OR YOUNGER]

My college will take some measurements. She/he will take your blood pressure. She/he will tell you more about it.

[THE FIELD SUPERVISOR, THE DATA MANAGER OR THE DRIVER WILL NOW TAKE THE BLOOD PRESSURE OF THE PARTICPANT. ONCE THEY HAVE FINISHED THE MEASUREMENTS, THEY WILL INFORM YOU ABOUT THE RESULTS. ONLY AFTER YOU HAVE RECEIVED THE RESULTS OF THE BLOOD PRESSURE MEASUREMENT FROM THE FIELD SUPERVISOR, THE DATA MANAGER OR THE DRIVER YOU CAN FINISH THE SURVEY. IN THE MEAN TIME YOU CAN START TAKING THE SURVEY OF THE OTHER HOUSEHOLD MEMBER OR GO TO THE NEXT HOUSEHOLD. HAND THE PARTICIPANT OVER TO THE FIELD SUPERVISOR, THE DATA MANAGER OR THE DRIVER]

[PARTICIPANT IS TAKEN OVER BY THE FIELD SUPERVISOR, THE DATA MANAGER OR THE DRIVER]

N0 – preparation of blood pressure measurement

[ALWAYS BE RESPECTFULL TOWARDS THE PARTICIPANT. MAKE SURE THE PARTICIPANT FEELS COMFORTABLE. UNLESS THE PARTICIPANT DO NOT WANT TO, TAKE THE MEASUREMENTS IN A COMPLETELY PRIVATE PLACE JUST LIKE THE REST OF THE INTERVIEW. KINDLY ASK THE PARTICPANT TO REMOVE EXCESS CLOTHING AND HEAVY ACCESSORIES, SUCH AS SHOES, COATS,SCARVES, HEAVY JEWELRY, COINS, CELL PHONES OR WALLETS BEFORE THE MEASURMENTS. MAKE SURE THAT THE LEFT ARM IS NOT TIGHTLY COVERED FOR MEASURING THE BLOOD PRESSURE]

Good morning/afternoon/evening, today I am assisting the research nurse that asked you all questions. I would like to measure your blood pressure and your heart rate. Your blood pressure can be healthy, too low or too high. If the blood pressure is too low or too high, doctors and health care workers can help you with establishing a healthy blood pressure. To measure your blood pressure I will put this cuff around your arm and ask you to follow my instructions. I will take you blood pressure three times in total. Between two measurements we will wait for 2 to 3 minutes. Can I proceed or do you have any questions?

N1 – Measuring the blood pressure and the heart rate

[FOLLOW THE STAPS AS DESCRIBE BELOW TO MEASURE THE BLOOD PRESSURE AND THE HEART RATE:
- THE PARTICIPANT HAS TO BE SEATED WITH UNCROSSED LEGS AND RELAXED FOR 5 MINUTES BEFORE THE FIRST BLOOD PRESSURE READING IS TAKEN. IN CASE THE PARTICIPANT HAS BEEN SEATED THROUGH THE WHOLE INTERVIEW YOU CAN MOVE TO THE FIRST MEASURMENT WITHOUT WAITING THOSE 5 MINUTES]
- PLACE THE LEFT ARM OF THE PARTICIPANTS SLIGHTLY BENT AND WITH THE PALM FACING UPWARDS
- PUT THE BLOOD PRESSURE MEASUREMENT INSTRUMENT AROUND THE LEFT WRIST OF THE PARTICPANT WITH THE INSTRUMENT IN THE INTERIOR SIDE OF THE ARM
- ASSURE THAT THE BLOOD PRESSURE MEASUREMENT INSTRUMENT IS TIGHTLY PLACED ON THE WRIST
- ASSURE THAT THE ARM DOES NOT MOVE AND ASK THE PERSON NOT TO TALK WHILE TAKING THE MEASUREMENT
- PRESS THE START BUTTON
- WAIT UNTIL THE BLOOD PRESSURE MEASUREMENT INSTRUMENT SHOWS THE THREE RESULTS
  - DIA
  - SYS
  - RATE
- COPY THE RESULT IN THE TABLE BELOW
- SWITCH OFF THE BLOOD PRESSURE MEASUREMENT INSTRUMENT
- WAIT FOR 2 TO 3 MINUTES BEFORE TAKEN THE SECOND MEASUREMENT OF BLOOD PRESSURE. INSTRUCT THE PARTICIPANT TO KEEP SEATED DURING THIS PERIOD.
- PRESS THE START BUTTON AGAIN
- TAKE THE THREE NUMBERS AGAIN AND COPY THE RESULT IN THE TABLE BELOW
- SWITCH OFF THE BLOOD PRESSURE MEASUREMENT INSTRUEMENT
- WAIT FOR 2 TO 3 MINUTES BEFORE TAKEN THE SECOND MEASUREMENT OF BLOOD PRESSURE. INSTRUCT THE

PARTICIPANT TO KEEP SEATED DURING THIS PERIOD.

- PRESS THE START BUTTON AGAIN
- TAKE THE THREE NUMBERS AGAIN AND COPY THE RESULT IN THE TABLE BELOW
- CALL THE FIELD SUPERVISOR WHEN THE SECOND AND THE THIRD MEASURMENT OF THE BLOOD PRESSURE HAVE EITHER:
  - A DIASTOLIC (DIA) LEVEL THAT IS HIGHER THAN 90 mmHg
  - OR A SYSTOLIC (SYS) LEVEL THAT IS HIGHER THAN 140 mmHg
- THANK THE PARTICIPANT FOR THEIR COOPERATION AND THEIR TIME

BLOOD PRESSURE & PULSE				
Reading 1: Systolic (SYS)	**(Answer in mm Hg)**			
Reading 1: Diastolic (DIA)	**(Answer in mm Hg)**			
Reading 1: Pulse	**(Beats per minute)**			
Reading 2: Systolic (SYS)	**(Answer in mm Hg)**			
Reading 2: Diastolic (DIA)	**(Answer in mm Hg)**			
Reading 2: Pulse	**(Beats per minute)**			
Reading 3: Systolic (SYS)	**(Answer in mm Hg)**			
Reading 3: Diastolic (DIA)	**(Answer in mm Hg)**			
Reading 3: Pulse	**(Beats per minute)**			

REPEAT SECTIONS E-N FOR SECOND RESPONDENT
